# Diretriz Brasileira de Cardio-oncologia – 2020

**DOI:** 10.36660/abc.20201006

**Published:** 2020-11-01

**Authors:** Ludhmila Abrahão Hajjar, Isabela Bispo Santos da Silva da Costa, Marcelo Antônio Cartaxo Queiroga Lopes, Paulo Marcelo Gehm Hoff, Maria Del Pilar Estevez Diz, Silvia Moulin Ribeiro Fonseca, Cristina Salvadori Bittar, Marília Harumi Higuchi dos Santos Rehder, Stephanie Itala Rizk, Dirceu Rodrigues Almeida, Gustavo dos Santos Fernandes, Luís Beck-da-Silva, Carlos Augusto Homem de Magalhães Campos, Marcelo Westerlund Montera, Sílvia Marinho Martins Alves, Júlia Tizue Fukushima, Maria Verônica Câmara dos Santos, Carlos Eduardo Negrão, Thiago Liguori Feliciano da Silva, Silvia Moreira Ayub Ferreira, Marcus Vinicius Bolivar Malachias, Maria da Consolação Vieira Moreira, Manuel Maria Ramos Valente, Veronica Cristina Quiroga Fonseca, Maria Carolina Feres de Almeida Soeiro, Juliana Barbosa Sobral Alves, Carolina Maria Pinto Domingues Carvalho Silva, João Sbano, Ricardo Pavanello, Ibraim Masciarelli F. Pinto, Antônio Felipe Simão, Marianna Deway Andrade Dracoulakis, Ana Oliveira Hoff, Bruna Morhy Borges Leal Assunção, Yana Novis, Laura Testa, Aristóteles Comte de Alencar, Cecília Beatriz Bittencourt Viana Cruz, Juliana Pereira, Diego Ribeiro Garcia, Cesar Higa Nomura, Carlos Eduardo Rochitte, Ariane Vieira Scarlatelli Macedo, Patricia Tavares Felipe Marcatti, Wilson Mathias, Evanius Garcia Wiermann, Renata do Val, Helano Freitas, Anelisa Coutinho, Clarissa Maria de Cerqueira Mathias, Fernando Meton de Alencar Camara Vieira, André Deeke Sasse, Vanderson Rocha, José Antônio Franchini Ramires, Roberto Kalil

**Affiliations:** 1 Universidade de São Paulo Faculdade de Medicina Instituto do Coração São PauloSP Brasil Instituto do Coração do Hospital das Clínicas da Faculdade de Medicina da Universidade de São Paulo, São Paulo, SP – Brasil; 2 Universidade de São Paulo Faculdade de Medicina Instituto do Câncer Hospital das Clínicas São PauloSP Brasil Instituto do Câncer Hospital das Clínicas da Faculdade de Medicina da Universidade de São Paulo, São Paulo, SP – Brasil; 3 Hospital Alberto Urquiza Wanderley João PessoaPB Brasil Hospital Alberto Urquiza Wanderley, João Pessoa, PB – Brasil; 4 Instituto D'or Pesquisa e Ensino Rio de JaneiroRJ Brasil Instituto D'or Pesquisa e Ensino, Rio de Janeiro, RJ – Brasil; 5 Hospital Sírio Libanês São PauloSP Brasil Hospital Sírio Libanês, São Paulo, SP – Brasil; 6 Universidade Federal de São Paulo São PauloSP Brasil Universidade Federal de São Paulo (UNIFESP), São Paulo, SP – Brasil; 7 Hospital de Clínicas de Porto Alegre Porto AlegreRS Brasil Hospital de Clínicas de Porto Alegre, Porto Alegre, RS – Brasil; 8 Universidade Federal do Rio Grande do Sul Porto AlegreRS Brasil Universidade Federal do Rio Grande do Sul, Porto Alegre, RS – Brasil; 9 Hospital Pró-Cardíaco Rio de JaneiroRJ Brasil Hospital Pró-Cardíaco, Rio de Janeiro, RJ – Brasil; 10 Pronto Socorro Cardiológico de Pernambuco RecifePE Brasil Pronto Socorro Cardiológico de Pernambuco (PROCAPE), Recife, PE – Brasil; 11 Sociedade Brasileira de Oncologia Pediátrica São PauloSP Brasil Sociedade Brasileira de Oncologia Pediátrica (SOBOPE), São Paulo, SP – Brasil; 12 Sociedade Brasileira de Cardiologia Departamento de Cardiopatias Congênitas e Cardiologia Pediátrica Rio de JaneiroRJ Brasil Departamento de Cardiopatias Congênitas e Cardiologia Pediátrica (DCC/CP) da Sociedade Brasileira de Cardiologia (SBC), Rio de Janeiro, RJ – Brasil; 13 Faculdade de Ciências Médicas de Minas Gerais Belo HorizonteMG Brasil Faculdade de Ciências Médicas de Minas Gerais, Belo Horizonte, MG – Brasil; 14 Universidade Federal de Minas Gerais Belo HorizonteMG Brasil Universidade Federal de Minas Gerais, Belo Horizonte, MG – Brasil; 15 Hospital do Coração da Associação do Sanatório Sírio – HCor São PauloSP Brasil Hospital do Coração da Associação do Sanatório Sírio – HCor, São Paulo, SP – Brasil; 16 Instituto Dante Pazzanese de Cardiologia São PauloSP Brasil Instituto Dante Pazzanese de Cardiologia, São Paulo, SP – Brasil; 17 Instituto de Cardiologia de Santa Catarina São JoséSC Brasil Instituto de Cardiologia de Santa Catarina, São José, SC – Brasil; 18 Hospital da Bahia SalvadorBA Brasil Hospital da Bahia, Salvador, BA – Brasil; 19 Universidade Federal do Amazonas ManausAM Brasil Universidade Federal do Amazonas, Manaus, AM – Brasil; 20 Hospital do Coração São PauloSP Brasil Hospital do Coração (HCOR), São Paulo, SP – Brasil; 21 Santa Casa de Misericórdia de São Paulo São PauloSP Brasil Santa Casa de Misericórdia de São Paulo, São Paulo, SP – Brasil; 22 Rede Mater Dei de Saúde Belo HorizonteMG Brasil Rede Mater Dei de Saúde, Belo Horizonte, MG – Brasil; 23 Centro de Oncologia do Paraná CuritibaPR Brasil Centro de Oncologia do Paraná, Curitiba, PR – Brasil; 24 A.C. Camargo Cancer Center São PauloSP Brasil A.C. Camargo Cancer Center, São Paulo, SP – Brasil; 25 Hospital Aliança SalvadorBA Brasil Hospital Aliança, Salvador, BA – Brasil; 26 NOB/Oncoclínicas SalvadorBA Brasil NOB/Oncoclínicas, Salvador, BA – Brasil; 27 Sociedade Brasileira de Oncologia Clínica São PauloSP Brasil Sociedade Brasileira de Oncologia Clínica, São Paulo, SP – Brasil; 28 Grupo Americas Oncologia Rio de JaneiroRJ Brasil Grupo Americas Oncologia, Rio de Janeiro, RJ – Brasil; 29 Grupo SOnhe CampinasSP Brasil Grupo SOnhe, Campinas, SP – Brasil; 30 Universidade Estadual de Campinas CampinasSP Brasil Universidade Estadual de Campinas (Unicamp), Campinas, SP – Brasil

**Table:** 

Diretriz Brasileira de Cardio-oncologia – 2020	
O relatório abaixo lista as declarações de interesse conforme relatadas à SBC pelos especialistas durante o período de desenvolvimento desta diretriz, 2020.	
Especialista	Tipo de relacionamento com a indústria
Ana Oliveira Hoff	DECLARAÇÃO FINANCEIRA A - PAGAMENTO DE QUALQUER ESPÉCIE E DESDE QUE ECONOMICAMENTE APRECIÁVEIS, FEITOS A (i) VOCÊ, (ii) AO SEU CÔNJUGE/COMPANHEIRO OU A QUALQUER OUTRO MEMBRO QUE RESIDA COM VOCÊ, (iii) A QUALQUER PESSOA JURÍDICA EM QUE QUALQUER DESTES SEJA CONTROLADOR, SÓCIO, ACIONISTA OU PARTICIPANTE, DE FORMA DIRETA OU INDIRETA, RECEBIMENTO POR PALESTRAS, AULAS, ATUAÇÃO COMO PROCTOR DE TREINAMENTOS, REMUNERAÇÕES, HONORÁRIOS PAGOS POR PARTICIPAÇÕES EM CONSELHOS CONSULTIVOS, DE INVESTIGADORES, OU OUTROS COMITÊS, ETC. PROVENIENTES DA INDÚSTRIA FARMACÊUTICA, DE ÓRTESES, PRÓTESES, EQUIPAMENTOS E IMPLANTES, BRASILEIRAS OU ESTRANGEIRAS: - Bayer: câncer de tireoide - Exelixis: câncer de tireoide - Eli Lilly: câncer de tireoide - United B - FINANCIAMENTO DE PESQUISAS SOB SUA RESPONSABILIDADE DIRETA/PESSOAL (DIRECIONADO AO DEPARTAMENTO OU INSTITUIÇÃO) PROVENIENTES DA INDÚSTRIA FARMACÊUTICA, DE ÓRTESES, PRÓTESES, EQUIPAMENTOS E IMPLANTES, BRASILEIRAS OU ESTRANGEIRAS: - Exelixis: câncer de tireoide - Eli Lilly: câncer de tireoide
André Deeke Sasse	DECLARAÇÃO FINANCEIRA A - PAGAMENTO DE QUALQUER ESPÉCIE E DESDE QUE ECONOMICAMENTE APRECIÁVEIS, FEITOS A (i) VOCÊ, (ii) AO SEU CÔNJUGE/COMPANHEIRO OU A QUALQUER OUTRO MEMBRO QUE RESIDA COM VOCÊ, (iii) A QUALQUER PESSOA JURÍDICA EM QUE QUALQUER DESTES SEJA CONTROLADOR, SÓCIO, ACIONISTA OU PARTICIPANTE, DE FORMA DIRETA OU INDIRETA, RECEBIMENTO POR PALESTRAS, AULAS, ATUAÇÃO COMO PROCTOR DE TREINAMENTOS, REMUNERAÇÕES, HONORÁRIOS PAGOS POR PARTICIPAÇÕES EM CONSELHOS CONSULTIVOS, DE INVESTIGADORES, OU OUTROS COMITÊS, ETC. PROVENIENTES DA INDÚSTRIA FARMACÊUTICA, DE ÓRTESES, PRÓTESES, EQUIPAMENTOS E IMPLANTES, BRASILEIRAS OU ESTRANGEIRAS: - Astellas: oncologia - Bayer: oncologia - Janssen: oncologia - Merck Serono - MSD - Novartis - Roche OUTROS RELACIONAMENTOS FINANCIAMENTO DE ATIVIDADES DE EDUCAÇÃO MÉDICA CONTINUADA, INCLUINDO VIAGENS, HOSPEDAGENS E INSCRIÇÕES PARA CONGRESSOS E CURSOS, PROVENIENTES DA INDÚSTRIA FARMACÊUTICA, DE ÓRTESES, PRÓTESES, EQUIPAMENTOS E IMPLANTES, BRASILEIRAS OU ESTRANGEIRAS: - MSD: oncologia - Janssen: oncologia - Novartis: oncologia
Anelisa Coutinho	DECLARAÇÃO FINANCEIRA A - PAGAMENTO DE QUALQUER ESPÉCIE E DESDE QUE ECONOMICAMENTE APRECIÁVEIS, FEITOS A (i) VOCÊ, (ii) AO SEU CÔNJUGE/COMPANHEIRO OU A QUALQUER OUTRO MEMBRO QUE RESIDA COM VOCÊ, (iii) A QUALQUER PESSOA JURÍDICA EM QUE QUALQUER DESTES SEJA CONTROLADOR, SÓCIO, ACIONISTA OU PARTICIPANTE, DE FORMA DIRETA OU INDIRETA, RECEBIMENTO POR PALESTRAS, AULAS, ATUAÇÃO COMO PROCTOR DE TREINAMENTOS, REMUNERAÇÕES, HONORÁRIOS PAGOS POR PARTICIPAÇÕES EM CONSELHOS CONSULTIVOS, DE INVESTIGADORES, OU OUTROS COMITÊS, ETC. PROVENIENTES DA INDÚSTRIA FARMACÊUTICA, DE ÓRTESES, PRÓTESES, EQUIPAMENTOS E IMPLANTES, BRASILEIRAS OU ESTRANGEIRAS: - Bayer: oncologia - Amgen: oncologia - Roche: oncologia - Merck group - MSD - Lilly - Servier B - FINANCIAMENTO DE PESQUISAS SOB SUA RESPONSABILIDADE DIRETA/PESSOAL (DIRECIONADO AO DEPARTAMENTO OU INSTITUIÇÃO) PROVENIENTES DA INDÚSTRIA FARMACÊUTICA, DE ÓRTESES, PRÓTESES, EQUIPAMENTOS E IMPLANTES, BRASILEIRAS OU ESTRANGEIRAS: - Bristol: oncologia - Servier: oncologia OUTROS RELACIONAMENTOS FINANCIAMENTO DE ATIVIDADES DE EDUCAÇÃO MÉDICA CONTINUADA, INCLUINDO VIAGENS, HOSPEDAGENS E INSCRIÇÕES PARA CONGRESSOS E CURSOS, PROVENIENTES DA INDÚSTRIA FARMACÊUTICA, DE ÓRTESES, PRÓTESES, EQUIPAMENTOS E IMPLANTES, BRASILEIRAS OU ESTRANGEIRAS: - Roche: oncologia - Servier: oncologia - Bayer: oncologia - Merck group - Amgen - Sanofi
Antônio Felipe Simão	DECLARAÇÃO FINANCEIRA A - PAGAMENTO DE QUALQUER ESPÉCIE E DESDE QUE ECONOMICAMENTE APRECIÁVEIS, FEITOS A (i) VOCÊ, (ii) AO SEU CÔNJUGE/COMPANHEIRO OU A QUALQUER OUTRO MEMBRO QUE RESIDA COM VOCÊ, (iii) A QUALQUER PESSOA JURÍDICA EM QUE QUALQUER DESTES SEJA CONTROLADOR, SÓCIO, ACIONISTA OU PARTICIPANTE, DE FORMA DIRETA OU INDIRETA, RECEBIMENTO POR PALESTRAS, AULAS, ATUAÇÃO COMO PROCTOR DE TREINAMENTOS, REMUNERAÇÕES, HONORÁRIOS PAGOS POR PARTICIPAÇÕES EM CONSELHOS CONSULTIVOS, DE INVESTIGADORES, OU OUTROS COMITÊS, ETC. PROVENIENTES DA INDÚSTRIA FARMACÊUTICA, DE ÓRTESES, PRÓTESES, EQUIPAMENTOS E IMPLANTES, BRASILEIRAS OU ESTRANGEIRAS: - AstraZeneca: cardiologia OUTROS RELACIONAMENTOS FINANCIAMENTO DE ATIVIDADES DE EDUCAÇÃO MÉDICA CONTINUADA, INCLUINDO VIAGENS, HOSPEDAGENS E INSCRIÇÕES PARA CONGRESSOS E CURSOS, PROVENIENTES DA INDÚSTRIA FARMACÊUTICA, DE ÓRTESES, PRÓTESES, EQUIPAMENTOS E IMPLANTES, BRASILEIRAS OU ESTRANGEIRAS: - AstraZeneca - Bayer
Ariane Vieira Scarlatelli Macedo	DECLARAÇÃO FINANCEIRA A - PAGAMENTO DE QUALQUER ESPÉCIE E DESDE QUE ECONOMICAMENTE APRECIÁVEIS, FEITOS A (i) VOCÊ, (ii) AO SEU CÔNJUGE/COMPANHEIRO OU A QUALQUER OUTRO MEMBRO QUE RESIDA COM VOCÊ, (iii) A QUALQUER PESSOA JURÍDICA EM QUE QUALQUER DESTES SEJA CONTROLADOR, SÓCIO, ACIONISTA OU PARTICIPANTE, DE FORMA DIRETA OU INDIRETA, RECEBIMENTO POR PALESTRAS, AULAS, ATUAÇÃO COMO PROCTOR DE TREINAMENTOS, REMUNERAÇÕES, HONORÁRIOS PAGOS POR PARTICIPAÇÕES EM CONSELHOS CONSULTIVOS, DE INVESTIGADORES, OU OUTROS COMITÊS, ETC. PROVENIENTES DA INDÚSTRIA FARMACÊUTICA, DE ÓRTESES, PRÓTESES, EQUIPAMENTOS E IMPLANTES, BRASILEIRAS OU ESTRANGEIRAS: - Bayer: anticoagulantes - Pfizer: anticoagulantes - Daichii Sankyo: anticoagulantes - AstraZeneca: anticoagulantes OUTROS RELACIONAMENTOS FINANCIAMENTO DE ATIVIDADES DE EDUCAÇÃO MÉDICA CONTINUADA, INCLUINDO VIAGENS, HOSPEDAGENS E INSCRIÇÕES PARA CONGRESSOS E CURSOS, PROVENIENTES DA INDÚSTRIA FARMACÊUTICA, DE ÓRTESES, PRÓTESES, EQUIPAMENTOS E IMPLANTES, BRASILEIRAS OU ESTRANGEIRAS: - Bayer: anticoagulantes - Pfizer: anticoagulantes - Zodiac: quimioterápicos - Ferring
Aristóteles Comte de Alencar Filho	DECLARAÇÃO FINANCEIRA A - PAGAMENTO DE QUALQUER ESPÉCIE E DESDE QUE ECONOMICAMENTE APRECIÁVEIS, FEITOS A (i) VOCÊ, (ii) AO SEU CÔNJUGE/COMPANHEIRO OU A QUALQUER OUTRO MEMBRO QUE RESIDA COM VOCÊ, (iii) A QUALQUER PESSOA JURÍDICA EM QUE QUALQUER DESTES SEJA CONTROLADOR, SÓCIO, ACIONISTA OU PARTICIPANTE, DE FORMA DIRETA OU INDIRETA, RECEBIMENTO POR PALESTRAS, AULAS, ATUAÇÃO COMO PROCTOR DE TREINAMENTOS, REMUNERAÇÕES, HONORÁRIOS PAGOS POR PARTICIPAÇÕES EM CONSELHOS CONSULTIVOS, DE INVESTIGADORES, OU OUTROS COMITÊS, ETC. PROVENIENTES DA INDÚSTRIA FARMACÊUTICA, DE ÓRTESES, PRÓTESES, EQUIPAMENTOS E IMPLANTES, BRASILEIRAS OU ESTRANGEIRAS: - Novartis: Entresto - Sandoz: Pidezot
Bruna Morhy Borges Leal Assunção	Nada a ser declarado
Carlos Augusto Homem de Magalhães Campos	Nada a ser declarado
Carlos Eduardo Negrão	Nada a ser declarado
Carlos Eduardo Rochitte	Nada a ser declarado
Carolina Maria Pinto Domingues Carvalho Silva	Nada a ser declarado
Cecilia Beatriz Bittencourt Viana Cruz	Nada a ser declarado
Cesar Higa Nomura	Nada a ser declarado
Clarissa Maria de Cerqueira Mathias	Nada a ser declarado
Cristina Salvadori Bittar	Nada a ser declarado
Diego Ribeiro Garcia	Nada a ser declarado
Dirceu Rodrigues Almeida	Nada a ser declarado
Evanius Garcia Wiermann	DECLARAÇÃO FINANCEIRA A - PAGAMENTO DE QUALQUER ESPÉCIE E DESDE QUE ECONOMICAMENTE APRECIÁVEIS, FEITOS A (i) VOCÊ, (ii) AO SEU CÔNJUGE/COMPANHEIRO OU A QUALQUER OUTRO MEMBRO QUE RESIDA COM VOCÊ, (iii) A QUALQUER PESSOA JURÍDICA EM QUE QUALQUER DESTES SEJA CONTROLADOR, SÓCIO, ACIONISTA OU PARTICIPANTE, DE FORMA DIRETA OU INDIRETA, RECEBIMENTO POR PALESTRAS, AULAS, ATUAÇÃO COMO PROCTOR DE TREINAMENTOS, REMUNERAÇÕES, HONORÁRIOS PAGOS POR PARTICIPAÇÕES EM CONSELHOS CONSULTIVOS, DE INVESTIGADORES, OU OUTROS COMITÊS, ETC. PROVENIENTES DA INDÚSTRIA FARMACÊUTICA, DE ÓRTESES, PRÓTESES, EQUIPAMENTOS E IMPLANTES, BRASILEIRAS OU ESTRANGEIRAS: - Sanofi: Xarelto - Novartis: Alpelisib OUTROS RELACIONAMENTOS FINANCIAMENTO DE ATIVIDADES DE EDUCAÇÃO MÉDICA CONTINUADA, INCLUINDO VIAGENS, HOSPEDAGENS E INSCRIÇÕES PARA CONGRESSOS E CURSOS, PROVENIENTES DA INDÚSTRIA FARMACÊUTICA, DE ÓRTESES, PRÓTESES, EQUIPAMENTOS E IMPLANTES, BRASILEIRAS OU ESTRANGEIRAS: - Janssen: Abiraterona - Bayer: Xofigo - Libbs: Zedora
Fernando Meton de Alencar Camara Vieira	DECLARAÇÃO FINANCEIRA A - PAGAMENTO DE QUALQUER ESPÉCIE E DESDE QUE ECONOMICAMENTE APRECIÁVEIS, FEITOS A (i) VOCÊ, (ii) AO SEU CÔNJUGE/COMPANHEIRO OU A QUALQUER OUTRO MEMBRO QUE RESIDA COM VOCÊ, (iii) A QUALQUER PESSOA JURÍDICA EM QUE QUALQUER DESTES SEJA CONTROLADOR, SÓCIO, ACIONISTA OU PARTICIPANTE, DE FORMA DIRETA OU INDIRETA, RECEBIMENTO POR PALESTRAS, AULAS, ATUAÇÃO COMO PROCTOR DE TREINAMENTOS, REMUNERAÇÕES, HONORÁRIOS PAGOS POR PARTICIPAÇÕES EM CONSELHOS CONSULTIVOS, DE INVESTIGADORES, OU OUTROS COMITÊS, ETC. PROVENIENTES DA INDÚSTRIA FARMACÊUTICA, DE ÓRTESES, PRÓTESES, EQUIPAMENTOS E IMPLANTES, BRASILEIRAS OU ESTRANGEIRAS: - BMS: câncer colorretal (pesquisa clínica)
Gustavo dos Santos Fernandes	DECLARAÇÃO FINANCEIRA A - PAGAMENTO DE QUALQUER ESPÉCIE E DESDE QUE ECONOMICAMENTE APRECIÁVEIS, FEITOS A (i) VOCÊ, (ii) AO SEU CÔNJUGE/COMPANHEIRO OU A QUALQUER OUTRO MEMBRO QUE RESIDA COM VOCÊ, (iii) A QUALQUER PESSOA JURÍDICA EM QUE QUALQUER DESTES SEJA CONTROLADOR, SÓCIO, ACIONISTA OU PARTICIPANTE, DE FORMA DIRETA OU INDIRETA, RECEBIMENTO POR PALESTRAS, AULAS, ATUAÇÃO COMO PROCTOR DE TREINAMENTOS, REMUNERAÇÕES, HONORÁRIOS PAGOS POR PARTICIPAÇÕES EM CONSELHOS CONSULTIVOS, DE INVESTIGADORES, OU OUTROS COMITÊS, ETC. PROVENIENTES DA INDÚSTRIA FARMACÊUTICA, DE ÓRTESES, PRÓTESES, EQUIPAMENTOS E IMPLANTES, BRASILEIRAS OU ESTRANGEIRAS: - Roche: Bevacizumab e Trastuzumab - MSD: imunoterapia - BMS: imunoterapia - Bayer - Sanofi - Novartis B - FINANCIAMENTO DE PESQUISAS SOB SUA RESPONSABILIDADE DIRETA/PESSOAL (DIRECIONADO AO DEPARTAMENTO OU INSTITUIÇÃO) PROVENIENTES DA INDÚSTRIA FARMACÊUTICA, DE ÓRTESES, PRÓTESES, EQUIPAMENTOS E IMPLANTES, BRASILEIRAS OU ESTRANGEIRAS: - BMS: imunoterapia - MSD: imunoterapia - Roche: imunoterapia
Helano Freitas	Nada a ser declarado
Ibraim Masciarelli F. Pinto	DECLARAÇÃO FINANCEIRA A - PAGAMENTO DE QUALQUER ESPÉCIE E DESDE QUE ECONOMICAMENTE APRECIÁVEIS, FEITOS A (i) VOCÊ, (ii) AO SEU CÔNJUGE/COMPANHEIRO OU A QUALQUER OUTRO MEMBRO QUE RESIDA COM VOCÊ, (iii) A QUALQUER PESSOA JURÍDICA EM QUE QUALQUER DESTES SEJA CONTROLADOR, SÓCIO, ACIONISTA OU PARTICIPANTE, DE FORMA DIRETA OU INDIRETA, RECEBIMENTO POR PALESTRAS, AULAS, ATUAÇÃO COMO PROCTOR DE TREINAMENTOS, REMUNERAÇÕES, HONORÁRIOS PAGOS POR PARTICIPAÇÕES EM CONSELHOS CONSULTIVOS, DE INVESTIGADORES, OU OUTROS COMITÊS, ETC. PROVENIENTES DA INDÚSTRIA FARMACÊUTICA, DE ÓRTESES, PRÓTESES, EQUIPAMENTOS E IMPLANTES, BRASILEIRAS OU ESTRANGEIRAS: - GE Healthcare: tomografia - Novo Nordisk: farma
Isabela Bispo Santos da Silva da Costa	Nada a ser declarado
João Cesar Nunes Sbano	Nada a ser declarado
José Antônio Franchini Ramires	Nada a ser declarado
Júlia Tizue Fukushima	Nada a ser declarado
Juliana Barbosa Sobral Alves	DECLARAÇÃO FINANCEIRA A - PAGAMENTO DE QUALQUER ESPÉCIE E DESDE QUE ECONOMICAMENTE APRECIÁVEIS, FEITOS A (i) VOCÊ, (ii) AO SEU CÔNJUGE/COMPANHEIRO OU A QUALQUER OUTRO MEMBRO QUE RESIDA COM VOCÊ, (iii) A QUALQUER PESSOA JURÍDICA EM QUE QUALQUER DESTES SEJA CONTROLADOR, SÓCIO, ACIONISTA OU PARTICIPANTE, DE FORMA DIRETA OU INDIRETA, RECEBIMENTO POR PALESTRAS, AULAS, ATUAÇÃO COMO PROCTOR DE TREINAMENTOS, REMUNERAÇÕES, HONORÁRIOS PAGOS POR PARTICIPAÇÕES EM CONSELHOS CONSULTIVOS, DE INVESTIGADORES, OU OUTROS COMITÊS, ETC. PROVENIENTES DA INDÚSTRIA FARMACÊUTICA, DE ÓRTESES, PRÓTESES, EQUIPAMENTOS E IMPLANTES, BRASILEIRAS OU ESTRANGEIRAS: - Janssen: hipertensão pulmonar - Bayer: hipertensão
Juliana Pereira	Nada a ser declarado
Laura Testa	DECLARAÇÃO FINANCEIRA A - PAGAMENTO DE QUALQUER ESPÉCIE E DESDE QUE ECONOMICAMENTE APRECIÁVEIS, FEITOS A (i) VOCÊ, (ii) AO SEU CÔNJUGE/COMPANHEIRO OU A QUALQUER OUTRO MEMBRO QUE RESIDA COM VOCÊ, (iii) A QUALQUER PESSOA JURÍDICA EM QUE QUALQUER DESTES SEJA CONTROLADOR, SÓCIO, ACIONISTA OU PARTICIPANTE, DE FORMA DIRETA OU INDIRETA, RECEBIMENTO POR PALESTRAS, AULAS, ATUAÇÃO COMO PROCTOR DE TREINAMENTOS, REMUNERAÇÕES, HONORÁRIOS PAGOS POR PARTICIPAÇÕES EM CONSELHOS CONSULTIVOS, DE INVESTIGADORES, OU OUTROS COMITÊS, ETC. PROVENIENTES DA INDÚSTRIA FARMACÊUTICA, DE ÓRTESES, PRÓTESES, EQUIPAMENTOS E IMPLANTES, BRASILEIRAS OU ESTRANGEIRAS: - Libbs: oncologia - Novartis: oncologia - Roche: oncologia - Pfizer: oncologia B - FINANCIAMENTO DE PESQUISAS SOB SUA RESPONSABILIDADE DIRETA/PESSOAL (DIRECIONADO AO DEPARTAMENTO OU INSTITUIÇÃO) PROVENIENTES DA INDÚSTRIA FARMACÊUTICA, DE ÓRTESES, PRÓTESES, EQUIPAMENTOS E IMPLANTES, BRASILEIRAS OU ESTRANGEIRAS: - Roche: oncologia - financiamento institucional - Lilly: oncologia - financiamento institucional - Novartis: oncologia - financiamento institucional - MSD: oncologia - financiamento institucional OUTROS RELACIONAMENTOS FINANCIAMENTO DE ATIVIDADES DE EDUCAÇÃO MÉDICA CONTINUADA, INCLUINDO VIAGENS, HOSPEDAGENS E INSCRIÇÕES PARA CONGRESSOS E CURSOS, PROVENIENTES DA INDÚSTRIA FARMACÊUTICA, DE ÓRTESES, PRÓTESES, EQUIPAMENTOS E IMPLANTES, BRASILEIRAS OU ESTRANGEIRAS: - Pfizer: oncologia - Libbs: oncologia - United Medical: oncologia
Ludhmila Abrahão Hajjar	Nada a ser declarado
Luís Beck-da-Silva	DECLARAÇÃO FINANCEIRA A - PAGAMENTO DE QUALQUER ESPÉCIE E DESDE QUE ECONOMICAMENTE APRECIÁVEIS, FEITOS A (i) VOCÊ, (ii) AO SEU CÔNJUGE/COMPANHEIRO OU A QUALQUER OUTRO MEMBRO QUE RESIDA COM VOCÊ, (iii) A QUALQUER PESSOA JURÍDICA EM QUE QUALQUER DESTES SEJA CONTROLADOR, SÓCIO, ACIONISTA OU PARTICIPANTE, DE FORMA DIRETA OU INDIRETA, RECEBIMENTO POR PALESTRAS, AULAS, ATUAÇÃO COMO PROCTOR DE TREINAMENTOS, REMUNERAÇÕES, HONORÁRIOS PAGOS POR PARTICIPAÇÕES EM CONSELHOS CONSULTIVOS, DE INVESTIGADORES, OU OUTROS COMITÊS, ETC. PROVENIENTES DA INDÚSTRIA FARMACÊUTICA, DE ÓRTESES, PRÓTESES, EQUIPAMENTOS E IMPLANTES, BRASILEIRAS OU ESTRANGEIRAS: - Novartis: insuficiência cardíaca - Merck: insuficiência cardíaca B - FINANCIAMENTO DE PESQUISAS SOB SUA RESPONSABILIDADE DIRETA/PESSOAL (DIRECIONADO AO DEPARTAMENTO OU INSTITUIÇÃO) PROVENIENTES DA INDÚSTRIA FARMACÊUTICA, DE ÓRTESES, PRÓTESES, EQUIPAMENTOS E IMPLANTES, BRASILEIRAS OU ESTRANGEIRAS: - AMGEN: insuficiência cardíaca - Novartis: insuficiência cardíaca
Manuel Maria Ramos Valente Neto	Nada a ser declarado
Marcelo Antônio Cartaxo Queiroga Lopes	Nada a ser declarado
Marcelo Westerlund Montera	Nada a ser declarado
Marcus Vinicius Bolivar Malachias	DECLARAÇÃO FINANCEIRA A - PAGAMENTO DE QUALQUER ESPÉCIE E DESDE QUE ECONOMICAMENTE APRECIÁVEIS, FEITOS A (i) VOCÊ, (ii) AO SEU CÔNJUGE/COMPANHEIRO OU A QUALQUER OUTRO MEMBRO QUE RESIDA COM VOCÊ, (iii) A QUALQUER PESSOA JURÍDICA EM QUE QUALQUER DESTES SEJA CONTROLADOR, SÓCIO, ACIONISTA OU PARTICIPANTE, DE FORMA DIRETA OU INDIRETA, RECEBIMENTO POR PALESTRAS, AULAS, ATUAÇÃO COMO PROCTOR DE TREINAMENTOS, REMUNERAÇÕES, HONORÁRIOS PAGOS POR PARTICIPAÇÕES EM CONSELHOS CONSULTIVOS, DE INVESTIGADORES, OU OUTROS COMITÊS, ETC. PROVENIENTES DA INDÚSTRIA FARMACÊUTICA, DE ÓRTESES, PRÓTESES, EQUIPAMENTOS E IMPLANTES, BRASILEIRAS OU ESTRANGEIRAS: - Abbott: cardiologia - Libbs: cardiologia - Bayewr: cardiologia - Novo Nordisk: cardiologia OUTROS RELACIONAMENTOS FINANCIAMENTO DE ATIVIDADES DE EDUCAÇÃO MÉDICA CONTINUADA, INCLUINDO VIAGENS, HOSPEDAGENS E INSCRIÇÕES PARA CONGRESSOS E CURSOS, PROVENIENTES DA INDÚSTRIA FARMACÊUTICA, DE ÓRTESES, PRÓTESES, EQUIPAMENTOS E IMPLANTES, BRASILEIRAS OU ESTRANGEIRAS: - AstraZeneca - Bayer PARTICIPAÇÃO SOCIETÁRIA DE QUALQUER NATUREZA E QUALQUER VALOR ECONOMICAMENTE APRECIÁVEL DE EMPRESAS NA ÁREA DE SAÚDE, DE ENSINO OU EM EMPRESAS CONCORRENTES OU FORNECEDORAS DA SBC: - Instituto de Hipertensão de Minas Gerais, Cardio Check Up
Maria Carolina Feres de Almeida Soeiro	Nada a ser declarado
Maria da Consolação Vieira Moreira	Nada a ser declarado
Maria Del Pilar Estevez Diz	Nada a ser declarado
Maria Verônica Câmara dos Santos	Nada a ser declarado
Marianna Deway Andrade Dracoulakis	DECLARAÇÃO FINANCEIRA A - PAGAMENTO DE QUALQUER ESPÉCIE E DESDE QUE ECONOMICAMENTE APRECIÁVEIS, FEITOS A (i) VOCÊ, (ii) AO SEU CÔNJUGE/COMPANHEIRO OU A QUALQUER OUTRO MEMBRO QUE RESIDA COM VOCÊ, (iii) A QUALQUER PESSOA JURÍDICA EM QUE QUALQUER DESTES SEJA CONTROLADOR, SÓCIO, ACIONISTA OU PARTICIPANTE, DE FORMA DIRETA OU INDIRETA, RECEBIMENTO POR PALESTRAS, AULAS, ATUAÇÃO COMO PROCTOR DE TREINAMENTOS, REMUNERAÇÕES, HONORÁRIOS PAGOS POR PARTICIPAÇÕES EM CONSELHOS CONSULTIVOS, DE INVESTIGADORES, OU OUTROS COMITÊS, ETC. PROVENIENTES DA INDÚSTRIA FARMACÊUTICA, DE ÓRTESES, PRÓTESES, EQUIPAMENTOS E IMPLANTES, BRASILEIRAS OU ESTRANGEIRAS: - Pfizer: cardiologia - Bayer: Xarelto - Daichii: Lixiana - Servier: cardiologia OUTROS RELACIONAMENTOS FINANCIAMENTO DE ATIVIDADES DE EDUCAÇÃO MÉDICA CONTINUADA, INCLUINDO VIAGENS, HOSPEDAGENS E INSCRIÇÕES PARA CONGRESSOS E CURSOS, PROVENIENTES DA INDÚSTRIA FARMACÊUTICA, DE ÓRTESES, PRÓTESES, EQUIPAMENTOS E IMPLANTES, BRASILEIRAS OU ESTRANGEIRAS: - Pfizer: Eliquis
Marília Harumi Higuchi dos Santos Rehder	DECLARAÇÃO FINANCEIRA A - PAGAMENTO DE QUALQUER ESPÉCIE E DESDE QUE ECONOMICAMENTE APRECIÁVEIS, FEITOS A (i) VOCÊ, (ii) AO SEU CÔNJUGE/COMPANHEIRO OU A QUALQUER OUTRO MEMBRO QUE RESIDA COM VOCÊ, (iii) A QUALQUER PESSOA JURÍDICA EM QUE QUALQUER DESTES SEJA CONTROLADOR, SÓCIO, ACIONISTA OU PARTICIPANTE, DE FORMA DIRETA OU INDIRETA, RECEBIMENTO POR PALESTRAS, AULAS, ATUAÇÃO COMO PROCTOR DE TREINAMENTOS, REMUNERAÇÕES, HONORÁRIOS PAGOS POR PARTICIPAÇÕES EM CONSELHOS CONSULTIVOS, DE INVESTIGADORES, OU OUTROS COMITÊS, ETC. PROVENIENTES DA INDÚSTRIA FARMACÊUTICA, DE ÓRTESES, PRÓTESES, EQUIPAMENTOS E IMPLANTES, BRASILEIRAS OU ESTRANGEIRAS: - Eli Lilly do Brasil OUTROS RELACIONAMENTOS VÍNCULO EMPREGATÍCIO COM A INDÚSTRIA FARMACÊUTICA, DE ÓRTESES, PRÓTESES, EQUIPAMENTOS E IMPLANTES, BRASILEIRAS OU ESTRANGEIRAS, ASSIM COMO SE TEM RELAÇÃO VÍNCULO EMPREGATÍCIO COM OPERADORAS DE PLANOS DE SAÚDE OU EM AUDITORIAS MÉDICAS (INCLUINDO MEIO PERÍODO) DURANTE O ANO PARA O QUAL VOCÊ ESTÁ DECLARANDO: - Gerente médico farmacovigilância
Patricia Tavares Felipe Marcatti	Nada a ser declarado
Paulo Marcelo Gehm Hoff	DECLARAÇÃO FINANCEIRA A - PAGAMENTO DE QUALQUER ESPÉCIE E DESDE QUE ECONOMICAMENTE APRECIÁVEIS, FEITOS A (i) VOCÊ, (ii) AO SEU CÔNJUGE/COMPANHEIRO OU A QUALQUER OUTRO MEMBRO QUE RESIDA COM VOCÊ, (iii) A QUALQUER PESSOA JURÍDICA EM QUE QUALQUER DESTES SEJA CONTROLADOR, SÓCIO, ACIONISTA OU PARTICIPANTE, DE FORMA DIRETA OU INDIRETA, RECEBIMENTO POR PALESTRAS, AULAS, ATUAÇÃO COMO PROCTOR DE TREINAMENTOS, REMUNERAÇÕES, HONORÁRIOS PAGOS POR PARTICIPAÇÕES EM CONSELHOS CONSULTIVOS, DE INVESTIGADORES, OU OUTROS COMITÊS, ETC. PROVENIENTES DA INDÚSTRIA FARMACÊUTICA, DE ÓRTESES, PRÓTESES, EQUIPAMENTOS E IMPLANTES, BRASILEIRAS OU ESTRANGEIRAS: - Exelixis: oncologia - Bayer: oncologia - Lilly: oncologia - United B - FINANCIAMENTO DE PESQUISAS SOB SUA RESPONSABILIDADE DIRETA/PESSOAL (DIRECIONADO AO DEPARTAMENTO OU INSTITUIÇÃO) PROVENIENTES DA INDÚSTRIA FARMACÊUTICA, DE ÓRTESES, PRÓTESES, EQUIPAMENTOS E IMPLANTES, BRASILEIRAS OU ESTRANGEIRAS: - Exelixis: oncologia - Bayer: oncologia - AstraZeneca: oncologia - United - Lilly - Pfizer - Sanofi - Roche - BMS - MSD - Merck - Novartis OUTROS RELACIONAMENTOS PARTICIPAÇÃO EM COMITÊS DE COMPRAS DE MATERIAIS OU FÁRMACOS EM INSTITUIÇÕES DE SAÚDE OU FUNÇÕES ASSEMELHADAS: - Comitê de farmácia - ICESP
Renata do Val	Nada a ser declarado
Ricardo Pavanello	OUTROS RELACIONAMENTOS FINANCIAMENTO DE ATIVIDADES DE EDUCAÇÃO MÉDICA CONTINUADA, INCLUINDO VIAGENS, HOSPEDAGENS E INSCRIÇÕES PARA CONGRESSOS E CURSOS, PROVENIENTES DA INDÚSTRIA FARMACÊUTICA, DE ÓRTESES, PRÓTESES, EQUIPAMENTOS E IMPLANTES, BRASILEIRAS OU ESTRANGEIRAS: - Bayer: Programa de Educação Continuada a Distância
Roberto Kalil Filho	Nada a ser declarado
Sílvia Marinho Martins Alves	Nada a ser declarado
Silvia Moreira Ayub Ferreira	DECLARAÇÃO FINANCEIRA A - PAGAMENTO DE QUALQUER ESPÉCIE E DESDE QUE ECONOMICAMENTE APRECIÁVEIS, FEITOS A (i) VOCÊ, (ii) AO SEU CÔNJUGE/COMPANHEIRO OU A QUALQUER OUTRO MEMBRO QUE RESIDA COM VOCÊ, (iii) A QUALQUER PESSOA JURÍDICA EM QUE QUALQUER DESTES SEJA CONTROLADOR, SÓCIO, ACIONISTA OU PARTICIPANTE, DE FORMA DIRETA OU INDIRETA, RECEBIMENTO POR PALESTRAS, AULAS, ATUAÇÃO COMO PROCTOR DE TREINAMENTOS, REMUNERAÇÕES, HONORÁRIOS PAGOS POR PARTICIPAÇÕES EM CONSELHOS CONSULTIVOS, DE INVESTIGADORES, OU OUTROS COMITÊS, ETC. PROVENIENTES DA INDÚSTRIA FARMACÊUTICA, DE ÓRTESES, PRÓTESES, EQUIPAMENTOS E IMPLANTES, BRASILEIRAS OU ESTRANGEIRAS: - Abbott: MitraClip OUTROS RELACIONAMENTOS FINANCIAMENTO DE ATIVIDADES DE EDUCAÇÃO MÉDICA CONTINUADA, INCLUINDO VIAGENS, HOSPEDAGENS E INSCRIÇÕES PARA CONGRESSOS E CURSOS, PROVENIENTES DA INDÚSTRIA FARMACÊUTICA, DE ÓRTESES, PRÓTESES, EQUIPAMENTOS E IMPLANTES, BRASILEIRAS OU ESTRANGEIRAS: - Abbott: assistência circulatória mecânica
Silvia Moulin Ribeiro Fonseca	Nada a ser declarado
Stephanie Itala Rizk	Nada a ser declarado
Thiago Liguori Feliciano da Silva	Nada a ser declarado
Vanderson Rocha	Nada a ser declarado
Veronica Cristina Quiroga Fonseca	Nada a ser declarado
Wilson Mathias Junior	Nada a ser declarado
Yana Novis	Nada a ser declarado

## 1. Introdução

Atualmente, doenças cardiovasculares (DCV) e câncer são as principais causas de mortalidade em todo o mundo e no Brasil.^1^^–^^3^ As transições demográfica e epidemiológica ocorridas recentemente no nosso país resultaram no aumento da expectativa de vida da população, hoje em torno de 76 anos, e na modificação no perfil de saúde, em que doenças crônicas e suas complicações são prevalentes.^4^

Esses fatores ocasionam importantes desafios e a necessidade de uma agenda para as políticas de saúde que possam lidar com as várias transições em curso. A escalada tecnológica, a escassez de análises de custo-efetividade e a pouca valorização na educação quanto aos aspectos referentes ao acesso à saúde e à promoção e prevenção em saúde impõem a necessidade da implementação de diretrizes e consensos para auxiliar na utilização de protocolos sistematizados, com o objetivo de adequar a prática clínica independentemente da localização geográfica da instituição de saúde e da heterogeneidade de recursos.

Avanços recentes na detecção e no tratamento do câncer tiveram como consequência o aumento exponencial no número de sobreviventes em todo o mundo. Em recente projeção, estima-se para 2026, nos Estados Unidos, uma população de 20 milhões de sobreviventes de câncer, 50% dos quais terão mais de 70 anos de idade.^5^^,^^6^ Uma população mais idosa com história de câncer e de DCV associada ao potencial de toxicidade cardiovascular do tratamento oncológico impõe a necessidade de especialistas com conhecimento a respeito da interação câncer e DCV.^7^

Em 1967, foi feita a primeira descrição de toxicidade cardíaca associada a antraciclina.^8^ Em 1971, descreveu-se que a cardiotoxicidade por antraciclina seria dose-dependente e o dano cardíaco possivelmente irreversível.^9^ Alguns anos depois, foram identificados fatores de risco para disfunção ventricular associada à quimioterapia, tendo-se relacionado biomarcadores, como troponina e BNP, à predição de eventos cardiovasculares.^10^^,^^11^ Esses foram os primeiros achados que nortearam a cardio-oncologia.

**Figura 1:**
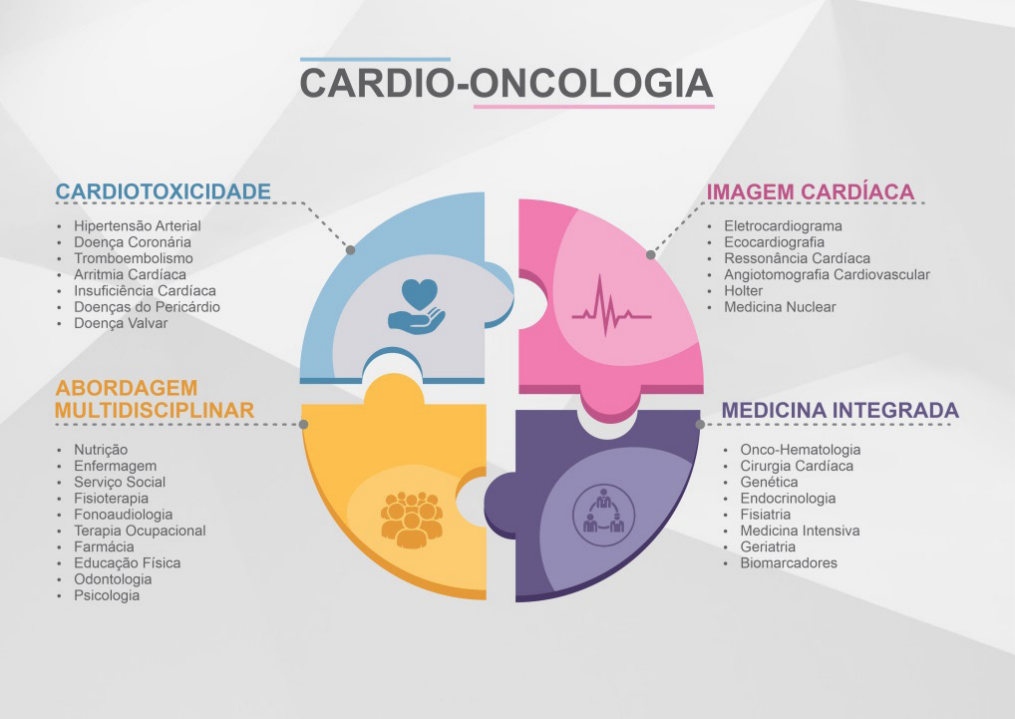
As fronteiras da cardio-oncologia da atualidade.

Cardio-oncologia é o campo da ciência voltado para o diagnóstico precoce e o manejo adequado da DCV em pacientes com diagnóstico atual ou pregresso de câncer. A cardio-oncologia ocupa-se ainda da análise do risco cardiovascular frente ao diagnóstico oncológico, além das necessidades do paciente antes, durante e após o tratamento. O time de especialistas em cardio-oncologia deve seguir o paciente desde o diagnóstico, passando por todas as fases do tratamento, e acompanhá-lo mesmo após sua cura, quando é denominado sobrevivente do câncer. A necessidade crescente da expansão da cardio-oncologia guarda relação direta com a epidemiologia do câncer e das DCV, os seus fatores de risco em comum e a multiplicidade de tratamentos com distintas toxicidades ao sistema cardiovascular (Figura 1).^12^^,^^13^

Em 2011, a Sociedade Brasileira de Cardiologia (SBC) e a Sociedade Brasileira de Oncologia Clínica (SBOC) foram pioneiras ao publicarem em conjunto a I Diretriz de Cardio-Oncologia.^14^ Em 9 anos, pudemos observar o crescimento significativo da disciplina de cardio-oncologia. Isso ocorreu devido a alguns fatores: a) avanços consideráveis no tratamento do câncer; b) entendimento da multidisciplinaridade e da integração entre cardiologia, oncologia e hematologia como essenciais ao cuidado do paciente com câncer; c) implementação de programas de *fellowship* em todo o mundo e inserção da cardio-oncologia na matriz curricular da residência em cardiologia em algumas instituições; d) crescimento da pesquisa na área tanto básica quanto clínica; e e) criação de importantes periódicos dedicados ao tema, como *JACC CardioOncology* e *Cardio-Oncology*.^15^^,^^16^

Destaca-se o fato de, em 2019, o Brasil ter sido a sede do *V Global Cardio-Oncology Summit*, que contou com a participação de especialistas de diversos países e de aproximadamente 600 profissionais (cardiologistas, oncologistas, hematologistas, enfermeiros, fisioterapeutas, farmacêuticos, educadores físicos). Foram publicados 89 resumos no jornal *Frontiers in Cardiovascular Medicine*, tendo o periódico *JACC CardioOncology* publicado *“Proceedings From the Global Cardio-Oncology Summit - The Top 10 Priorities to Actualize for CardioOncology*”.^17^^,^^18^

A SBC e a SBOC, com o objetivo de renovar o conhecimento e promover a implementação da abordagem racional e sistemática das complicações cardiovasculares no paciente oncológico, reuniram um grupo de especialistas para abordar novas estratégias, propor recomendações baseadas em evidências e desenvolver o cuidado multidisciplinar, que permitirão o manejo adequado dessa categoria crescente de pacientes.

A *Diretriz Brasileira de Cardio-Oncologia - 2020* tem como metas: 1) desmistificar a visão da DCV como uma barreira ao tratamento efetivo do paciente com câncer; 2) prevenir e reduzir os riscos da cardiotoxicidade do tratamento; 3) promover a interação das especialidades (cardiologia, hematologia e oncologia) para obter a melhor estratégia de cuidado para o paciente, considerando os riscos e os benefícios do tratamento; 4) propor a unificação de terminologias e definições das complicações cardiovasculares no paciente com câncer, com o objetivo de homogeneizar a assistência e a pesquisa; 5) divulgar as evidências disponíveis em relação ao manejo das complicações cardiovasculares no paciente oncológico, buscando diagnóstico precoce por meio da monitorização da função cardiovascular antes, durante e após o tratamento do paciente; 6) promover tratamento adequado em conjunto com a oncologia e a hematologia com base em evidências científicas, em análise de risco e na personalização do cuidado, levando-se em consideração as preferências do paciente; e 7) estimular a pesquisa e a disseminação do conhecimento na área de cardio-oncologia.

A *Diretriz Brasileira de Cardio-Oncologia - 2020* reúne a evidência disponível até 2020 no que se refere às complicações cardiovasculares dos pacientes com câncer.

## 2. Métodos

A *Diretriz Brasileira de Cardio-Oncologia – 2020* foi realizada de acordo com as recomendações vigentes. Um grupo de especialistas dos campos da cardiologia, oncologia e hematologia formou o comitê responsável pelo manuscrito. Os participantes foram escolhidos por seu destaque na área, sua participação na *International Cardio-Oncology Society* (ICOS), na SBC e na SBOC, além de sua produção científica.

Foi realizada pesquisa bibliográfica no PubMed no período de 1975 a julho de 2020 com as seguintes palavras-chave: *cardiotoxicity, cancer, immunotherapy, cardiooncology, cardiovascular complications, targeted therapy, radiotherapy, vascular toxicity, heart failure, ventricular dysfunction, pericardial disease, coronary disease, thromboembolism, arrhythmias, hypertension, individual drug names*. O manuscrito foi enviado eletronicamente para todos os participantes e, após a concordância de todos com o conteúdo, foi formatado e encaminhado para publicação.

Seguem as classes de recomendação e níveis de evidência utilizados por esta diretriz.

Classes (graus) de recomendação:

Classe I – Condições para as quais há evidências conclusivas, ou, na sua falta, consenso geral de que o procedimento é seguro e útil/eficaz.

Classe II – Condições para as quais há evidências conflitantes e/ou divergência de opinião sobre segurança e utilidade/eficácia do procedimento.

- Classe IIa – Peso ou evidência/opinião a favor do procedimento. A maioria aprova.
- Classe IIb – Segurança e utilidade/eficácia menos bem estabelecida, não havendo predomínio de opiniões a favor.

Classe III – Condições para as quais há evidências e/ou consenso de que o procedimento não é útil/eficaz e, em alguns casos, pode ser prejudicial Níveis de evidência.

Níveis de evidência:

Nível A – Dados obtidos a partir de múltiplos estudos randomizados de bom porte, concordantes e/ou de metanálise robusta de estudos clínicos randomizados.

Nível B – Dados obtidos a partir de metanálise menos robusta, a partir de um único estudo randomizado ou de estudos não randomizados (observacionais).

Nível C – Dados obtidos de opiniões consensuais de especialistas.

## 3. Diagnóstico e Manejo de Complicações Cardiovasculares no Paciente com Câncer

### 3.1. Avaliação Cardiológica Inicial do Paciente

O tratamento do câncer, em suas diversas modalidades (quimioterapia, imunoterapia, radioterapia, dentre outras), pode resultar em dano ao sistema cardiovascular. Pacientes com DCV prévia ou fatores de risco cardiovasculares representam o grupo com maior possibilidade para complicações decorrentes do tratamento. Portanto, recomenda-se o tratamento e o controle dos fatores de risco cardiovasculares na população de pacientes com câncer.^19^^–^^21^

A consulta com o cardiologista deve abordar controle dos fatores de risco cardiovasculares, medidas de cardioproteção, adesão ao tratamento e estratégia para permitir o diagnóstico precoce de dano cardíaco (I, B).

**Figura 2:**
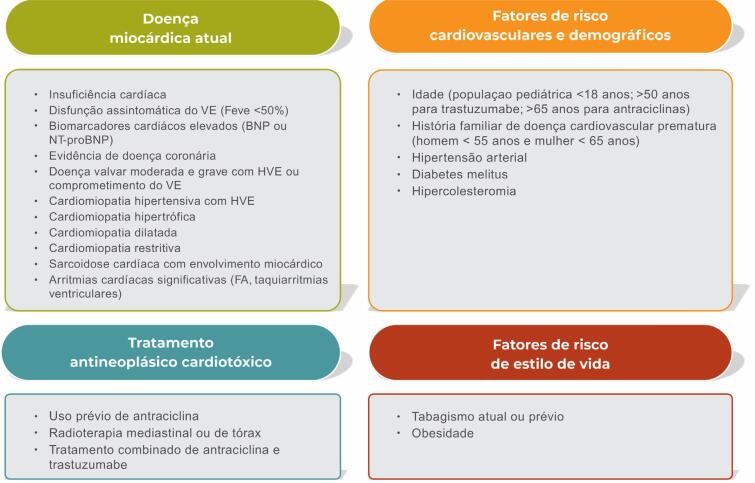
Fatores de risco presentes no paciente com câncer que predispõem a cardiotoxicidade. Adaptado de Zamorano et al.22 BNP: peptídeo natriurético tipo B; FEVE: fração de ejeção do ventrículo esquerdo; HVE: hipertrofia ventricular esquerda; FA: fibrilação atrial; NT-proBNP: fragmento N-terminal do peptídeo natriurético tipo B; VE: ventrículo esquerdo.

Pacientes com fatores de risco cardiovasculares ou DCV já estabelecida e que serão submetidos a tratamento com potencial de cardiotoxicidade [antraciclinas, agentes anti-HER2 (receptor tipo 2 do fator de crescimento epidérmico humano), agentes alquilantes, inibidores de sinalização VEGF (fator de crescimento endotelial vascular), inibidores de proteassoma e inibidores de *checkpoint* imunológicos (ICIs)] devem ser avaliados pelo cardiologista no início da terapia e acompanhados de acordo com protocolos de seguimento específicos (I,B). A Tabela 1 apresenta os tratamentos antineoplásicos mais comumente associados a toxicidade cardiovascular e a Figura 2, os fatores associados a maior risco de cardiotoxicidade.

**Tabela 1:** Terapias antineoplásicas associadas à toxicidade cardiovascular

Classes de medicações antineoplásicas	Toxicidade cardiovascular
Radioterapia	Isquemia e infarto do miocárdio Doença pericárdica Doença valvar Miocardite Arritmia cardíaca
Antraciclinas (doxorrubicina, epirrubicina, daunorrubicina, idarrubicina, mitoxantrona)	Insuficiência cardíaca Disfunção ventricular assintomática Miocardite Pericardite Arritmias atriais e ventriculares
Agentes alquilantes (ciclofosfamida, ifosfamida, melfalan)	Arritmias Disfunção ventricular Doença arterial coronariana
Platina (cisplatina, carboplatina, oxaliplatina)	Trombose coronária Isquemia miocárdica Hipertensão arterial
Antimetabólitos (5-fluorouracil, capecitabina)	Isquemia miocárdica Vasoespasmo coronário Arritmias atriais e ventriculares
Terapias-alvo anti-HER2 (trastuzumabe, pertuzumabe, T-DM1, lapatinibe, neratinibe)	Insuficiência cardíaca Disfunção ventricular assintomática Hipertensão arterial
Inibidores de sinalização VEGF: Inibidores de tirosina quinase (sunitinibe, pazopanibe, sorafenibe, axitinibe, tivozanibe, cabozantinibe, regorafenibe, lenvatinibe, vandetinibe) Anticorpos monoclonais (bevacizumabe, ramucirumabe)	Hipertensão arterial Insuficiência cardíaca Disfunção ventricular assintomática Isquemia e infarto do miocárdio Prolongamento do QTc
Inibidores de tirosina quinase multi-alvo: Inibidores de tirosina quinase de segunda e terceira geração BCR-ABL (ponatinibe, nilotinibe, dasatinibe, bosutinibe)	Trombose arterial (infarto do miocárdio, acidente vascular cerebral e doença vascular periférica oclusiva*) Tromboembolismo venoso Hipertensão arterial Insuficiência cardíaca Disfunção ventricular assintomática Aterosclerose** Prolongamento do QTc** Hipertensão pulmonar***
Outros inibidores de tirosina quinase multi-alvo: Inibidores de ALK (crizotinibe, ceritinibe) Inibidores de PI3-AKT-mTor (everolimus, sirolimus) Inibidores de tirosina quinase de Bruton (ibrutinibe) Inibidor de tirosina quinase EGFR (osimertinibe)	Bradicardia, prolongamento do QTc Hiperglicemia, dislipidemia Fibrilação atrial Insuficiência cardíaca, fibrilação atrial, prolongamento do QTc Fibrilação atrial, insuficiência cardíaca
Terapia do mieloma múltiplo: Inibidores de proteassoma (carfilzomibe, bortezomibe, ixazomibe) Imunomoduladores (lenalidomide, talidomida, pomalidomide)	Insuficiência cardíaca**** Disfunção ventricular assintomática**** Isquemia e infarto do miocárdio Arritmias atriais e ventriculares Tromboembolismo venoso Trombose arterial Hipertensão arterial
Inibidores BRAF e MEK: (dabrafenibe + trametinibe, vemurafenibe + cobimetinibe, encorafenibe + binimetinibe)	Insuficiência cardíaca Disfunção ventricular assintomática Hipertensão arterial Prolongamento QTc*****
Terapias antiandrogênicas: Agonistas GnRH (goserelina, leuprolide) Antagonistas GnRH (degarelix) Antiandrogênicos (abiraterone)	Aterosclerose Isquemia e infarto do miocárdio Diabetes mellitus Hipertensão arterial
Inibidores de *checkpoint* imunológicos: (nivolumabe, ipilimumabe, durvalumabe, pembrolizumabe, atezolizumabe, avelumabe)	Miocardite Insuficiência cardíaca Arritmias atriais e ventriculares Isquemia miocárdica

* *Associado com ponatinibe*

** *Associado com ponatinibe e nilotinibe*

*** *Associado com dasatinibe*

**** *Associado com carfilzomibe*

***** *Associado com vemurafenibe e cobimetinibe. EGFR: receptor do fator de crescimento epidérmico; GnRH: hormônio liberador de gonadotrofina; HER2: receptor tipo 2 do fator de crescimento epidérmico humano; QTc: QT corrigido; T-DM1: ado-trastuzumabe entansina; VEGF: fator de crescimento endotelial vascular.*

A equipe multidisciplinar, ao avaliar o paciente, deve analisar os riscos e os supostos benefícios da terapia e implementar estratégias de prevenção de dano cardiovascular (IIa, C).

Mensuração de fatores de risco cardiovasculares e sua abordagem de acordo com consensos e diretrizes são recomendados (I, A).

**Figura 3:**
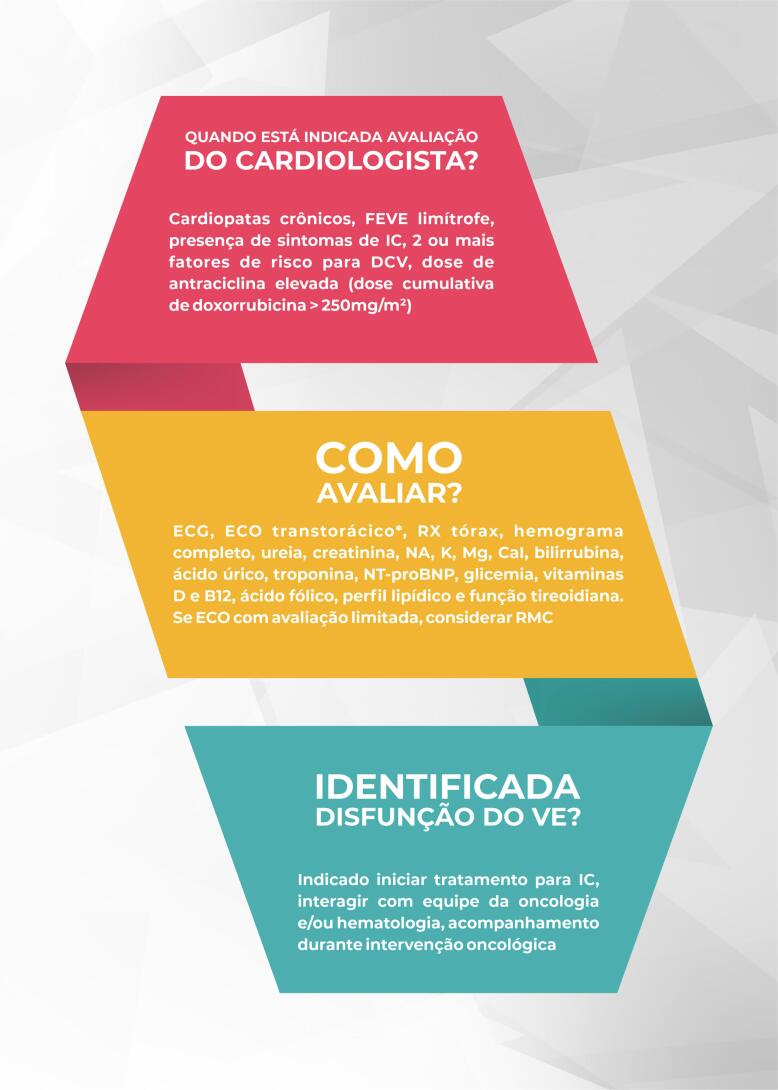
Avaliação inicial do cardiologista. *Idealmente com avaliação tridimensional da FEVE e strain miocárdico pela técnica speckle tracking. CaI: cálcio iônico sérico; DCV: doença cardiovascular; ECG: eletrocardiograma; ECO: ecocardiograma; FEVE: fração de ejeção do ventrículo esquerdo; IC: insuficiência cardíaca; K: potássio sérico; Mg: magnésio sérico; Na: sódio sérico; NT-proBNP: fragmento N-terminal do peptídeo natriurético tipo B; RMC: ressonância magnética cardíaca; RX: radiografia; VE: ventrículo esquerdo.

Na avaliação inicial do cardiologista, recomenda-se realizar anamnese, exame físico, eletrocardiograma (ECG), radiografia de tórax, hemograma completo, dosagem de eletrólitos e biomarcadores [fragmento N-terminal do BNP (NT-proBNP) e troponina I ou T ultrassensível], ácido fólico, vitaminas D e B12, além de verificar glicemia, perfil lipídico e funções renal, hepática e tireoidiana (I,A) (Figura 3).

Recomenda-se ainda realizar, na avaliação basal e seriada de acordo com o regime de tratamento, um ecocardiograma transtorácico com *Doppler* colorido, idealmente tridimensional, com análise da fração de ejeção do ventrículo esquerdo (FEVE), da função diastólica e da deformação miocárdica com mensuração do *strain* pela técnica *speckle tracking* (I, A).

Colaboração entre cardiologistas, oncologistas e hematologistas é recomendada para assegurar tratamento adequado e benéfico aos pacientes com câncer (IIa, A).

### 3.2. Diagnóstico de Cardiotoxicidade nos Pacientes com Câncer

O diagnóstico de cardiotoxicidade pode ser realizado pela confirmação de alteração cardiovascular nova durante ou após o tratamento, seja de natureza clínica e/ou alteração em biomarcadores e/ou em exame de imagem cardiovascular, tendo sido excluídas outras etiologias (I, B).

A ecocardiografia é o método de escolha para detectar disfunção miocárdica relacionada ao tratamento do câncer. A ecocardiografia tridimensional é o melhor método ecocardiográfico para medir a FEVE no paciente com câncer. Quando indisponível ou na presença de limitação, o método bidimensional de Simpson é recomendado (I, A).

Disfunção ventricular relacionada à terapia do câncer é definida como uma redução ≥ 10% na FEVE para um valor abaixo do limite inferior da normalidade (FEVE < 50%). Recomenda-se repetir a imagem cardiovascular em 2 a 3 semanas (I, B).

A redução da FEVE deve ocorrer durante a evolução, sendo classificada como sintomática ou assintomática e reversível ou irreversível (I, B).

O *strain longitudinal global* (SLG) é ferramenta que prediz com alta sensibilidade a posterior redução da FEVE. Redução ≥ 15% no SLG em relação ao basal é considerada anormal, sendo um marcador precoce de disfunção ventricular (I, B).

Recomenda-se realizar a análise da função diastólica nos pacientes oncológicos, tanto antes do início da terapia quanto no seguimento (IIa,C). Porém, não há evidência de que o tratamento deva ser interrompido com base na função diastólica.

A ventriculografia radioisotópica não é recomendada de rotina no paciente com câncer, em virtude da necessidade de radiação, devendo ser reservada para situações especiais, nas quais outras metodologias não estejam disponíveis (IIB, C).

A ressonância magnética cardíaca (RMC) é o método padrão-ouro para verificação da função cardíaca, capaz de avaliar estrutura e caracterização tecidual, sendo recomendada em casos com limitação da ecocardiografia, em situações de doenças infiltrativas, na avaliação do pericárdio e miocárdio e na detecção de massas e tumores (IIa, B). A RMC também pode ter valor prognóstico, por meio da análise de fibrose miocárdica.

O uso dos biomarcadores de rotina durante tratamento com potencial de cardiotoxicidade não está bem estabelecido. A monitorização da cardiotoxicidade por meio da dosagem de biomarcadores pode ser considerada para a detecção de lesão miocárdica precoce em pacientes de alto risco, devido a fatores prévios, ou expostos a fármacos como antraciclinas e trastuzumabe (IIa, B). Não se sabe ainda o melhor momento, em relação à quimioterapia, para a dosagem dos biomarcadores (durante a quimioterapia, 24h após, 48h após ou mais tardio) nem qual a conduta a ser tomada frente à detecção de níveis elevados. Além disso, sugere-se que sejam utilizados os mesmos *kits* de análise, por exemplo, de troponina ultrassensível e NT-proBNP durante a evolução do tratamento (IIa, C).

A presença de níveis elevados de biomarcadores (NT-proBNP e troponina) é indicativa de risco aumentado de cardiotoxicidade (I, A).

O ECG deve ser realizado na avaliação inicial e durante o tratamento. Deve-se calcular o QTc pela fórmula de Bazett QT / (RR)^1/2^ ou Fridericia QT / (RR)^1/3^ e utilizar o mesmo método durante a avaliação seriada do paciente. Nos pacientes com câncer, a fórmula de Fridericia é preferível, pois sofre menos alterações na presença de taquicardia ou bradicardia (IIa, C).

Na Tabela 2, estão descritos os métodos de diagnóstico cardiovascular, suas principais vantagens, aplicações e limitações.

**Tabela 2:** Métodos de diagnóstico cardiovascular, suas principais vantagens, aplicações e limitações

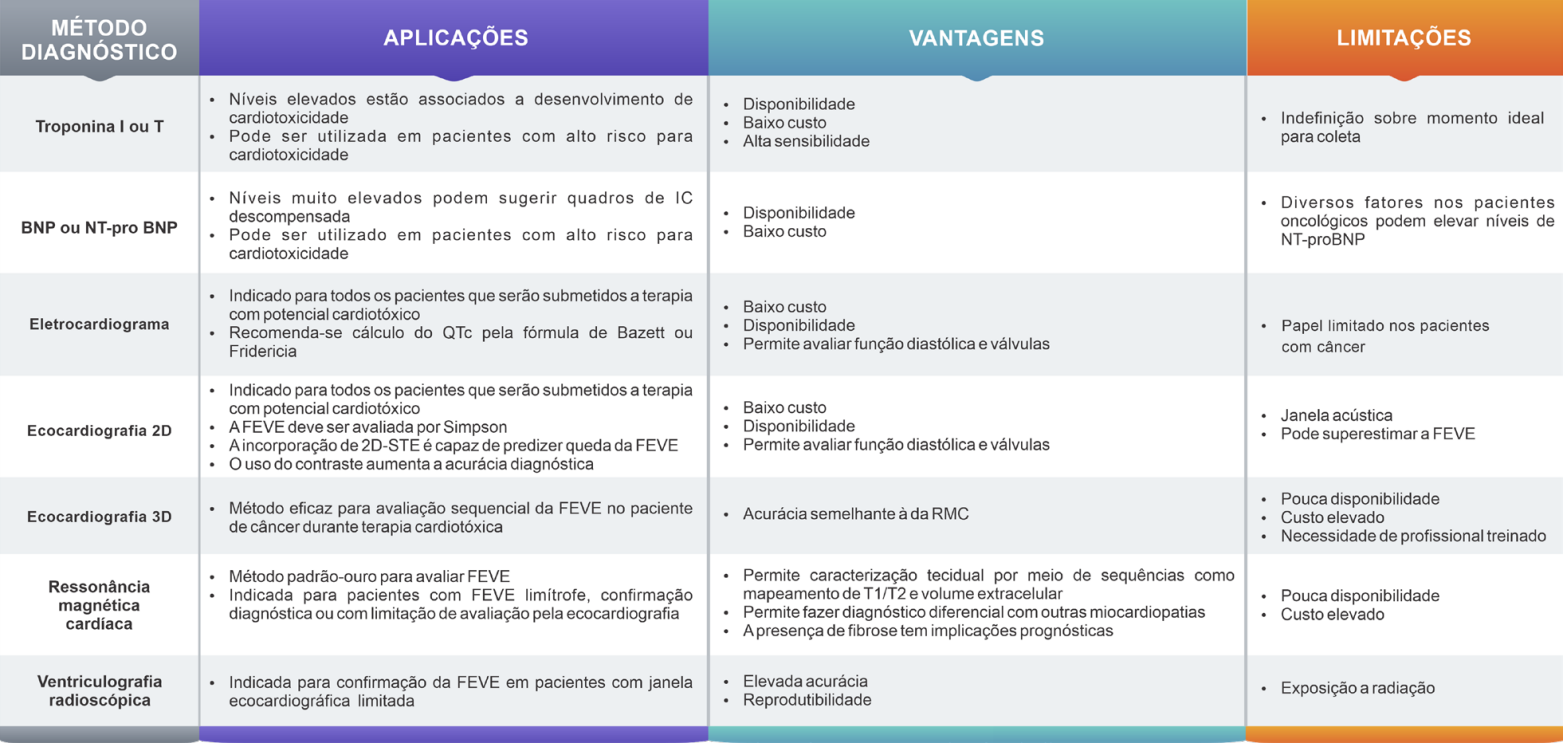

## 4. Disfunção Ventricular

A disfunção ventricular é uma das complicações mais graves do tratamento do câncer, caracterizando-se por altas taxas de morbidade e mortalidade. Pode surgir durante a terapia ou mesmo anos após seu término e ainda assim ser decorrente da toxicidade medicamentosa.^23^ O modelo clássico de disfunção ventricular como forma de cardiotoxicidade é secundário ao uso das antraciclinas, quimioterápicos amplamente utilizados no tratamento do sarcoma, linfoma, leucemia e câncer de mama.^24^^,^^25^

Os diferentes quimioterápicos e imunoterápicos associados à disfunção ventricular resultam em fenótipos distintos nos pacientes, desde disfunção leve assintomática e reversível até casos graves de insuficiência cardíaca clinicamente manifesta e irreversível. Sobreviventes de câncer pediátricos têm até 15 vezes mais chance de desenvolver insuficiência cardíaca que controles pareados para outros fatores de risco.^26^

A predição de cardiotoxicidade é desafiadora, dada a multiplicidade de fármacos aos quais o paciente é exposto ao longo de sua vida, além dos fatores de risco cardiovasculares muitas vezes presentes. Chamam a atenção as múltiplas interações medicamentosas das diversas modalidades terapêuticas, como os regimes de antraciclinas com ciclofosfamida e antraciclinas com trastuzumabe.

Nos últimos anos, com a introdução de novas drogas quimioterápicas e o advento da imunoterapia, além da introdução de protocolos de detecção precoce, houve aumento crescente no diagnóstico de disfunção ventricular. Na Tabela 3, observam-se os fármacos antineoplásicos mais associados à disfunção ventricular.

**Tabela 3:** Agentes quimioterápicos associados à disfunção ventricular

Agentes quimioterápicos	Incidência (%)
**Antraciclinas (dose-dependente)**	
Doxorrubicina (Adriamicina) 400 mg/m^2^ 550 mg/m^2^ 700 mg/m^2^	3-5 7-26 18-48
Idarrubicina > 90 mg/m^2^	5-18
Epirrubicina > 900 mg/m^2^	0,9-11,4
Mitoxantrona > 120 mg/m^2^	2,6
Doxorrubicina lipossomal >900 mg/m^2^	2
**Agentes alquilantes**	
Ciclofosfamida	7-28
Ifosfamida < 10 g/m^2^ 12,5-16 g/m^2^	0,5 17
**Antimetabólitos**	
Clofarabina	27
**Agentes antimicrotúbulos**	
Docetaxel	2,3-13
Paclitaxel	< 1
**Terapias-alvo anti-HER2**	
Trastuzumabe	1,7-20,1
Pertuzumabe	0,7-1,2
**Anticorpo monoclonal**	
Bevacizumabe	1,6-4
**Inibidores de tirosina quinase**	
Sunitinibe	2,7-19
Pazopanibe	7-11
Sorafenibe	4-8
Dasatinibe	2-4
Imatinibe	0,2-2,7
Lapatinibe	0,2-1,5
Nilotinibe	1
**Inibidores de proteassoma**	
Carfilzomibe	11-25
Bortezomibe	2-5

HER2: receptor tipo 2 do fator de crescimento epidérmico humano. Adaptado de Zamorano et al.^22^

### 4.1. Antraciclinas

As antraciclinas constituem um grupo de antineoplásicos reconhecidamente eficazes no tratamento de linfomas, leucemias, sarcomas e câncer de mama. Seu uso clínico é limitado por cardiotoxicidade caracterizada por disfunção ventricular e insuficiência cardíaca, sendo essa a principal causa de mortalidade nos sobreviventes do câncer.

A toxicidade por antraciclinas é bastante variável, podendo ocorrer em até 50% dos pacientes, a depender de fatores de risco do paciente e relacionados às propriedades farmacológicas dos quimioterápicos, como dose cumulativa. Por exemplo, a doxorrubicina é associada a uma incidência de 5% de insuficiência cardíaca com dose cumulativa de até 400 mg/m2, mas essa incidência pode chegar a 50% se a dose cumulativa de doxorrubicina ultrapassar 700 mg/m^2^.^27^ Estudo recente com 2.625 pacientes em seguimento de 5 anos demonstrou incidência geral de cardiotoxicidade por antraciclinas em torno de 9%, sendo que 98% dos casos ocorreram no primeiro ano e foram assintomáticos.^24^

A cardiotoxicidade pode ser aguda, precoce ou tardia, reversível ou irreversível. A toxicidade aguda é caracterizada pela presença de arritmia supraventricular, disfunção ventricular esquerda e alterações eletrocardiográficas, que surgem logo após a infusão da antraciclina em até 1% dos pacientes, sendo em geral reversível. A disfunção ventricular aguda pode ser um preditor de insuficiência cardíaca que poderá ocorrer de forma subaguda ou crônica. A cardiotoxicidade precoce surge no primeiro ano do tratamento, enquanto a tardia, anos após o tratamento (em média, 7 anos após término do tratamento).^28^

Não há preditores capazes de identificar se a toxicidade das antraciclinas será reversível ou irreversível. A elevação de biomarcadores e sua persistência podem identificar pacientes de alto risco para irreversibilidade.^29^

A propensão para cardiotoxicidade varia com os diferentes regimes de tratamento, sendo a doxorrubicina a antraciclina mais comumente associada a disfunção ventricular. A cardiotoxicidade é dose-dependente, sendo a redução da dose cumulativa uma maneira de minimizá-la. Modificações na infusão, como prolongar sua duração, fracionar a dose e utilizar formulações lipossomais, têm potencial de prevenir cardiotoxicidade.^24^ Recente estudo experimental sugeriu que o pré-condicionamento isquêmico possa ser eficaz na prevenção da cardiotoxicidade.^30^

Estudos mecanísticos demonstram que a disfunção ventricular relacionada às antraciclinas está associada a: 1) lesão do retículo sarcoplasmático e das mitocôndrias; 2) modificação estrutural e funcional de miofibrilas; 3) perda total ou parcial da matriz intercalada com placas de colágeno no interstício; 4) modificação do acoplamento excitação-contração e do fluxo do cálcio; 5) apoptose; 6) alterações do metabolismo do ferro; e 7) perda da capacidade de regeneração do músculo cardíaco e de células endoteliais coronarianas. Consequentemente, há disfunção e hipertrofia dos miócitos remanescentes.^31^ O gatilho comum desses eventos parece estar ligado ao estresse oxidativo causado pela produção de espécies reativas de oxigênio, além da inibição da topoisomerase 2β, resultando em dano às membranas, proteínas e DNA. Algumas observações dão consistência à importância do estresse oxidativo na cardiotoxicidade das antraciclinas: a) super expressão da metalotioneína, um antirradical livre, no coração de camundongo transgênico minimiza a injúria induzida pela doxorrubicina; b) a inibição da formação de peroxinitrito, um oxidante reativo produzido do óxido nítrico e do superóxido, melhora a função cardíaca de camundongos expostos a doxorrubicina; c) o probucol, um forte antioxidante, impede a redução em glutationa peroxidase e reduz a peroxidação lipídica miocárdica associada à doxorrubicina em modelo murino; d) o dexrazoxano é um quelante EDTA-*like* que pode impedir o dano por antraciclinas por meio da ligação com ferro, que é o cofator para os radicais livres.^32^ Disfunção diastólica por toxicidade cumulativa dose-dependente pode ser observada com dose cumulativa equivalente a 200 mg/m^2^, enquanto disfunção sistólica é observada usualmente com doses acima de 400 mg/m^2^, com variabilidade segundo limiar individual. Entretanto, prejuízo na função diastólica foi observado com dose cumulativa de apenas 120 mg/m^2^.^33^

Fatores de risco associados com maior chance de toxicidade por antraciclinas estão apontados na Tabela 4. Dentre eles, destacam-se cardiopatia prévia, dose cumulativa e velocidade rápida de infusão do fármaco. Entretanto, na presença dos mesmos fatores de risco, nota-se importante variabilidade entre os pacientes em relação à ocorrência de cardiotoxicidade, possivelmente relacionada a fatores genéticos e a interações com outros fatores desconhecidos.

**Tabela 4:** Fatores de risco para cardiotoxicidade relacionada às antraciclinas

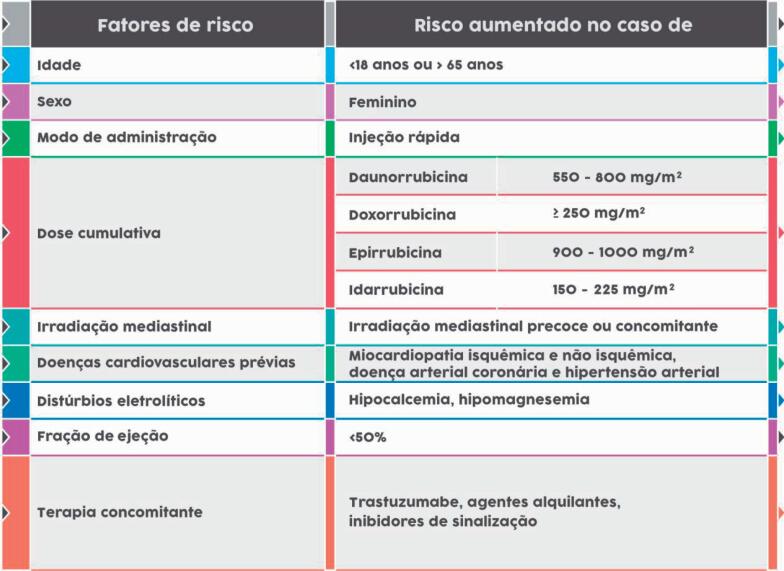

Polimorfismos nos genes transportadores de cassetes de ligação de ATP (ABC) são associados a cardiomiopatia da antraciclina. Tais transportadores são importantes na resistência às drogas via efluxo celular, incluindo antraciclinas. Atividade reduzida pode levar ao acúmulo intracelular de antraciclina e toxicidade celular. Variantes nessa família de genes replicados nas coortes de cânceres pediátricos incluem ABCC5 (A-1629T, rs7627754), associado à redução substancial na FEVE em sobreviventes homozigóticos para o alelo T.^34^ Além disso, uma variante na metiltransferase de histamina (HNMT, rs17583889) confere risco em pacientes jovens expostos às antraciclinas.^35^ Na Tabela 5, estão descritas as variantes farmacogenéticas que predispõem à cardiotoxicidade relacionada às antraciclinas.

**Tabela 5:** Variantes farmacogenéticas associadas à cardiotoxicidade das antraciclinas

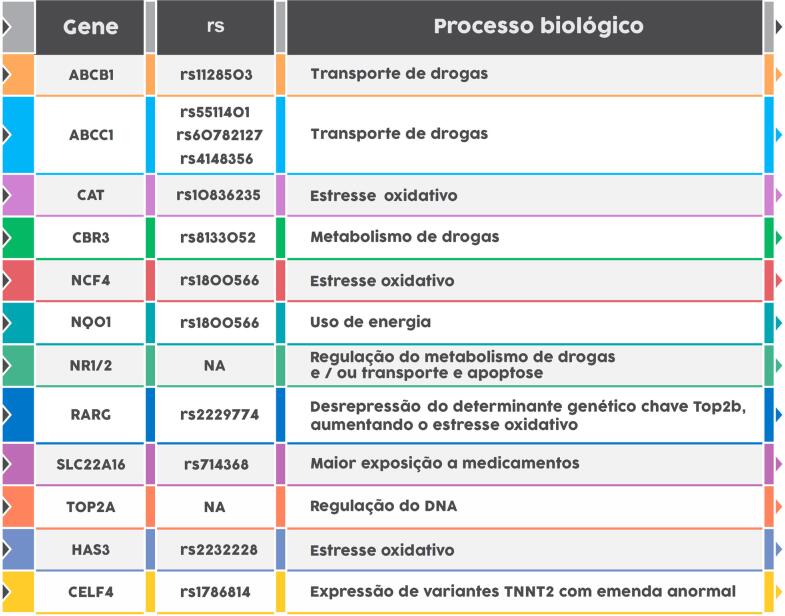

**Figura 4:**
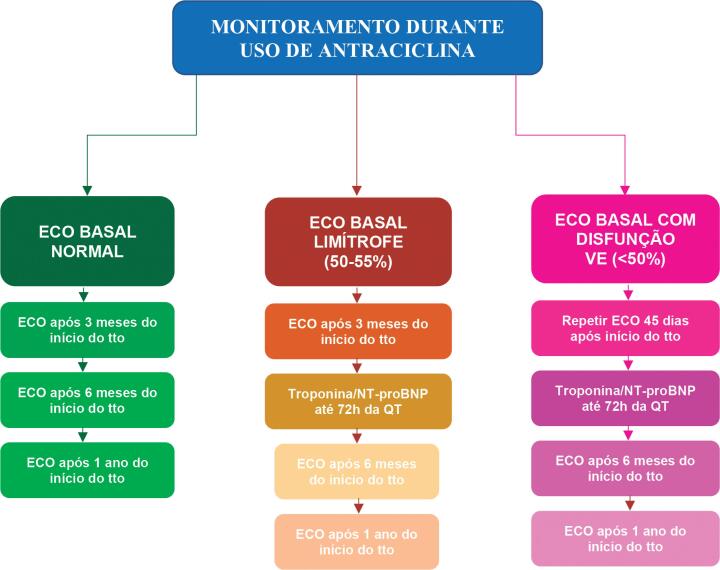
Monitoramento ecocardiográfico e análise de biomarcadores em pacientes em uso de antraciclinas. ECO: ecocardiograma; NT-proBNP: fragmento N-terminal do peptídeo natriurético tipo B; QT: quimioterapia; tto: tratamento.

Durante o tratamento com antraciclinas, monitoramento clínico e ecocardiográfico é recomendado em periodicidade pré-estabelecida ou fora do protocolo, se surgirem sinais e sintomas de insuficiência cardíaca.^21^ Idealmente, o ecocardiograma deve incluir análise de função sistólica biventricular e avaliação da função diastólica (I, A) (Figura 4).

### 4.2. Terapias-alvo Anti-HER2

O trastuzumabe é um anticorpo monoclonal cujo alvo é o receptor 2 do fator de crescimento epidérmico humano (HER2 ou ErbB2). Para os 15-20% de pacientes com câncer de mama cujos tumores super expressam o HER2, a terapia com trastuzumabe reduz de maneira significativa a mortalidade.^36^^,^^37^ Seu uso está associado a um risco considerável de cardiotoxicidade, clinicamente manifestada por um declínio assintomático da FEVE e, mais incomumente, pela ocorrência de insuficiência cardíaca sintomática.^38^ Após a introdução do trastuzumabe, três outros agentes anti-HER2 foram desenvolvidos: lapatinibe, um inibidor de tirosina quinase do fator de crescimento epidérmico (EGFR), ERBB1 e HER2; ado-trastuzumabe entansina (T-DM1), um anticorpo conjugado composto por trastuzumabe, um ligante tioéster e um derivado antimitótico de maitansina; e pertuzumabe, um anticorpo monoclonal que se liga ao subdomínio II do domínio extracelular HER2 e previne a homo- e a heterodimerização do HER2 com outros receptores HER. Embora os dados ainda sejam escassos sobre esses novos fármacos, a evidência até o momento é que o T-DM1 e o pertuzumabe sejam menos cardiotóxicos que o trastuzumabe.^39^

A taxa de declínio da FEVE consequente ao uso do trastuzumabe é variável na literatura. Estudos mais recentes apontam para taxas entre 15% e 40% de ocorrência de redução de pelo menos 10% na FEVE relacionada ao uso do trastuzumabe e, em 18% dos pacientes, a FEVE cai para menos de 53%.^40^^,^^41^ A ocorrência de insuficiência cardíaca sintomática é relatada em 0,6% a 8,7% dos pacientes.^40^

Uma das características da toxicidade dos agentes anti-HER2 que diferem da cardiotoxicidade das antraciclinas é sua reversibilidade na maioria dos casos. Os determinantes da reversibilidade são a função cardiovascular prévia e a extensão do declínio da FEVE relacionado ao tratamento. Estudo recente demonstrou que todos os declínios de FEVE menores de 10% foram reversíveis. Porém, em casos de quedas da FEVE maiores que 10%, a reversibilidade foi observada em 91% dos pacientes com função cardiovascular basal normal e em apenas 71,4% daqueles com FEVE reduzida previamente à exposição.^42^ Estudos apontam que, mesmo na presença de cardiotoxicidade, 70% a 80% dos pacientes continuam recebendo trastuzumabe e que o subgrupo que apresenta maior chance de toxicidade e mortalidade cardiovascular relacionada ao tratamento é formado por pacientes com FEVE reduzida previamente.^43^

A disfunção ventricular e a insuficiência cardíaca clinicamente manifesta induzidas pelo trastuzumabe são geralmente reversíveis após a interrupção da quimioterapia e/ou após início do tratamento da insuficiência cardíaca. Os mecanismos de cardiotoxicidade induzida pela terapia anti-HER2 incluem alterações estruturais e funcionais nas proteínas contráteis e nas mitocôndrias, mas raramente levam à morte celular, explicando a potencial reversibilidade. A interrupção do tratamento com trastuzumabe está associada a aumento da recorrência do câncer, sendo a cardiotoxicidade a maior responsável pela suspensão do fármaco.^44^

Na Tabela 6, citam-se os fatores de risco para cardiotoxicidade da terapia anti-HER2.

**Tabela 6:** Terapia anti-HER2 e fatores de risco de cardiotoxicidade.

Agentes	Fatores de risco de cardiotoxicidade
Anti-HER2 Trastuzumabe Pertuzumabe T-DM1	Tratamento prévio ou concomitante com antraciclina
	Idade > 50 anos
	Índice de massa corpórea > 30 kg/m^2^
	Disfunção ventricular esquerda prévia
	Hipertensão arterial
	Radioterapia mediastinal prévia

HER2: receptor tipo 2 do fator de crescimento epidérmico humano; T-DM1: ado-trastuzumabe entansina.

**Figura 5:**
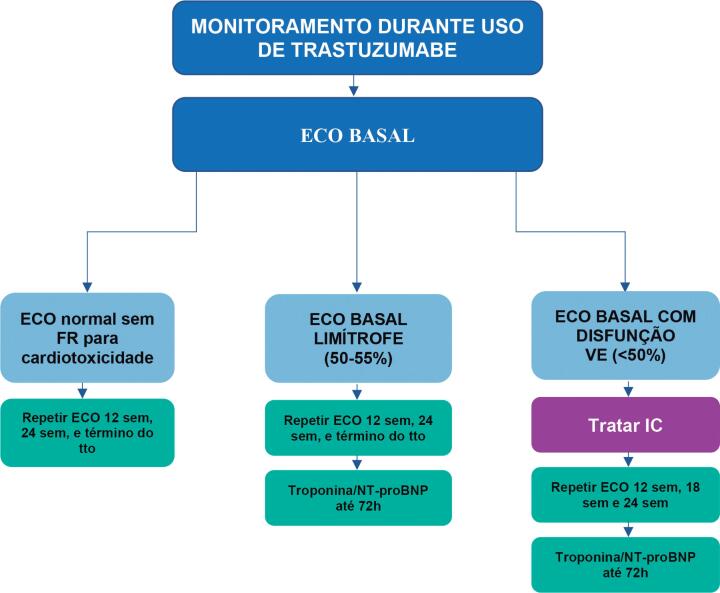
Monitoramento ecocardiográfico e análise de biomarcadores em pacientes em uso de fármacos anti-HER2. ECO: ecocardiograma; FR: fatores de risco; IC: insuficiência cardíaca; sem: semanas; tto: tratamento; VE: ventricular esquerda.

Durante o tratamento com trastuzumabe, recomenda-se monitoramento clínico e ecocardiográfico, respeitando-se a periodicidade ou se surgirem sinais e sintomas de insuficiência cardíaca (I, A) (Figura 5).

### 4.3. Inibidores VEGF

A inibição das vias de sinalização VEGF beneficia milhares de pacientes com câncer, mas alguns dos quimioterápicos dessa classe estão ligados a risco de cardiotoxicidade, que pode ser reversível ou irreversível, particularmente quando associados a tratamento concomitante ou prévio com outros quimioterápicos.^45^^–^^47^

Os pacientes tratados com bevacizumabe apresentam risco de 4,74 (IC 95%: 1,6-11,18, p = 0,001) de desenvolverem quadros de insuficiência cardíaca congestiva quando comparados ao grupo placebo.^45^ Fármacos como sunitinibe, pazopanibe e axitinibe também têm sido relacionados ao desenvolvimento de disfunção ventricular. Uma meta-análise incluindo um total de 10.553 pacientes observou uma incidência de insuficiência cardíaca congestiva de 3,2% (IC 95%: 1,8% - 5,8%) com o uso de inibidor de tirosina quinase VEGF.^47^

Hipertensão arterial sistêmica (HAS) é complicação comum dessa classe de quimioterápicos e estudos sugerem que o tratamento adequado da HAS possa reduzir o risco de insuficiência cardíaca.^48^ O prognóstico de pacientes que desenvolvem cardiotoxicidade associada aos inibidores VEGF é difícil de analisar, pois os candidatos a esses fármacos geralmente apresentam doença metastática e expectativa de vida reduzida. A maioria dos casos é reversível com o tratamento da disfunção ventricular. Os fatores de risco para cardiotoxicidade estão descritos na Tabela 7.

**Tabela 7:** Terapia com inibidores VEGF e fatores de risco de cardiotoxicidade

Inibidores VEGF	
Anticorpos Bevacizumabe Ramucirumabe	IC pré-existente, doença coronária, doença valvar, cardiopatia isquêmica Uso prévio de antraciclina
Inibidores tirosina quinase Sunitinibe Pazopanibe Axitinibe Neratinibe Afatinibe Sorafenibe Desatinibe	Hipertensão arterial Doença cardíaca pré-existente

*IC: insuficiência cardíaca.*

#### 4.3.1. Inibidores Tirosina Quinase BCR-ABL

Fármacos como os inibidores de tirosina quinase BCR-ABL modificaram o prognóstico de pacientes com leucemia mieloide crônica e tumores estromais gastrointestinais. Não há confirmação de cardiotoxicidade envolvendo imatinibe, porém nilotinibe e ponatinibe podem estar associados à cardiotoxicidade do tipo insuficiência cardíaca, HAS, arritmias e tromboembolismo.^49^

### 4.4. Terapias para Mieloma Múltiplo

Os inibidores de proteassoma são drogas relativamente novas no tratamento do mieloma múltiplo. Bortezomibe e carfilzomibe são os fármacos dessa classe e podem causar disfunção cardiovascular. Os proteassomas são complexos proteicos responsáveis por degradar proteínas disfuncionais e, além disso, essenciais para a sobrevivência do cardiomiócito. A incidência de insuficiência cardíaca com bortezomibe é de 4% e pode ser agravada pelo uso de esteroides.^50^ Carfilzomibe é um inibidor irreversível e o mais potente dos proteassomas, podendo causar insuficiência cardíaca em até 25% dos pacientes.^51^^,^^52^

### 4.5. Inibidores BRAF e MEK

A combinação da terapia com inibidores BRAF e MEK é, no momento, a primeira escolha no melanoma metastático com a mutação BRAF, apresentando significativa melhora na sobrevida dos pacientes. Atualmente, 3 inibidores BRAF (dabrafenibe, vemurafenibe e encorafenibe) e 3 inibidores MEK (trametinibe, cobimetinibe e binimetinibe) estão aprovados para o tratamento do melanoma.^53^^–^^55^

Efeitos adversos cardiovasculares associados a esses inibidores têm sido relatados em vários estudos, especialmente redução na FEVE (5-11%), HAS (10-15%) e prolongamento do intervalo QT.^56^^,^^57^ A inibição do BRAF e do MEK interfere com a sinalização cardiovascular MAPK, resultando em estresse oxidativo, apoptose de miócito e inibição de angiogênese.^56^^,^^57^

Em meta-análise recente que inclui 5 estudos clínicos randomizados e 2.317 pacientes com melanoma em uso dos inibidores BRAF e MEK, demonstrou-se que o tratamento concomitante com esses inibidores está associado a risco aumentado de embolia pulmonar (4,4x), queda na FEVE (3,72x) e HAS (1,5x). Não houve aumento da ocorrência de arritmias, infarto e prolongamento do QT. Maior risco de insuficiência cardíaca foi detectado em pacientes com idade inferior a 55 anos.^58^

### 4.6. Taxanos

O paclitaxel e o docetaxel são usados no tratamento de várias neoplasias sólidas. Cardiotoxicidade é fenômeno pouco frequente nesse grupo, com ocorrência de 12 por 100 (RR: 0,9 [0,53 -1,54]).^59^ O docetaxel, em particular, parece estar associado a aumento da ocorrência de disfunção ventricular. Alguns relatos sugerem que os taxanos devem ser evitados em pacientes com disfunção ventricular prévia, com os mesmos critérios de não utilização de antraciclinas. Há relatos de que os taxanos causam bradicardia sinusal, bloqueios atrioventriculares, taquicardia ventricular e extrassístoles ventriculares. Porém, como os taxanos são usados em combinação com antraciclinas, é desafiador afirmar seu potencial de cardiotoxicidade.^36^^,^^60^

### 4.7. Inibidores de *Checkpoint* Imunológicos

**Figura 6:**
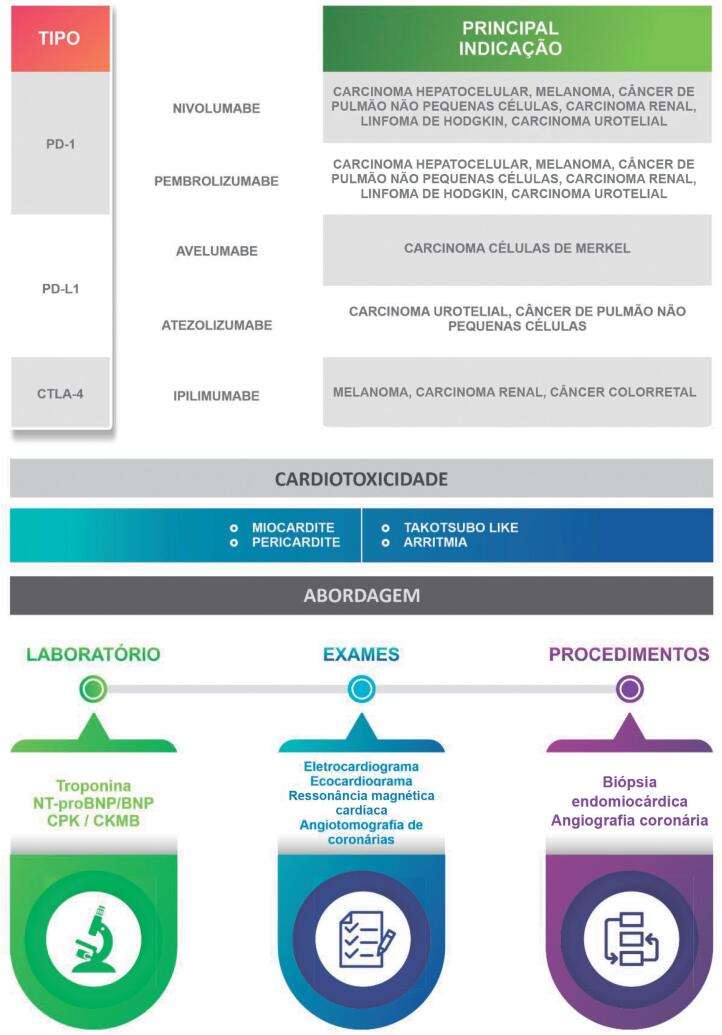
Principais inibidores de checkpoint imunológicos relacionados a toxicidade cardíaca e sua abordagem.

Os ICIs revolucionaram o tratamento do câncer. Esses imunoterápicos atuam modulando o sistema imunológico, inibindo a apoptose dos linfócitos T, gerando restauração da resposta celular antitumoral. Sua ação anti-apoptótica dá-se por inibição do CTLA-4 (ipilimumabe), do PD-1 (nivolumabe, pembrolizumabe) e do PDL-1 (atezolizumabe, durvalumabe, avelumabe)^61^ (Figura 6).

A cardiotoxicidade dos ICIs pode ser agrupada em duas categorias: efeitos adversos inflamatórios (miocardite, pericardite e vasculite) e toxicidade cardiovascular não inflamatória (síndrome Takotsubo-*like*, disfunção ventricular assintomática não inflamatória e arritmias). A maioria dos casos relatados é grave, com taxas de mortalidade de 50% na miocardite, 21% na doença pericárdica e 6% na vasculite.^62^ As principais causas de mortalidade da miocardite são arritmias e choque cardiogênico.^62^^–^^64^

Os eventos adversos ocorrem normalmente após a primeira ou segunda dose dos ICIs, mas há relatos esporádicos de eventos cardiovasculares até 32 semanas após o tratamento. A prevalência de envolvimento cardiovascular é maior em pacientes com terapia combinada, sexo feminino e idade acima de 75 anos. A prevalência de miocardite varia entre 0,06% e 0,3%.^62^^,^^63^

Para pacientes que desenvolvem novos sintomas cardiovasculares durante ou logo após o tratamento com os ICIs ou que apresentam arritmia, anormalidade do sistema de condução ou disfunção ventricular ao ecocardiograma, recomenda-se iniciar investigação cardiovascular com dosagem de biomarcadores (troponina, NT-proBNP e proteína C reativa), ECG, painel viral, ecocardiograma com *strain* e RMC para confirmação diagnóstica e exclusão de miocardite viral (IIa, C).

Biópsia endomiocárdica deve ser considerada quando há suspeição diagnóstica mesmo com investigação inicial negativa (IIa, C).

## 5. Radioterapia

A incidência atual de cardiotoxicidade induzida por radiação é difícil de ser estimada devido a algumas razões, como longo intervalo entre a exposição e a manifestação clínica de cardiotoxicidade, uso de quimioterapia cardiotóxica concomitante e melhoria progressiva nas técnicas de radiação nos últimos anos, com redução da incidência de lesão estrutural cardíaca. Os estudos apontam risco relativo de eventos cardiovasculares fatais em 2,2% a 12,7% de sobreviventes de linfoma e em 1% a 2,2% de pacientes com câncer de mama.^65^^,^^66^ Entre os sobreviventes expostos a radioterapia, o risco de disfunção ventricular aumenta 4,9 vezes.^66^ Na neoplasia de mama, é mais frequente cardiotoxicidade relacionada a radioterapia em pacientes com lesão em hemitórax esquerdo^67^ e naquelas em uso concomitante de antraciclinas. A lesão induzida pela radioterapia pode afetar o músculo cardíaco, as válvulas, o pericárdio, as coronárias e o sistema de condução,^68^ podendo ser diagnosticada 10 a 15 anos após a radioterapia.

## 6. Prevenção e Tratamento da Cardiotoxicidade

**Figura 7:**
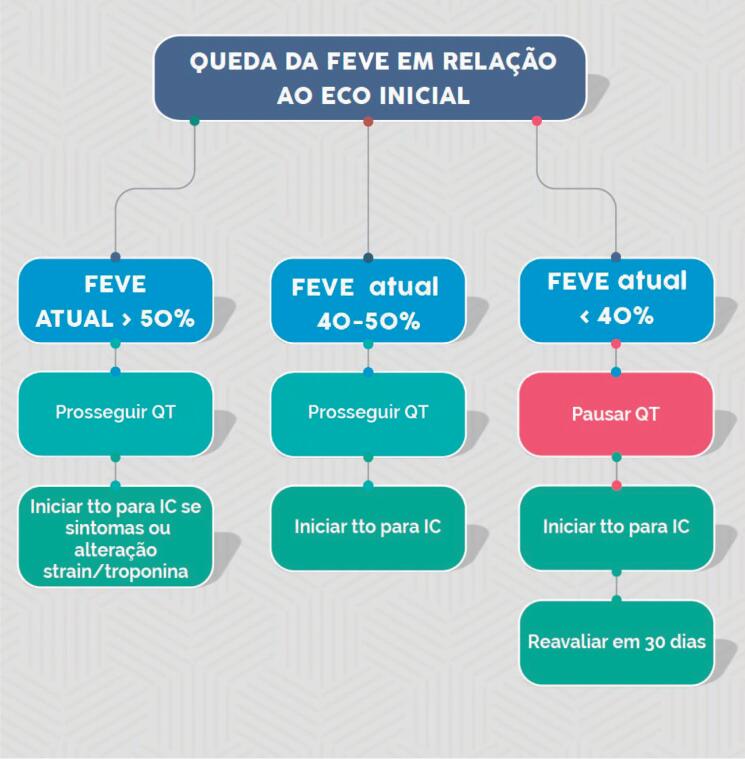
Algoritmo de manejo da insuficiência cardíaca e disfunção ventricular por antraciclinas. ECO: ecocardiograma; FEVE: fração de ejeção do ventrículo esquerdo; QT: quimioterapia; IC: insuficiência cardíaca; tto: tratamento.

- A prevenção da cardiotoxicidade deve ser realizada em todos os pacientes com câncer, com o reconhecimento dos fatores de risco cardiovasculares desde a consulta inicial, sendo recomendadas as seguintes medidas: cessação de tabagismo, cessação do alcoolismo, implementação de dieta regular visando à manutenção do peso adequado (índice de massa corpórea entre 18 e 24 kg/m^2^), prática de exercícios físicos (atividade física aeróbica moderada por 30 minutos por dia por pelo menos 5 vezes na semana), controle da HAS, tratamento do diabetes e da dislipidemia (I, B).
- Os fármacos de escolha para o tratamento da HAS são os inibidores da enzima conversora de angiotensina (IECA) ou os antagonistas do receptor da angiotensina (AT1). As estatinas são recomendadas no tratamento da dislipidemia, com o objetivo de manter níveis de LDL abaixo de 100 mg/dl. O tratamento do diabetes tem como fármaco de escolha a metformina e, em casos de insuficiência cardíaca associada, devem-se utilizar os inibidores do SGLT2 (empaglifozina, dapaglifozina, canaglifozina) e, em casos de doença coronariana, dar preferência aos agonistas do GLP-1 (liraglutida, dulaglutida e semaglutida) (IIa, C).
- Na avaliação da proposta terapêutica, devem ser reconhecidos os fatores de risco para cardiotoxicidade e implementadas as medidas específicas de acordo com o regime (IIa, C).
- Para pacientes com detecção de cardiotoxicidade subclínica (elevação de troponina ou redução no SLG absoluta ≥ 5% ou relativa ≥ 15%):
  - o uso de IECA ou de AT1 ou de betabloqueador pode ser considerado com o objetivo de prevenir disfunção ventricular e eventos cardiovasculares (IIa, B);
  - repetir ecocardiografia com *strain* a cada 3 meses e dosagem de biomarcadores a cada ciclo, se assintomático, ou a qualquer momento, se surgirem sintomas (IIa, C);
  - quimioterapia não deve ser suspensa com base em alterações no *strain* e em biomarcadores (IIa, C);
  - considere encaminhar o paciente para o cardio-oncologista (IIa, C);
  - considere excluir doença isquêmica do coração (IIa, C);
  - considere iniciar dexrazoxano em pacientes que serão submetidos a doses altas de antraciclinas e com risco elevado de cardiotoxicidade (IIa, B).
- Em pacientes com FEVE ≤ 50% e ≥ 40%, terapia com IECA/AT1 e betabloqueador é recomendada antes do tratamento cardiotóxico (I, A).
- Pacientes com FEVE ≤ 40% não devem receber terapia com antraciclina a menos que não haja opções eficazes de tratamento (IIa, A).
- Pacientes em uso de quimioterapia ou imunoterapia que desenvolvem insuficiência cardíaca e FEVE < 40%, durante o tratamento, devem ter o tratamento antineoplásico suspenso temporariamente de acordo com discussão entre o cardiologista e o oncologista, e terapia para insuficiência cardíaca deve ser iniciada de acordo com as diretrizes e consensos (I, A).
- Pacientes em uso de fármacos com potencial de cardiotoxicidade que apresentarem sinais ou sintomas de insuficiência cardíaca, devem ser encaminhados ao cardio-oncologista para avaliação clínica, realização de ecocardiograma e dosagem de biomarcadores (IIa, C).
- As Figuras 7 e 8 apresentam os algoritmos de manejo da disfunção ventricular por antraciclinas e anti-HER2 que devem ser considerados (IIa, B).
- Em pacientes com cardiotoxicidade por trastuzumabe, após a estabilização dos sintomas e a recuperação da FEVE para acima de 40%, a reintrodução do trastuzumabe deve ser considerada, desde que o paciente esteja sendo acompanhado pelo cardio-oncologista, com avaliação seriada por ecocardiografia e biomarcadores (IIa, B).
- Em pacientes com cardiotoxicidade por trastuzumabe, não havendo melhora dos sintomas e persistindo FEVE abaixo de 40%, a reintrodução do trastuzumabe só deve ser considerada se não houver alternativa terapêutica após ampla discussão com oncologista (IIa, C).
- Em pacientes em uso de sunitinibe ou outro fármaco anti-VEGF, avaliação e controle adequado da HAS são recomendados (IIa, C).
- Em pacientes em uso de terapia com anticorpos monoclonais ou inibidores de tirosina quinase com ação anti-VEGF (bevacizumabe, sunitinibe, sorafenibe, axitinibe e pazopanibe), o maior risco de insuficiência cardíaca ocorre no início da terapia. Na presença de sinais e sintomas, deve-se investigar o paciente com ecocardiograma e dosagem de biomarcadores (IIa, B). Recomendam-se consultar o cardio-oncologista, iniciar tratamento para insuficiência cardíaca e suspender o fármaco em discussão com oncologista (IIa, C). Após recuperação do quadro clínico e da FEVE, considera-se reiniciar a quimioterapia (IIa, C).
- Em pacientes com insuficiência cardíaca ou com disfunção ventricular, tratamento medicamentoso deve ser instituído de acordo com as diretrizes (I, A).
- A indicação de dispositivo de assistência circulatória e de transplante cardíaco segue as recomendações da Diretriz Brasileira de Insuficiência Cardíaca Aguda e Crônica, devendo-se discutir com o oncologista, antes da indicação, o *status* do paciente e o prognóstico oncológico, levando-se sempre em consideração as preferências do paciente.
- A indicação de transplante cardíaco para pacientes com câncer segue as recomendações da Diretriz Brasileira de Insuficiência Cardíaca Aguda e Crônica (Tabela 8). Porém, devem ser considerados para transplante apenas os pacientes com insuficiência cardíaca aguda ou crônica que atendam aos critérios de remissão ou cura do câncer por um período maior que 3 anos (IIa, C).
- Havendo suspeita ou confirmação de miocardite por ICIs, a terapia com ICIs deve ser interrompida e corticosteroide iniciado imediatamente (metilprednisolona, 1g intravenoso por dia, por 3 a 5 dias, seguida de prednisona, 1-2 mg/kg/dia). O corticosteroide deve ser continuado até a resolução dos sintomas e a normalização da troponina, da função sistólica e das anormalidades de condução (IIa, C). Em casos de pericardite, recomenda-se a utilização de corticosteroide oral (IIa, C). Na síndrome de Takotsubo, pode-se considerar a pulsoterapia (IIa, C) e, na cardiomiopatia dilatada, o tratamento é de suporte (Tabela 9).
- Em pacientes com miocardite refratária ou em situações graves com choque cardiogênico, outras terapias imunossupressoras como globulina antitimócito, infliximabe (exceto em pacientes com insuficiência cardíaca), micofenolato mofetil, ciclofosfamida ou abatacepte devem ser considerados (IIa, C).
- Para pacientes com taquiarritmia ou bradiarritmia por ICIs, terapia apropriada medicamentosa e marca-passo devem ser considerados de acordo com as características clínicas (IIa, C).
- A terapia com ICIs deve ser descontinuada nos casos de miocardite. A decisão de reiniciar a terapia deve ser individualizada, de acordo com o *status* do câncer, resposta ao tratamento, gravidade da cardiotoxicidade, analisando riscos e benefícios. Se o tratamento com ICIs for reiniciado, recomendam-se monoterapia com uma droga anti-PD1 e vigilância cardiovascular (IIa,C).
- Considerar o uso do dexrazoxano em pacientes com câncer de mama metastático com dose planejada elevada de antraciclina (doxorrubicina acima de 250 mg/m^2^) (I, A) e em pacientes com sarcoma e pacientes pediátricos com linfoma/leucemia (IIa, A).

**Figura 8:**
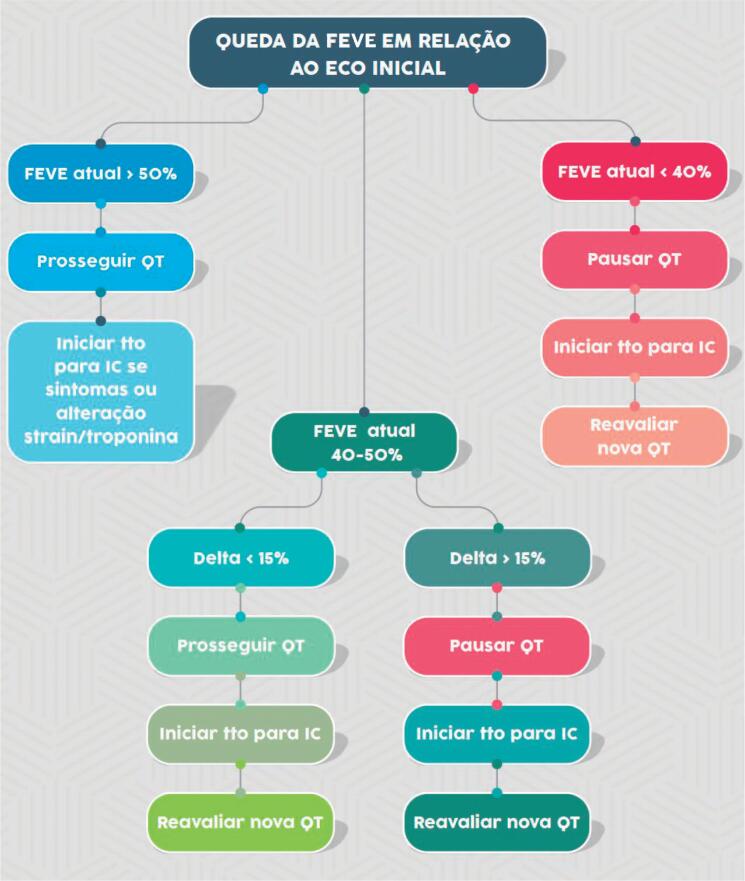
Algoritmo de manejo da insuficiência cardíaca e da disfunção ventricular por terapia anti-HER2. ECO: ecocardiograma; FEVE: fração de ejeção do ventrículo esquerdo; IC: insuficiência cardíaca; QT: quimioterapia; tto: tratamento.

**Tabela 8:** Recomendações para transplante cardíaco. Comitê Coordenador da Diretriz de Insuficiência Cardíaca. Diretriz Brasileira de Insuficiência Cardíaca Crônica e Aguda^69^

Recomendação	Classe	Nível de evidência
Em pacientes com insuficiência cardíaca aguda e/ou choque cardiogênico com baixo potencial de recuperação, sugere-se que a avaliação da candidatura ao transplante seja iniciada precocemente e que seja o mais completa possível, incluindo avaliação psicossocial, mesmo que com dificuldades inerentes ao quadro agudo.	I	C
Em pacientes com choque cardiogênico refratário e sem recuperação adequada da função miocárdica, a definição da candidatura para transplante deve considerar o grau de instabilidade hemodinâmica, a presença de disfunções multiorgânicas, as comorbidades e a experiência do centro. Escores prognósticos podem auxiliar na estimativa de risco de mortalidade pós-transplante a curto e longo prazo.	IIa	C

**Tabela 9:** Efeitos adversos dos inibidores de *checkpoint i* munológicos e estratégias terapêuticas

Potenciais eventos CV relacionados aos inibidores de *checkpoint*	Métodos diagnósticos	Potencial abordagem inicial para tratamento	Potencial terapia adicional se estável e não respondendo à abordagem inicial	Potencial terapia adicional se instável
Miocardite	Não invasivo: RMC, troponina, ECG Invasivo: biópsia e patologia	Metilprednisolona, 1g/dia por 3-5 dias, seguida de 1,5mg/kg de prednisona com monitorização ambulatorial de troponina. Terapia padrão para insuficiência cardíaca com bloqueador neuro-hormonal, se FEVE reduzida.	Micofenolato, 500-750mg 2x/dia Plasmaférese Imunoglobulina intravenosa	Globulina antitimócito Abatacepte Alentuzumabe Suporte circulatório mecânico
Pericardite	Não invasivo: ecocardiografia Invasivo: análise de líquidos	Prednisona, 1,5mg/kg/dia, com redução da dose ambulatorial por 2 meses.	Metilprednisolona, 1g/dia por 3-5 dias Micofenolato, 500-750mg 2x/dia	Drenagem pericárdica, se presença de derrame pericárdico volumoso com sinais de instabilidade hemodinâmica
Síndrome de Takotsubo	Não invasivo: ecocardiografia, RMC Invasivo: cineangiocoronariografia e ventriculografia	Terapia-padrão de insuficiência cardíaca com bloqueador neuro-hormonal, se FEVE reduzida. Considerar metilprednisolona, 1g/dia por 3-5 dias, seguida de prednisona oral com redução de dose por 4-6 semanas.	Micofenolato, 500-750mg 2x/dia	Suporte circulatório mecânico
Cardiomiopatia dilatada	Não invasivo: RMC, ecocardiografia, troponina, peptídeo natriurético Invasivo: cineangiocoronariografia e ventriculografia	Terapia-padrão de insuficiência cardíaca com bloqueador neuro-hormonal, se FEVE reduzida.	Terapia de ressincronização cardíaca Desfibrilador cardíaco implantável	

*Esta tabela detalha as toxicidades cardiovasculares associadas com os inibidores de checkpoint e potenciais estratégias de manejo. Muitas das estratégias listadas para outras toxicidades que não miocardite são extrapoladas da literatura de miocardite e baseadas em pequenas séries de casos ou relatos de casos. CV: cardiovascular; ECG: eletrocardiograma; FEVE: fração de ejeção do ventrículo esquerdo; RMC: ressonância magnética cardíaca. Tabela adaptada de Lenihan DJ et al. Proceedings*.^18^

## 7. Tromboembolismo Arterial e Venoso

A doença tromboembólica é afecção comum no paciente com câncer, sendo considerada a segunda causa de mortalidade nessa população.

### 7.1 Trombose Venosa

O tromboembolismo venoso (TEV) inclui a trombose venosa profunda (TVP) e o tromboembolismo pulmonar (TEP). É uma grave complicação em pacientes com câncer, nos quais é a segunda causa de óbito. As neoplasias são associadas ao aumento do risco e da gravidade e à recorrência da trombose, além de resultar em taxas maiores de complicações relacionadas ao tratamento. Ademais, o paciente com neoplasia tem uma chance 2 a 9 vezes maior de recorrência de eventos tromboembólicos.^70^^–^^72^

O câncer induz um estado pró-trombótico devido à sua produção de micropartículas trombogênicas, à ativação plaquetária, às suas propriedades antifibrinolíticas e à produção de trombina. Além disso, a trombogênese é potencializada por fatores relacionados ao tipo de câncer, ao *status* da doença, ao uso de drogas concomitantes,^73^ como agentes eritroestimulantes, à presença de anemia e leucocitose, à obesidade e ao fenótipo laboratorial trombogênico, como presença de altos níveis de dímero D e de fragmento 1+2 da protrombina.^74^

Nos últimos 5 anos, alguns ensaios clínicos foram publicados especificamente na população oncológica, permitindo ampliar o arsenal terapêutico desses pacientes (Tabela 10).^75^^,^^76^

**Tabela 10:** Estudos clínicos de TEV em pacientes com câncer

Estudo	População	Intervenção	Desfechos primários de eficácia	Desfecho primário de segurança
**Prevenção primária**				
CASSINI trial*	841 pacientes ambulatoriais com câncer e alto risco para TEV	Rivaroxabana 10mg vs placebo 6 meses	TVP ou EP ou morte relacionada a TEV HR: 0,66; IC95%: 0,4-1,09	Sangramento maior 1,0% vs. 2,0% HR: 1,96; IC95%: 0,59-6,49
AVERT trial*	574 pacientes ambulatoriais com câncer e alto risco para TEV	Apixabana 2,5mg 2x/dia vs placebo	TEV documentado 4,2% vs 10,2%. HR: 0,41; IC95%:0,26-0,65	Sangramento maior 3,5% vs. 1,8% HR: 2,0; IC95%:1,0-3,95.
**Tratamento**				
HOKUSAI VTE	1050 pacientes com câncer com TEV agudo sintomático ou incidental	HBPM por 5 dias + edoxabana 60mg vs dalteparina Tratamento: 6 meses	Recorrência de TEV ou sangramento maior 12,8% vs 13%	Sangramento maior 6,9% vs. 4% HR: 1,77; IC95%: 1,03-3,04
SELECT-D	406 pacientes com câncer e EP ou TEV sintomáticos	Rivaroxabana vs dalteparina Tratamento: 6 meses	Recorrência de TEV: 4% vs 11% HR: 0,43; IC95%: 0,19-0,99	Sangramento maior 6% vs. 4% HR:1,83 IC 95% 0,68-4,96
ADAM-VTE trial	300 pacientes com TEV associado ao câncer	Apixabana 10mg 2x/dia por 7 dias seguido de 5mg 2x/dia vs dalteparina	Recorrência de TEV 0,7% vs 6,3%. HR: 0,099; IC95%: 0,013-0,78	Sangramento maior 0% vs 1,4% HR: 1,96; IC95%: 0,59-6,49
Caravaggio Study*	1055 pacientes com câncer com TEV ou EP sintomáticos ou incidental	Apixabana 10mg por 10 dias seguida de 5mg/dia vs dalteparina	Recorrência TEV 5,6% vs 7,9% HR: 0,63; IC95%: 0,37-1,07	Sangramento maior 3,8% vs 4% HR: 0,82; IC95%: 0,4-1,69

* *Ensaio clínico randomizado; CASSINI = rivaroxabana em pacientes ambulatoriais de alto risco com câncer; AVERT = apixabana na prevenção de TEV em pacientes com câncer; HOKUSAI VTE = edoxabana versus dalteparina para tratamento de TEV sintomático; SELECT-D = anticoagulação em paciente com risco de recorrência de TEV; ADAM VTE = apixabana e dalteparina em TEV associado a neoplasia ativa; Caravaggio Study = apixabana para o tratamento de TEV associado ao câncer. EP: embolia pulmonar; HBPM: heparina de baixo peso molecular; HR: razão de chance; IC: intervalo de confiança; TEV: tromboembolismo venoso; TVP: trombose venosa profunda. Tabela adaptada de: Lenihan DJ et al. Proceedings*.^18^

As recomendações para o manejo do TEV no paciente com câncer são:

- A equipe multidisciplinar que acompanha os pacientes oncológicos deve educá-los quanto ao risco de TEV, particularmente em situações de alto risco, como cirurgias de grande porte e durante o tratamento quimioterápico (IIa, C).
- Pacientes hospitalizados devem receber profilaxia farmacológica, na ausência de contraindicações (IIa, B).
- Profilaxia farmacológica não deve ser feita de rotina em pacientes admitidos para pequenos procedimentos ou para infusão de quimioterapia ou para transplante (IIa, C).
- Para pacientes ambulatoriais de baixo risco, anticoagulação de rotina para prevenção de TEV não está recomendada (III, B).
- Profilaxia farmacológica ambulatorial com apixabana, rivaroxabana ou enoxaparina deve ser oferecida a pacientes de alto risco para TEV, mensurado pelo escore de Khorana (≥ 2) ou pelo CAT *score* configurando alto risco (mensuração de dímero D e avaliação do tipo de câncer) (IIa, A).
- Na avaliação para profilaxia farmacológica ambulatorial, considere o risco de sangramento do paciente (maior nos tumores gastrointestinais) e suas preferências (IIa, C).
- Pacientes com mieloma múltiplo em uso de talidomida ou lenalidomida ou dexametasona devem ser avaliados para a instituição de aspirina ou enoxaparina (IIa, C).
- Pacientes que serão submetidos a cirurgia oncológica de grande porte devem receber profilaxia farmacológica de TEV (enoxaparina ou heparina de baixo peso molecular), que deve ser iniciada no pré-operatório, com exceção de pacientes com sangramento ativo ou alto risco de sangramento (I, A). Métodos mecânicos podem ser associados à profilaxia farmacológica, porém seu uso como monoterapia só deve ser feito em pacientes com contraindicação à heparina (IIa, B).
- O regime combinado de profilaxia farmacológica e mecânica pode melhorar a eficácia, especialmente em pacientes de mais alto risco (IIa, B).
- A tromboprofilaxia farmacológica para pacientes submetidos a cirurgia oncológica de grande porte deve se estender por 7 a 10 dias, devendo ser prolongada por 4 semanas de pós-operatório em casos de cirurgia abdominal aberta ou laparoscópica e em cirurgia pélvica se o paciente tem mais fatores de risco do tipo obesidade, imobilidade e história de TEV (IIa, B).
- Em cirurgias menores ou de pequeno porte, a decisão do tempo de profilaxia deve ser personalizada (IIa, C).
- O tratamento inicial do TEV no paciente oncológico pode ser realizado com heparina de baixo peso molecular (enoxaparina), heparina não fracionada, fondaparinux, apixabana ou rivaroxabana. Para pacientes iniciando tratamento com anticoagulação parenteral, a heparina de baixo peso molecular é preferida em relação à heparina não fracionada nos primeiros dias de tratamento, desde que o paciente não tenha disfunção renal (depuração de creatinina deve ser maior que 40 ml/min/m^2^) (I, A).
- A anticoagulação a longo prazo pode ser realizada preferencialmente com heparina de baixo peso molecular, edoxabana, apixabana ou rivaroxabana por pelo menos 6 meses (I, A).
- A varfarina pode ser utilizada no paciente com câncer na indisponibilidade de outras medicações ou em situações específicas de contraindicação aos outros anticoagulantes, como na insuficiência renal crônica dialítica (IIa, B).
- Os anticoagulantes de ação direta (DOACs), como rivaroxabana e apixabana, estão associados a maiores taxas de sangramento, especialmente em neoplasias do trato gastrointestinal e geniturinárias (IIa, B).
- Devem-se analisar caso a caso as interações medicamentosas dos DOACs no paciente com câncer (I,A).
- Anticoagulação por tempo superior a 6 meses deve ser oferecida a pacientes com câncer ativo, como os metastáticos, ou em quimioterapia, devendo ser analisados risco e benefício (IIa, C).
- Com base na opinião de especialistas, na ausência de estudos randomizados, a inserção de filtro de veia cava não deve ser recomendada a pacientes com trombose crônica ou estabelecida (tempo maior que 4 semanas) nem àqueles com contraindicações temporárias à terapia anticoagulante (IIa, C).
- A varfarina é a primeira opção de anticoagulação nos pacientes com insuficiência renal crônica dialítica (IIa, B).
- O filtro de veia cava pode ser considerado em pacientes com TEV agudo (nas últimas 4 semanas), com contraindicação absoluta a anticoagulante e se o TEV for de alto risco (IIa, C)
- A TVP e o TEP incidentais devem ser tratados da mesma maneira do TEV sintomático, por terem desfechos semelhantes (IIa, C).
- O tratamento do TEP subsegmentar ou do trombo venoso visceral ou esplâncnico deve ser considerado caso a caso, analisando-se potenciais benefícios e riscos da anticoagulação (IIa, C).
- Pacientes com câncer devem ter seu risco de TEV avaliado ambulatorialmente com o escore de Khorana e o CAT *score*, devendo-se analisar os riscos e os benefícios dessa estratégia individualmente, por estar associada à redução de eventos tromboembólicos mas não à redução de mortalidade (IIa, B).
- Em casos de sangramento clinicamente significativo associado à varfarina, o tratamento de escolha é a vitamina K intravenosa (10 mg) e o complexo protrombínico intravenoso (500 U/kg) (IIa, B).
- Em casos de sangramento associado a rivaroxabana, edoxabana e apixabana, não há antídoto específico disponível. Portanto, recomenda-se a utilização de antifibrinolíticos (ácido tranexâmico, 1g a 2g intravenoso) e complexo protrombínico (500 U/kg intravenoso). Em casos de refratariedade, recomenda-se a utilização de plasma (15 ml/kg), crioprecipitado (1 U/kg) e plaquetas (1-2 unidades) por aférese (IIa, C).

Há variação substancial do risco de TEV em pacientes com câncer e diferentes situações clínicas. Os pacientes com câncer devem ter seu risco de TEV analisado na avaliação basal e periodicamente a partir de então, em particular no início da terapia antineoplásica e no momento da hospitalização. Fatores de risco individuais, incluindo biomarcadores ou sítio do câncer, não identificam com alta acurácia pacientes com câncer em risco de TEV. Ambulatorialmente, a avaliação deve ser feita por meio do escore de Khorana e do CAT *score* (IIa,C) (Tabelas 11 e 12, respectivamente).

**Tabela 11:** Escore de Khorana^77^

Característica do paciente		Pontos
Sítio do câncer		
	Risco muito alto (estômago e pâncreas)	2
	Alto risco (pulmão, linfoma, ginecológico, bexiga, testicular, renal)	1
Contagem de plaquetas pré-quimioterapia ≥ 350.000 / μl		1
Nível de hemoglobina < 10 g/dL e/ou uso de fatores de crescimento de glóbulos vermelhos		1
Contagem de leucócitos pré-quimioterapia > 11.000 / μl		1
Índice de massa corpórea ≥ 35 kg/m^2^		1
Calcular a pontuação total, adicionando pontos para cada critério do modelo		
Interpretação		
	Escore de alto risco ≥ 3 pontos	
	Escore de risco intermediário 1-2 pontos	
	Escore de baixo risco - 0 pontos	

**Tabela 12:** Nomograma (CAT *score*) para predizer o risco de TEV em 6 meses

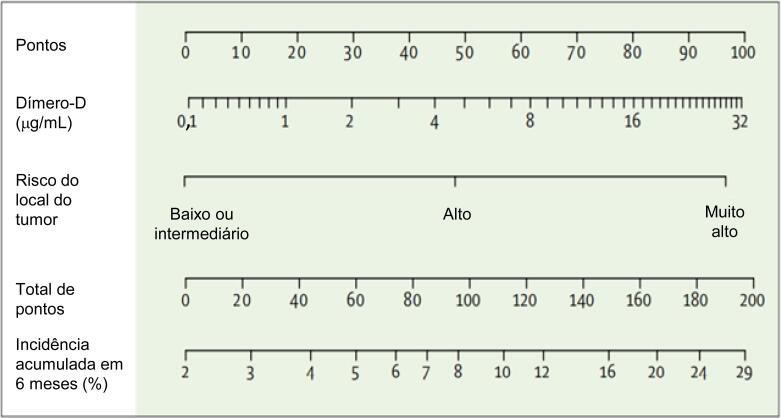

### 7.2. Trombose Arterial

Em estudo epidemiológico com 279.719 participantes e comparando pacientes com neoplasia e controles sem neoplasia, a incidência de eventos arteriais foi de 4,7% nos primeiros e 2,2% nos controles em um período de 6 meses.^79^ Geralmente, esses eventos ocorrem em indivíduos com neoplasias metastáticas de pâncreas, mama, colorretal e pulmão em uso de antraciclinas, taxanos e platina. O estado pró-trombótico pode favorecer eventos embólicos secundários à fibrilação atrial. Alguns fármacos antineoplásicos, especialmente inibidores VEGF, podem induzir complicações tromboembólicas. Em pacientes sob terapia hormonal, maiores taxas de eventos trombóticos arteriais são observadas mais frequentemente com os inibidores de aromatase do que com o tamoxifeno. Em muitos casos, quinases e suas vias desempenham papel crítico na homeostase vascular e metabólica das células. A inibição dessas quinases pode causar sequelas cardiovasculares, dependendo do tipo de quinase. As toxicidades vasculares mais preocupantes que podem ocorrer com os novos agentes incluem eventos isquêmicos arteriais, como infarto agudo do miocárdio, acidente vascular cerebral e isquemia de membro, assim como eventos tromboembólicos venosos.^79^

Relatos recentes mostram que a terapia com inibidor de VEGF resulta em eventos vasculares adversos, como dissecção de aorta, acidente vascular cerebral e trombose arterial e venosa. O bevacizumabe é associado a maior taxa de TEV dentre os inibidores VEGF, aproximadamente 12% comparado a 2% com os outros fármacos.^49^^,^^80^

## 8. Síndrome Metabólica Associada à Terapia de Privação Androgênica

O tratamento de neoplasias de próstata localmente avançada baseia-se no controle hormonal de testosterona. Esse bloqueio pode ser obtido cirurgicamente (orquiectomia) ou pela terapia medicamentosa de privação androgênica. Os agonistas de GnRH (hormônio liberador de gonadotrofina: leuprolide, goserelina e triptorelina) e os antagonistas de GnRH (degarelix) causam bloqueio central com redução dos níveis de hormônio luteinizante, hormônio folículo estimulante e testosterona. Os inibidores de receptor androgênico adrenal (abiraterona) e os inibidores diretos androgênicos (enzalutamida) também agem reduzindo a testosterona. São medicações utilizadas com intenção curativa em pacientes de alto risco com doença não metastática e como terapia padrão na doença metastática. Entender o impacto dessas medicações no risco cardiovascular é importante, pois muitos dos fatores de risco que levam ao câncer de próstata também podem resultar em doença cardiovascular, como idade avançada, tabagismo, dieta e obesidade. Estudos apontam detecção mais prevalente desses fatores de risco na população de pacientes com câncer de próstata.

A terapia antiandrogênica reconhecida leva a alterações metabólicas caracterizadas por hiperinsulinemia, hipercolesterolemia e alterações na composição corporal, com aumento da gordura predominantemente visceral e redução de massa magra. A síndrome metabólica resultante da terapia antiandrogênica está associada a aumento de complicações cardiovasculares. Recomenda-se a modificação dos fatores de risco com terapia hipolipemiante, tratamento anti-hipertensivo, controle estrito da glicemia e utilização de antiagregantes plaquetários (IIa, B).

## 9. Arritmia Cardíaca

Diversos fatores presentes nos pacientes com câncer, como infecção, distúrbios hidroeletrolíticos, desidratação, procedimentos cirúrgicos e terapias oncológicas e adjuvantes, predispõem à ocorrência de arritmias cardíacas.^81^ Essas arritmias são complicações relativamente frequentes em pacientes com câncer e estima-se que ocorram em 16-36% desses pacientes.^82^^,^^83^

Os tipos de arritmias cardíacas no paciente oncológico incluem amplo espectro: taquicardia sinusal, bradiarritmias, taquiarritmias e distúrbios de condução. Dentre as arritmias supraventriculares, a mais comum é a fibrilação atrial. Taquicardia ventricular e fibrilação ventricular são raras, mas podem ocorrer especialmente na presença do alargamento do QT e em pacientes com hipocalemia ou hipomagnesemia.^84^^,^^85^ A Tabela 13 apresenta os principais fármacos e a incidência de arritmias cardíacas.

**Tabela 13:** Principais fármacos relacionados a arritmias cardíacas e suas incidências

Fármaco	Incidência
Antraciclinas	Alterações eletrocardiográficas: 38,6% FA: 2-10%
Agentes antimicrotúbulos (Paclitaxel)	Bradicardia sinusal: 29% BAV 1° grau: 25%
Antimetabólitos (5-Fluorouracil e Capecitabina)	Alterações eletrocardiográficas: 68% Arritmias (FA, TSV, TV): 5%
Platina (Cisplatina)	TSV: 12-32%
Talidomida	Bradicardia: 27%
Trióxido arsênio	Prolongamento QT e arritmias ventriculares: até 50%
**Inibidor Tirosina Quinase**	
Crizotinibe	Alargamento QT e arritmias: 3,5%
Dasatinibe	Alargamento QT e arritmias: 0,6-3%
Sunitinibe	Alargamento QT e arritmias: 1-4%
Vandetinibe	Alargamento QT e arritmias: 12-15%

*BAV: bloqueio atrioventricular; FA: fibrilação atrial; TSV: taquicardia supraventricular; TV: taquicardia ventricular.*

### 9.1. Prolongamento QT

O diagnóstico do prolongamento do intervalo QT é eletrocardiográfico, devendo-se calcular o QTc pela fórmula de Bazett QT / (RR)^1/2^ ou Fridericia QT / (RR)^1/3^. Consideram-se normais valores de QTc ≤ 440 ms nos homens e entre 450 e 460 ms nas mulheres. Tanto fatores congênitos como adquiridos podem ser responsáveis por prolongamento do QT. Dentre as condições mais citadas estão: sexo feminino, bradicardia, anormalidades eletrolíticas, efeitos de drogas, isquemia miocárdica, insuficiência cardíaca, miocardites, hipotermia e canalopatias.^86^

O prolongamento do QT é uma preocupação em pacientes com câncer, pois tanto o tratamento oncológico como os distúrbios hidroeletrolíticos e as medicações concomitantes podem contribuir para esse prolongamento e para a predisposição à ocorrência de arritmias complexas.^83^ A monitorização do QT e a correção de fatores que contribuam para o prolongamento do QT devem ser consideradas em pacientes em uso de medicações que aumentem o intervalo QT. A cardiotoxicidade é definida quando há prolongamento de QTc > 500ms e/ou variação do QT > 60ms do basal (Tabela 14).^87^^,^^88^

**Tabela 14:** Recomendações para pacientes em uso de fármacos com potencial de prolongamento de QT.

Evitar uso de drogas relacionadas ao prolongamento de QTc em pacientes com QTc pré-tratamento > 470ms
Descontinuar drogas relacionadas ao prolongamento de QTc se QTc > 500ms ou > 550ms se QRS alargado basal (>120ms secundário a marca-passo ou bloqueio de ramo)
Redução de dose ou descontinuação de drogas se QTc aumentar > 60ms do valor pré-tratamento
Manter concentração de eletrólitos (potássio, magnésio e cálcio séricos) dentro dos valores normais
Evitar interações medicamentosas
Em pacientes com lesão renal aguda ou doença renal crônica, ajustar para a função renal as drogas com eliminação renal que prolonguem QTc
Evitar infusão intravenosa rápida de drogas que prolonguem QTc
Evitar administração em conjunto de mais de 1 droga que prolongue QTc
Evitar drogas que prolonguem QTc em pacientes com história de *torsade de points* induzida por drogas ou pacientes ressuscitados de morte cardíaca súbita
Evitar uso de drogas que prolonguem QTc em pacientes com doenças congênitas
Realizar ECG com frequência dependendo da terapia instituída, dose administrada e concentração das drogas

ECG: eletrocardiograma; QTc: intervalo QT corrigido. Fonte: Adaptado de Porta-Sanchez et al.^88^

O intervalo QT e os fatores de risco associados com prolongamento do QT devem ser avaliados antes e depois do tratamento com as drogas sabidamente relacionadas a arritmias cardíacas, como inibidores de tirosina quinase (crizotinibe, dasatinibe, sunitinibe) e trióxido de arsênio. A avaliação do ECG e dos eletrólitos deve ser feita durante o tratamento nos momentos basal, 7-15 dias após o início da terapêutica, após mudanças em doses nos primeiros 3 meses e a depender da frequência da terapia. Antes do adiamento da quimioterapia, deve-se considerar a suspensão de outros medicamentos relacionados ao prolongamento do QT, como antieméticos, antidepressivos, antiarrítmicos, antifúngicos, antipsicóticos. A correção de distúrbio hidroeletrolítico também deve ser realizada. Pacientes em uso de trióxido de arsênio devem ser monitorados com ECG semanalmente.^22^

O prolongamento do intervalo QT aumenta a incidência de arritmias ventriculares e *torsades de points.*^88^ As taquicardias ventriculares, em geral, estão associadas a miocardiopatias estruturais [doença arterial coronariana (DAC), miocardiopatia dilatada, cardiopatias do ventrículo direito, anormalidades congênitas, miocardiopatia hipertrófica e canalopatias].^89^ O objetivo do tratamento das arritmias ventriculares é diminuir morbidade e eventos de morte súbita, sendo fundamental avaliar os fatores desencadeantes. Indica-se terapêutica farmacológica para casos refratários e/ou sintomáticos.^90^

### 9.2. Fibrilação Atrial

A fibrilação atrial é a arritmia cardíaca mais frequente no paciente oncológico. Sua ocorrência está relacionada ao estado pró-inflamatório desses pacientes e também à resposta inflamatória à cirurgia oncológica e aos efeitos cardiotóxicos da terapia antineoplásica.^91^ A compreensão dos mecanismos que desencadeiam fibrilação atrial e promovem sua manutenção é importante para que haja prevenção.

Diversos mecanismos podem induzir fibrilação atrial, tais como alteração miocárdica por distúrbio hidroeletrolítico, dano lipossomal e mitocondrial, inflamação, doença pericárdica e aumento do estresse oxidativo induzindo apoptose celular.^92^

Existe grande dificuldade em se estabelecer a relação causal dos eventos arrítmicos com cada quimioterápico. Primeiramente, em razão do pequeno número de estudos publicados e da administração simultânea de muitas drogas, que tornam difícil relacionar droga e efeito. Os quimioterápicos mais associados a arritmias são as antraciclinas (doxorrubicina, epirrubicina), os agentes antimicrotúbulos (paclitaxel e docetaxel), os antimetabólitos (5-fluorouracil, capecitabina e gencitabina), os agentes alquilantes (cisplatina e ciclofosfamida), os inibidores da tirosina quinase (ibrutinibe, ponatinibe, sorafenibe e sunitinibe) e os anticorpos monoclonais (trastuzumabe e cetuximabe), além dos imunoterápicos.

O câncer é associado a um estado pró-trombótico, podendo aumentar o risco de eventos embólicos no paciente com fibrilação atrial, que também apresenta maior ocorrência de complicações hemorrágicas decorrentes do tratamento e, portanto, maior morbimortalidade. Não há recomendações baseadas em consensos e diretrizes sobre o uso dos antitrombóticos em pacientes com fibrilação atrial.^85^^,^^93^

A escolha da terapia antritrombótica no paciente com câncer deve ser individualizada, analisando-se os fatores farmacocinéticos e farmacodinâmicos, as interações medicamentosas, o risco de trombose e o risco de sangramento (IIa, B).

Deve-se evitar a varfarina no paciente oncológico com fibrilação atrial, pois essa droga é associada a menor eficácia e a maior risco de sangramento por interações medicamentosas, pela presença de maior ocorrência de disfunção hepática, alterações dietéticas, caquexia e desnutrição (IIa, B).

Os DOACs (dabigatrana, rivaroxabana, apixabana e edoxabana) são superiores à varfarina em termos de eficácia e sangramento na população geral com fibrilação atrial, porém a evidência para sua utilização no paciente com câncer vem de análises de subestudos e de dados observacionais (IIa, B).

Apesar de não haver validação dos escores clássicos na população com câncer, recomenda-se iniciar anticoagulação nesses pacientes utilizando-se os mesmos critérios adotados na população sem câncer: escores CHADS_2_ e CHA_2_DS_2_-VASc acima de 2 indicam anticoagulação (IIa, C) (Tabela 15).

**Tabela 15:** Escore de risco de tromboembolismo associado a fibrilação atrial

CHA_2_DS_2_-VASc		
	Descrição	Pontos
C	Insuficiência cardíaca	1
H	Hipertensão arterial	1
A_2_	Idade (≥ 75 anos)	2
D	Diabetes mellitus	1
S_2_	AIT ou AVC prévio	2
V	Doença vascular (IAM prévio, doença arterial periférica ou placa aórtica)	1
A	Idade (65-74 anos)	1
Sc	Sexo feminino	1

*AIT: ataque isquêmico transitório; AVC: acidente vascular cerebral; IAM: infarto agudo do miocárdio.*

A terapia antitrombótica no paciente com câncer deve ser personalizada, analisando-se perfil do paciente, tipo de câncer, risco de trombose e de sangramento, esse último por exemplo, por meio do escore de HAS-BLED (IIa, C) (Tabela 16).

**Tabela 16:** Escore de risco de sangramento associado a anticoagulação (HAS-BLED). Um ponto para cada critério presente

□	Hipertensão arterial (1 ponto)
□	Função hepática anormal (1 ponto)
□	Função renal anormal (1 ponto)
□	Acidente vascular cerebral (1 ponto)
□	Tendência ou predisposição a sangramento (1 ponto)
□	INR lábil em pacientes que tomam varfarina (1 ponto)
□	Idosos: acima de 60 anos (1 ponto)
□	Medicamentos: agente(s) antiplaquetários concomitantes ou AINES (1 ponto)
□	Abuso de álcool (1 ponto)
*AINES: anti-inflamatórios não esteroidais; INR: razão normalizada internacional*.	
0 ponto: 1,02 sangramento por 100 pacientes/ano	
1 ponto: 1,13 sangramento por 100 pacientes/ano	
2 pontos: 1,88 sangramento por 100 pacientes/ano	
3 pontos: 3,74 sangramentos por 100 pacientes/ano	
4 pontos: 8,70 sangramentos por 100 pacientes/ano	
5-9 pontos: dados insuficientes (alto risco)	

Não há estudos prospectivos e randomizados sobre DOACs em pacientes com câncer e fibrilação atrial. Análise de subestudos dos ensaios clínicos randomizados demonstra segurança e eficácia daquelas drogas nos pacientes com câncer. Essa evidência associada ao resultado de estudos em TEV e câncer, que confirmam a superioridade dos DOACs quando comparados a heparina de baixo peso molecular, sugere que os DOACs são opção viável como terapia antitrombótica nos pacientes com câncer e fibrilação atrial (IIa, C).

Uso rotineiro de DOACs em pacientes com fibrilação atrial e tumores do trato gastrointestinal e geniturinário pode ser considerado, devendo ser analisado risco potencial de sangramento (IIb,B).

Recomenda-se o envolvimento do cardiologista desde o início no manejo do paciente com câncer e fibrilação atrial, devido às maiores taxas de uso de anticoagulação e menor incidência de complicações isquêmicas e hemorrágicas (IIa, C).

## 10. Doença Arterial Coronariana

**Figura 9:**
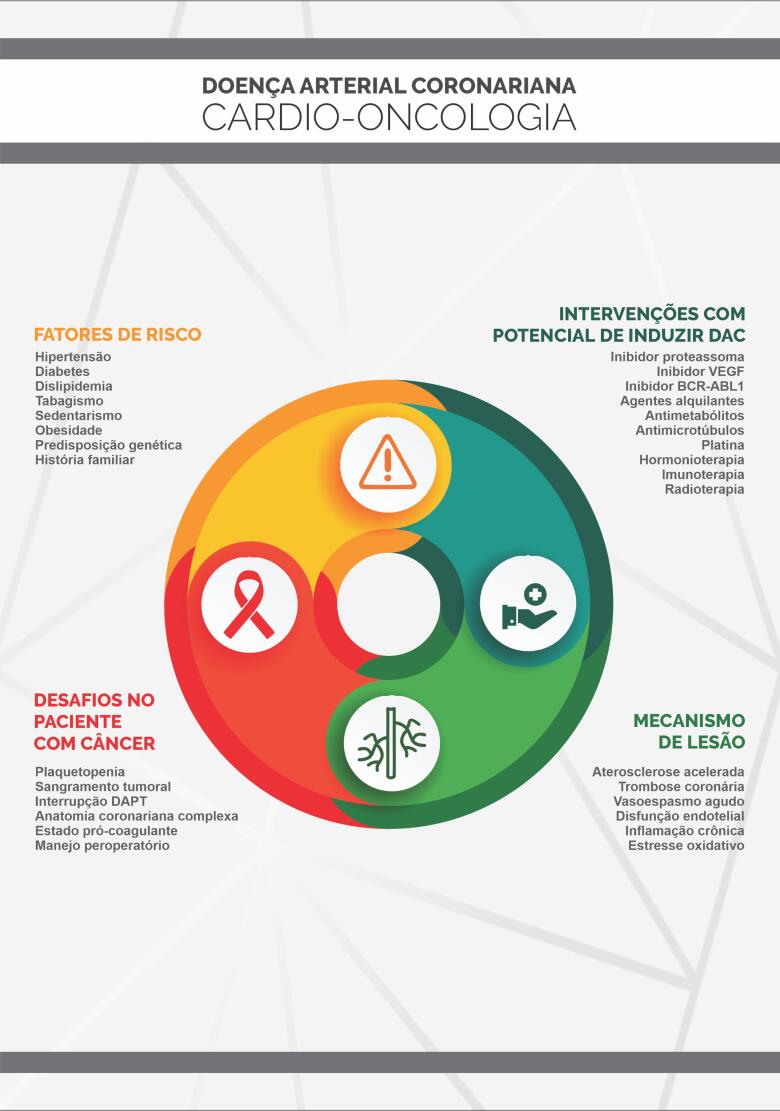
Doença arterial coronariana na cardio-oncologia. DAC: doença arterial coronariana; DAPT: dupla antiagregação plaquetária; VEGF: fator de crescimento endotelial vascular.

A DAC e o câncer apresentam diversos fatores de risco em comum e frequentemente coexistem no mesmo paciente (Figura 9). A presença de fatores de risco, como idade avançada, tabagismo, diabetes, HAS, sedentarismo e dislipidemia, é elevada nos pacientes com câncer.^94^ Outros fatores comuns nesses pacientes e que contribuem para o desenvolvimento de DAC são disfunção endotelial, estresse oxidativo, predisposição genética e inflamação crônica.^95^

O tratamento oncológico também contribui para a elevada prevalência de DAC nos pacientes oncológicos.^49^ Pacientes com câncer de pulmão submetidos a tratamento quimioterápico apresentam aumento de 5,3 vezes (IC 95%: 2,002-14,152) no risco de lesão coronariana importante, o que sugere que esse tratamento possa estar associado com complexidade anatômica.^96^ Os mecanismos principais de acometimento de DAC relacionada a terapia oncológica são: vasoespasmo, trombose e aterosclerose acelerada.^49^

Nos pacientes em uso de cisplatina de modo isolado ou em associação com vincristina ou bleomicina, observou-se trombose coronária em angiografia coronária sem aterosclerose prévia. Essas drogas podem induzir disfunção/lesão endotelial, que parece ser o mecanismo básico da alteração vasoativa causada por esses fármacos.^97^^–^^99^ A cisplatina leva à morte de células endoteliais pela produção de micropartículas pró-coagulantes.^100^

Outra classe de quimioterápico tipicamente relacionada a DAC nos pacientes com câncer são os antimetabólitos, especialmente o 5-fluorouracil e a capecitabina. A incidência de angina ou quadros agudos varia de 3,9% a 12,5%.^101^ O mecanismo pelo qual esses fármacos causam toxicidade não está completamente estabelecido e várias hipóteses foram geradas para explicar esses quadros, tais como vasoespasmo agudo, toxicidade direta aos miócitos, disfunção endotelial e estado de hipercoagulabilidade causando trombose.^101^^,^^102^ O vasoespasmo agudo é frequentemente observado e estudos experimentais sugerem que a vasoconstricção causada pelo 5-fluorouracil esteja relacionada a proteína quinase C e endotelina-1.^103^^,^^104^ De modo semelhante ao 5-fluorouracil, os taxanos são outra classe de fármacos que induzem quadros de angina secundários a espasmos coronarianos. A incidência de dor torácica relatada em pacientes em uso de paclitaxel é de aproximadamente 0,2-4%.^105^^,^^106^

Aterosclerose acelerada tem sido observada em pacientes recebendo tratamento com os inibidores de tirosina quinase de segunda e terceira gerações. Esses fármacos apresentam risco aumentado de oclusão coronária quando comparados a imatinibe (OR = 3,45 [IC 95%: 2,30-5,18).^107^ Dos inibidores de tirosina quinase, o ponatinibe parece ser o mais relacionado a toxicidade vascular. Tromboses arterial e venosa foram descritas em 27% dos pacientes recebendo ponatinibe, independentemente da presença de fatores de risco cardiovasculares.^107^ Os pacientes tratados com bevacizumabe apresentam elevado risco de isquemia coronariana quando comparados a grupo controle (RR: 2,47; IC 95%: 1,4-4,36).^108^ Os pacientes em uso de ICIs apresentam uma composição de células inflamatórias alterada na placa aterosclerótica (aumento de células TCD3+ em relação a macrófagos CD68+), que pode predispor a progressão de placa e/ou eventos coronários clínicos.^109^

O tratamento radioterápico é classicamente relacionado ao desenvolvimento de DAC, em geral reportado de modo tardio após exposição à radiação. A incidência é variável na literatura, com tendência à redução nas últimas décadas em virtude de técnicas mais modernas que diminuem a radiação direta ao coração. A patogênese da DAC é multifatorial, resultando em lesão miocárdica direta, alteração do tônus vascular, ativação inflamatória e estresse oxidativo.^110^^,^^111^

Em virtude da complexidade do paciente com câncer e DAC, sua mortalidade é maior do que a de pacientes sem câncer a longo prazo.^112^^,^^113^ Na abordagem desses pacientes, é importante conhecer o prognóstico oncológico, as perspectivas terapêuticas e a programação de cirurgias oncológicas. O controle de fatores de risco deve ser reforçado nos pacientes com DAC evidenciada. O uso de *stent* farmacológico deve ser preferido nesses pacientes quando indicada intervenção terapêutica.^114^ A terapia com antiplaquetários deve ser mantida conforme diretrizes habituais de abordagem de DAC e síndrome coronariana aguda, a menos que existam contraindicações, como sangramento tumoral.^115^^–^^117^

Recomendações:

- O controle de fatores de risco (HAS, diabetes, dislipidemias) e da perda ponderal, a cessação de tabagismo e a orientação dietética devem ser feitos em todos os pacientes que serão submetidos a fármacos com predisposição a DAC (I, A).
- O *stent* farmacológico deve ser preferido nesses pacientes (IIa, B).
- A dupla antiagregação pode ser mantida em pacientes com níveis de plaquetas > 30 mil, na ausência de contraindicação (IIa, B).
- A pesquisa de DAC está indicada em pacientes após 5 anos da exposição mediastinal a dose de 30 gy ou mais (IIa, B).
- Nos pacientes que apresentaram síndrome coronariana aguda com 5-fluorouracil, recomenda-se a avaliação do cardio-oncologista e deve-se considerar a estratificação de DAC (IIa, B).
- Pode-se considerar a re-exposição em pacientes que apresentaram eventos leves, que estejam assintomáticos e cujo benefício do 5-fluorouracil tenha impacto prognóstico. O uso de nitratos e vasodilatadores pode ser considerado nesse cenário (IIb, B).

## 11. Hipertensão Arterial

A prevalência de HAS é maior nos pacientes com câncer e sobreviventes quando comparados à população geral.^118^ A HAS é o principal fator de risco modificável de eventos cardiovasculares nesses pacientes.^119^ A HAS, a doença renal crônica, a doença cardiovascular e o câncer têm fatores de risco em comum, como tabagismo, obesidade e diabetes. Muitos tipos de câncer e seus tratamentos causam ou agravam a HAS preexistente por efeitos vasculares, endoteliais e renais.^22^

Recomenda-se a aferição periódica da pressão arterial no paciente com câncer (IIa, C).

A seleção de agentes anti-hipertensivos deve levar em consideração fatores de risco individuais, efeitos do tratamento antineoplásico e interações medicamentosas. Estima-se que 35% dos pacientes com câncer desenvolverão HAS ao longo do tratamento. Pacientes com história de câncer têm uma prevalência maior de HAS que a população geral.^94^ Pacientes com câncer renal, gástrico e ovariano têm níveis tensionais mais elevados que aqueles com outros sítios tumorais. Exposição a quimioterapia é um fator de risco independente para HAS.^120^

### 11.1. HAS e Tratamento Quimioterápico

A terapia com inibidores de tirosina quinase anti-VEGF e inibidores de tirosina quinase multi-alvo acentua e induz HAS.^121^ Os mecanismos são redução da produção de óxido nítrico e de angiogênese, levando a aumento da resistência vascular sistêmica. A terapia anti-VEGF leva à retenção fluida por comprometer a natriurese, além de induzir vasoconstricção mediada por endotelina-1 e microangiopatia trombótica, semelhante à fisiopatologia da eclampsia.^121^ Em recente meta-análise publicada, o uso dos inibidores de tirosina quinase anti-VEGF aumentou o risco de cardiotoxicidade, como HAS, sangramento e disfunção ventricular. A HAS foi a cardiotoxicidade vascular mais comum.^122^

Os agentes alquilantes parecem induzir HAS por nefrotoxicidade, porém não há muita evidência de seu real efeito na pressão arterial. A ciclofosfamida tem sido associada a complicações vasculares múltiplas, como doença veno-oclusiva no pulmão e fígado, doença tromboembólica e isquemia miocárdica. A evidência pré-clínica demonstra injúria endotelial e anormalidades do sistema renina-angiotensina-aldosterona nos animais tratados com ciclofosfamida.^21^^,^^22^

Tanto a ifosfamida quanto a cisplatina aparentemente induzem HAS por causar nefrotoxicidade.^123^ Os agentes antimicrotúbulos afetam a mitose, agindo na tubulina para impedir a polimerização do microtúbulo. Estudos experimentais mostram que a vimblastina atua no endotélio e na apoptose, porém seu efeito na HAS é desconhecido.^124^^,^^125^ A gencitabina e os inibidores de proteassoma podem desencadear HAS associada a microangiopatia trombótica. As medicações adjuvantes utilizadas nos pacientes com câncer associadas a HAS são: corticosteroides, eritropoietina, inibidores de calcineurina e anti-inflamatórios.

### 11.2. HAS Induzida pelo Câncer

A HAS pode ser uma manifestação paraneoplásica do carcinoma hepatocelular, câncer renal, doença carcinoide, entre outras. Ocorre por produção de renina, angiotensinogênio, angiotensina I ou catecolaminas. Entre indivíduos com carcinoma de células renais, a prevalência de HAS excede 75% e ocorre pela perda de néfrons relacionada a nefrectomia e particularmente pelo tratamento com inibidores VEGF. Além disso, o carcinoma renal pode secretar peptídeos vasoativos, principalmente a endotelina-1. A presença de HAS no câncer renal pode significar doença mais agressiva, com impacto negativo no prognóstico.^126^^,^^127^ O feocromocitoma e o paraganglioma são tumores neuroendócrinos das células cromafins, com incidência anual de 0,8 por 100 mil pessoas. A HAS relacionada a esses tumores é causada por secreção de catecolaminas (norepinefrina, epinefrina e dopamina) e pode estar associada a sintomas como cefaleia, palpitações e sudorese.^128^

O paciente com câncer e HAS têm maior incidência de insuficiências cardíaca e renal. A HAS é fator de risco independente de DAC, insuficiência cardíaca e arritmia, sendo o principal fator de risco modificável para evitar insuficiência cardíaca.^47^^,^^129^

A pressão arterial deve ser aferida de maneira adequada e periódica no paciente com câncer (I, A).

Pacientes em uso de inibidores de tirosina quinase anti-VEGF, inibidores de tirosina quinase multi-alvo, agentes alquilantes ou esteroides em altas doses devem ser submetidos a monitorização mais frequente da pressão arterial (IIa, C).

A meta da pressão arterial no paciente com câncer segue as recomendações do paciente sem câncer: < 130 x 80 mmHg (IIa, B).

O tratamento medicamentoso deve ser personalizado, porém a demonstração de proteinúria ou de disfunção ventricular determina a indicação de IECA ou AT1 (IIa, B).

No caso de não haver proteinúria ou disfunção ventricular, bloqueador de canal de cálcio do tipo diidropiridina (amlodipina) pode ser iniciado (IIa, C).

Diuréticos devem ser utilizados com critério bem definido e vigilância pelo risco de hipovolemia e distúrbios hidroeletrolíticos (IIa, C).

Bloqueador de canal de cálcio não diidropiridínico (verapamil e diltiazem) em pacientes com câncer (III, B). Esses medicamentos, por serem metabolizados no CYP3A4, podem alterar os níveis séricos dos quimioterápicos.

Sugere-se buscar causas secundárias de HAS, como hipovolemia e dor (IIa, C).
