# Supplementary material for: Diretriz Brasileira de Cardio-oncologia – 2020
Source: Arq Bras Cardiol. 2020 Nov 1;115(5):1006–43. [Article in Portuguese] doi: 10.36660/abc.20201006 (PMC8452206; doi:10.36660/abc.20201006)
Supplement: Translation

# Translation: Brazilian Cardio-oncology Guideline – 2020

**Authors:** Ludhmila Abrahão Hajjar, Isabela Bispo Santos da Silva da Costa, Marcelo Antônio Cartaxo Queiroga Lopes, Paulo Marcelo Gehm Hoff, Maria Del Pilar Estevez Diz, Silvia Moulin Ribeiro Fonseca, Cristina Salvadori Bittar, Marília Harumi Higuchi dos Santos Rehder, Stephanie Itala Rizk, Dirceu Rodrigues Almeida, Gustavo dos Santos Fernandes, Luís Beck-da-Silva, Carlos Augusto Homem de Magalhães Campos, Marcelo Westerlund Montera, Sílvia Marinho Martins Alves, Júlia Tizue Fukushima, Maria Verônica Câmara dos Santos, Carlos Eduardo Negrão, Thiago Liguori Feliciano da Silva, Silvia Moreira Ayub Ferreira, Marcus Vinicius Bolivar Malachias, Maria da Consolação Vieira Moreira, Manuel Maria Ramos Valente, Veronica Cristina Quiroga Fonseca, Maria Carolina Feres de Almeida Soeiro, Juliana Barbosa Sobral Alves, Carolina Maria Pinto Domingues Carvalho Silva, João Sbano, Ricardo Pavanello, Ibraim Masciarelli F. Pinto, Antônio Felipe Simão, Marianna Deway Andrade Dracoulakis, Ana Oliveira Hoff, Bruna Morhy Borges Leal Assunção, Yana Novis, Laura Testa, Aristóteles Comte de Alencar, Cecília Beatriz Bittencourt Viana Cruz, Juliana Pereira, Diego Ribeiro Garcia, Cesar Higa Nomura, Carlos Eduardo Rochitte, Ariane Vieira Scarlatelli Macedo, Patricia Tavares Felipe Marcatti, Wilson Mathias, Evanius Garcia Wiermann, Renata do Val, Helano Freitas, Anelisa Coutinho, Clarissa Maria de Cerqueira Mathias, Fernando Meton de Alencar Camara Vieira, André Deeke Sasse, Vanderson Rocha, José Antônio Franchini Ramires, Roberto Kalil

**Affiliations:** 1 São PauloSP Brazil Instituto do Coração do Hospital das Clínicas da Faculdade de Medicina da Universidade de São Paulo, São Paulo, SP – Brazil; 2 São PauloSP Brazil Instituto do Câncer Hospital das Clínicas da Faculdade de Medicina da Universidade de São Paulo, São Paulo, SP – Brazil; 3 João PessoaPB Brazil Hospital Alberto Urquiza Wanderley, João Pessoa, PB – Brazil; 4 Rio de JaneiroRJ Brazil Instituto D'or Pesquisa e Ensino, Rio de Janeiro, RJ – Brazil; 5 São PauloSP Brazil Hospital Sírio Libanês, São Paulo, SP – Brazil; 6 São PauloSP Brazil Universidade Federal de São Paulo (UNIFESP), São Paulo, SP – Brazil; 7 Porto AlegreRS Brazil Hospital de Clínicas de Porto Alegre, Porto Alegre, RS – Brazil; 8 Porto AlegreRS Brazil Universidade Federal do Rio Grande do Sul, Porto Alegre, RS – Brazil; 9 Rio de JaneiroRJ Brazil Hospital Pró-Cardíaco, Rio de Janeiro, RJ – Brazil; 10 RecifePE Brazil Pronto Socorro Cardiológico de Pernambuco (PROCAPE), Recife, PE – Brazil; 11 São PauloSP Brazil Sociedade Brasileira de Oncologia Pediátrica (SOBOPE), São Paulo, SP – Brazil; 12 Rio de JaneiroRJ Brazil Departamento de Cardiopatias Congênitas e Cardiologia Pediátrica (DCC/CP) da Sociedade Brasileira de Cardiologia (SBC), Rio de Janeiro, RJ – Brazil; 13 Belo HorizonteMG Brazil Faculdade de Ciências Médicas de Minas Gerais, Belo Horizonte, MG – Brazil; 14 Belo HorizonteMG Brazil Universidade Federal de Minas Gerais, Belo Horizonte, MG – Brazil; 15 São PauloSP Brazil Hospital do Coração da Associação do Sanatório Sírio – HCor, São Paulo, SP – Brazil; 16 São PauloSP Brazil Instituto Dante Pazzanese de Cardiologia, São Paulo, SP – Brazil; 17 São JoséSC Brazil Instituto de Cardiologia de Santa Catarina, São José, SC – Brazil; 18 SalvadorBA Brazil Hospital da Bahia, Salvador, BA – Brazil; 19 ManausAM Brazil Universidade Federal do Amazonas, Manaus, AM – Brazil; 20 São PauloSP Brazil Hospital do Coração (HCOR), São Paulo, SP – Brazil; 21 São PauloSP Brazil Santa Casa de Misericórdia de São Paulo, São Paulo, SP – Brazil; 22 Belo HorizonteMG Brazil Rede Mater Dei de Saúde, Belo Horizonte, MG – Brazil; 23 CuritibaPR Brazil Centro de Oncologia do Paraná, Curitiba, PR – Brazil; 24 São PauloSP Brazil A.C. Camargo Cancer Center, São Paulo, SP – Brazil; 25 SalvadorBA Brazil Hospital Aliança, Salvador, BA – Brazil; 26 SalvadorBA Brazil NOB/Oncoclínicas, Salvador, BA – Brazil; 27 São PauloSP Brazil Sociedade Brasileira de Oncologia Clínica, São Paulo, SP – Brazil; 28 Rio de JaneiroRJ Brazil Grupo Americas Oncologia, Rio de Janeiro, RJ – Brazil; 29 CampinasSP Brazil Grupo SOnhe, Campinas, SP – Brazil; 30 CampinasSP Brazil Universidade Estadual de Campinas (Unicamp), Campinas, SP – Brazil

**Table:** 

Brazilian Cardio-oncology Guideline – 2020	
The report below lists declarations of interest as reported to the SBC by the experts during the period of the development of these update, 2020.	
Expert	Type of relationship with industry
Ana Oliveira Hoff	FINANCIAL DECLARATION A - ECONOMICALLY RELEVANT PAYMENTS OF ANY KIND MADE TO (i) YOU, (ii) YOUR SPOUSE/PARTNER OR ANY OTHER PERSON LIVING WITH YOU, (iii) ANY LEGAL PERSON IN WHICH ANY OF THESE IS EITHER A DIRECT OR INDIRECT CONTROLLING OWNER, BUSINESS PARTNER, SHAREHOLDER OR PARTICIPANT; ANY PAYMENTS RECEIVED FOR LECTURES, LESSONS, TRAINING INSTRUCTION, COMPENSATION, FEES PAID FOR PARTICIPATION IN ADVISORY BOARDS, INVESTIGATIVE BOARDS OR OTHER COMMITTEES, ETC. FROM THE BRAZILIAN OR INTERNATIONAL PHARMACEUTICAL, ORTHOSIS, PROSTHESIS, EQUIPMENT AND IMPLANTS INDUSTRY: - Bayer: thyroid cancer - Exelixis: thyroid cancer - Eli Lilly: thyroid cancer - United B - RESEARCH FUNDING UNDER YOUR DIRECT/PERSONAL RESPONSIBILITY (DIRECTED TO THE DEPARTMENT OR INSTITUTION) FROM THE BRAZILIAN OR INTERNATIONAL PHARMACEUTICAL, ORTHOSIS, PROSTHESIS, EQUIPMENT AND IMPLANTS INDUSTRY: - Exelixis: thyroid cancer - Eli Lilly: thyroid cancer
André Deeke Sasse	FINANCIAL DECLARATION A - ECONOMICALLY RELEVANT PAYMENTS OF ANY KIND MADE TO (i) YOU, (ii) YOUR SPOUSE/PARTNER OR ANY OTHER PERSON LIVING WITH YOU, (iii) ANY LEGAL PERSON IN WHICH ANY OF THESE IS EITHER A DIRECT OR INDIRECT CONTROLLING OWNER, BUSINESS PARTNER, SHAREHOLDER OR PARTICIPANT; ANY PAYMENTS RECEIVED FOR LECTURES, LESSONS, TRAINING INSTRUCTION, COMPENSATION, FEES PAID FOR PARTICIPATION IN ADVISORY BOARDS, INVESTIGATIVE BOARDS OR OTHER COMMITTEES, ETC. FROM THE BRAZILIAN OR INTERNATIONAL PHARMACEUTICAL, ORTHOSIS, PROSTHESIS, EQUIPMENT AND IMPLANTS INDUSTRY: - Astellas: oncology - Bayer: oncology - Janssen: oncology - Merck Serono - MSD - Novartis - Roche OTHER RELATIONSHIPS FUNDING OF CONTINUING MEDICAL EDUCATION ACTIVITIES, INCLUDING TRAVEL, ACCOMMODATION AND REGISTRATION IN CONFERENCES AND COURSES, FROM THE BRAZILIAN OR INTERNATIONAL PHARMACEUTICAL, ORTHOSIS, PROSTHESIS, EQUIPMENT AND IMPLANTS INDUSTRY: - MSD: oncology - Janssen: oncology - Novartis: oncology
Anelisa Coutinho	FINANCIAL DECLARATION A - ECONOMICALLY RELEVANT PAYMENTS OF ANY KIND MADE TO (i) YOU, (ii) YOUR SPOUSE/PARTNER OR ANY OTHER PERSON LIVING WITH YOU, (iii) ANY LEGAL PERSON IN WHICH ANY OF THESE IS EITHER A DIRECT OR INDIRECT CONTROLLING OWNER, BUSINESS PARTNER, SHAREHOLDER OR PARTICIPANT; ANY PAYMENTS RECEIVED FOR LECTURES, LESSONS, TRAINING INSTRUCTION, COMPENSATION, FEES PAID FOR PARTICIPATION IN ADVISORY BOARDS, INVESTIGATIVE BOARDS OR OTHER COMMITTEES, ETC. FROM THE BRAZILIAN OR INTERNATIONAL PHARMACEUTICAL, ORTHOSIS, PROSTHESIS, EQUIPMENT AND IMPLANTS INDUSTRY: - Bayer: oncology - Amgen: oncology - Roche: oncology - Merck group - MSD - Lilly - Servier B - RESEARCH FUNDING UNDER YOUR DIRECT/PERSONAL RESPONSIBILITY (DIRECTED TO THE DEPARTMENT OR INSTITUTION) FROM THE BRAZILIAN OR INTERNATIONAL PHARMACEUTICAL, ORTHOSIS, PROSTHESIS, EQUIPMENT AND IMPLANTS INDUSTRY: - Bristol: oncology - Servier: oncology OTHER RELATIONSHIPS FUNDING OF CONTINUING MEDICAL EDUCATION ACTIVITIES, INCLUDING TRAVEL, ACCOMMODATION AND REGISTRATION IN CONFERENCES AND COURSES, FROM THE BRAZILIAN OR INTERNATIONAL PHARMACEUTICAL, ORTHOSIS, PROSTHESIS, EQUIPMENT AND IMPLANTS INDUSTRY: - Roche: oncology - Servier: oncology - Bayer: oncology - Merck group - Amgen - Sanofi
Antônio Felipe Simão	FINANCIAL DECLARATION A - ECONOMICALLY RELEVANT PAYMENTS OF ANY KIND MADE TO (i) YOU, (ii) YOUR SPOUSE/PARTNER OR ANY OTHER PERSON LIVING WITH YOU, (iii) ANY LEGAL PERSON IN WHICH ANY OF THESE IS EITHER A DIRECT OR INDIRECT CONTROLLING OWNER, BUSINESS PARTNER, SHAREHOLDER OR PARTICIPANT; ANY PAYMENTS RECEIVED FOR LECTURES, LESSONS, TRAINING INSTRUCTION, COMPENSATION, FEES PAID FOR PARTICIPATION IN ADVISORY BOARDS, INVESTIGATIVE BOARDS OR OTHER COMMITTEES, ETC. FROM THE BRAZILIAN OR INTERNATIONAL PHARMACEUTICAL, ORTHOSIS, PROSTHESIS, EQUIPMENT AND IMPLANTS INDUSTRY: - AstraZeneca: cardiology OTHER RELATIONSHIPS FUNDING OF CONTINUING MEDICAL EDUCATION ACTIVITIES, INCLUDING TRAVEL, ACCOMMODATION AND REGISTRATION IN CONFERENCES AND COURSES, FROM THE BRAZILIAN OR INTERNATIONAL PHARMACEUTICAL, ORTHOSIS, PROSTHESIS, EQUIPMENT AND IMPLANTS INDUSTRY: - AstraZeneca - Bayer
Ariane Vieira Scarlatelli Macedo	FINANCIAL DECLARATION A - ECONOMICALLY RELEVANT PAYMENTS OF ANY KIND MADE TO (i) YOU, (ii) YOUR SPOUSE/PARTNER OR ANY OTHER PERSON LIVING WITH YOU, (iii) ANY LEGAL PERSON IN WHICH ANY OF THESE IS EITHER A DIRECT OR INDIRECT CONTROLLING OWNER, BUSINESS PARTNER, SHAREHOLDER OR PARTICIPANT; ANY PAYMENTS RECEIVED FOR LECTURES, LESSONS, TRAINING INSTRUCTION, COMPENSATION, FEES PAID FOR PARTICIPATION IN ADVISORY BOARDS, INVESTIGATIVE BOARDS OR OTHER COMMITTEES, ETC. FROM THE BRAZILIAN OR INTERNATIONAL PHARMACEUTICAL, ORTHOSIS, PROSTHESIS, EQUIPMENT AND IMPLANTS INDUSTRY: - Bayer: anticoagulants - Pfizer: anticoagulants - Daichii Sankyo: anticoagulants - AstraZeneca: anticoagulants OTHER RELATIONSHIPS FUNDING OF CONTINUING MEDICAL EDUCATION ACTIVITIES, INCLUDING TRAVEL, ACCOMMODATION AND REGISTRATION IN CONFERENCES AND COURSES, FROM THE BRAZILIAN OR INTERNATIONAL PHARMACEUTICAL, ORTHOSIS, PROSTHESIS, EQUIPMENT AND IMPLANTS INDUSTRY: - Bayer: anticoagulants - Pfizer: anticoagulants - Zodiac: chemotherapy - Ferring
Aristóteles Comte de Alencar Filho	FINANCIAL DECLARATION A - ECONOMICALLY RELEVANT PAYMENTS OF ANY KIND MADE TO (i) YOU, (ii) YOUR SPOUSE/PARTNER OR ANY OTHER PERSON LIVING WITH YOU, (iii) ANY LEGAL PERSON IN WHICH ANY OF THESE IS EITHER A DIRECT OR INDIRECT CONTROLLING OWNER, BUSINESS PARTNER, SHAREHOLDER OR PARTICIPANT; ANY PAYMENTS RECEIVED FOR LECTURES, LESSONS, TRAINING INSTRUCTION, COMPENSATION, FEES PAID FOR PARTICIPATION IN ADVISORY BOARDS, INVESTIGATIVE BOARDS OR OTHER COMMITTEES, ETC. FROM THE BRAZILIAN OR INTERNATIONAL PHARMACEUTICAL, ORTHOSIS, PROSTHESIS, EQUIPMENT AND IMPLANTS INDUSTRY: - Novartis: Entresto - Sandoz: Pidezot
Bruna Morhy Borges Leal Assunção	Nothing to be declared
Carlos Augusto Homem de Magalhães Campos	Nothing to be declared
Carlos Eduardo Negrão	Nothing to be declared
Carlos Eduardo Rochitte	Nothing to be declared
Carolina Maria Pinto Domingues Carvalho Silva	Nothing to be declared
Cecilia Beatriz Bittencourt Viana Cruz	Nothing to be declared
Cesar Higa Nomura	Nothing to be declared
Clarissa Maria de Cerqueira Mathias	Nothing to be declared
Cristina Salvadori Bittar	Nothing to be declared
Diego Ribeiro Garcia	Nothing to be declared
Dirceu Rodrigues Almeida	Nothing to be declared
Evanius Garcia Wiermann	FINANCIAL DECLARATION A - ECONOMICALLY RELEVANT PAYMENTS OF ANY KIND MADE TO (i) YOU, (ii) YOUR SPOUSE/PARTNER OR ANY OTHER PERSON LIVING WITH YOU, (iii) ANY LEGAL PERSON IN WHICH ANY OF THESE IS EITHER A DIRECT OR INDIRECT CONTROLLING OWNER, BUSINESS PARTNER, SHAREHOLDER OR PARTICIPANT; ANY PAYMENTS RECEIVED FOR LECTURES, LESSONS, TRAINING INSTRUCTION, COMPENSATION, FEES PAID FOR PARTICIPATION IN ADVISORY BOARDS, INVESTIGATIVE BOARDS OR OTHER COMMITTEES, ETC. FROM THE BRAZILIAN OR INTERNATIONAL PHARMACEUTICAL, ORTHOSIS, PROSTHESIS, EQUIPMENT AND IMPLANTS INDUSTRY: - Sanofi: Xarelto - Novartis: Alpelisib OTHER RELATIONSHIPS FUNDING OF CONTINUING MEDICAL EDUCATION ACTIVITIES, INCLUDING TRAVEL, ACCOMMODATION AND REGISTRATION IN CONFERENCES AND COURSES, FROM THE BRAZILIAN OR INTERNATIONAL PHARMACEUTICAL, ORTHOSIS, PROSTHESIS, EQUIPMENT AND IMPLANTS INDUSTRY: - Janssen: Abiraterona - Bayer: Xofigo - Libbs: Zedora
Fernando Meton de Alencar Camara Vieira	FINANCIAL DECLARATION A - ECONOMICALLY RELEVANT PAYMENTS OF ANY KIND MADE TO (i) YOU, (ii) YOUR SPOUSE/PARTNER OR ANY OTHER PERSON LIVING WITH YOU, (iii) ANY LEGAL PERSON IN WHICH ANY OF THESE IS EITHER A DIRECT OR INDIRECT CONTROLLING OWNER, BUSINESS PARTNER, SHAREHOLDER OR PARTICIPANT; ANY PAYMENTS RECEIVED FOR LECTURES, LESSONS, TRAINING INSTRUCTION, COMPENSATION, FEES PAID FOR PARTICIPATION IN ADVISORY BOARDS, INVESTIGATIVE BOARDS OR OTHER COMMITTEES, ETC. FROM THE BRAZILIAN OR INTERNATIONAL PHARMACEUTICAL, ORTHOSIS, PROSTHESIS, EQUIPMENT AND IMPLANTS INDUSTRY: - BMS: colorectal cancer (clinical research)
Gustavo dos Santos Fernandes	FINANCIAL DECLARATION A - ECONOMICALLY RELEVANT PAYMENTS OF ANY KIND MADE TO (i) YOU, (ii) YOUR SPOUSE/PARTNER OR ANY OTHER PERSON LIVING WITH YOU, (iii) ANY LEGAL PERSON IN WHICH ANY OF THESE IS EITHER A DIRECT OR INDIRECT CONTROLLING OWNER, BUSINESS PARTNER, SHAREHOLDER OR PARTICIPANT; ANY PAYMENTS RECEIVED FOR LECTURES, LESSONS, TRAINING INSTRUCTION, COMPENSATION, FEES PAID FOR PARTICIPATION IN ADVISORY BOARDS, INVESTIGATIVE BOARDS OR OTHER COMMITTEES, ETC. FROM THE BRAZILIAN OR INTERNATIONAL PHARMACEUTICAL, ORTHOSIS, PROSTHESIS, EQUIPMENT AND IMPLANTS INDUSTRY: - Roche: Bevacizumab e Trastuzumab - MSD: immunotherapy - BMS: immunotherapy - Bayer - Sanofi - Novartis B - RESEARCH FUNDING UNDER YOUR DIRECT/PERSONAL RESPONSIBILITY (DIRECTED TO THE DEPARTMENT OR INSTITUTION) FROM THE BRAZILIAN OR INTERNATIONAL PHARMACEUTICAL, ORTHOSIS, PROSTHESIS, EQUIPMENT AND IMPLANTS INDUSTRY: - BMS: immunotherapy - MSD: immunotherapy - Roche: immunotherapy
Helano Freitas	Nothing to be declared
Ibraim Masciarelli F. Pinto	FINANCIAL DECLARATION A - ECONOMICALLY RELEVANT PAYMENTS OF ANY KIND MADE TO (i) YOU, (ii) YOUR SPOUSE/PARTNER OR ANY OTHER PERSON LIVING WITH YOU, (iii) ANY LEGAL PERSON IN WHICH ANY OF THESE IS EITHER A DIRECT OR INDIRECT CONTROLLING OWNER, BUSINESS PARTNER, SHAREHOLDER OR PARTICIPANT; ANY PAYMENTS RECEIVED FOR LECTURES, LESSONS, TRAINING INSTRUCTION, COMPENSATION, FEES PAID FOR PARTICIPATION IN ADVISORY BOARDS, INVESTIGATIVE BOARDS OR OTHER COMMITTEES, ETC. FROM THE BRAZILIAN OR INTERNATIONAL PHARMACEUTICAL, ORTHOSIS, PROSTHESIS, EQUIPMENT AND IMPLANTS INDUSTRY: - GE Healthcare: tomography - Novo Nordisk: farma
Isabela Bispo Santos da Silva da Costa	Nothing to be declared
João Cesar Nunes Sbano	Nothing to be declared
José Antônio Franchini Ramires	Nothing to be declared
Júlia Tizue Fukushima	Nothing to be declared
Juliana Barbosa Sobral Alves	FINANCIAL DECLARATION A - ECONOMICALLY RELEVANT PAYMENTS OF ANY KIND MADE TO (i) YOU, (ii) YOUR SPOUSE/PARTNER OR ANY OTHER PERSON LIVING WITH YOU, (iii) ANY LEGAL PERSON IN WHICH ANY OF THESE IS EITHER A DIRECT OR INDIRECT CONTROLLING OWNER, BUSINESS PARTNER, SHAREHOLDER OR PARTICIPANT; ANY PAYMENTS RECEIVED FOR LECTURES, LESSONS, TRAINING INSTRUCTION, COMPENSATION, FEES PAID FOR PARTICIPATION IN ADVISORY BOARDS, INVESTIGATIVE BOARDS OR OTHER COMMITTEES, ETC. FROM THE BRAZILIAN OR INTERNATIONAL PHARMACEUTICAL, ORTHOSIS, PROSTHESIS, EQUIPMENT AND IMPLANTS INDUSTRY: - Janssen: pulmonary hypertension - Bayer: hypertension
Juliana Pereira	Nothing to be declared
Laura Testa	FINANCIAL DECLARATION A - ECONOMICALLY RELEVANT PAYMENTS OF ANY KIND MADE TO (i) YOU, (ii) YOUR SPOUSE/PARTNER OR ANY OTHER PERSON LIVING WITH YOU, (iii) ANY LEGAL PERSON IN WHICH ANY OF THESE IS EITHER A DIRECT OR INDIRECT CONTROLLING OWNER, BUSINESS PARTNER, SHAREHOLDER OR PARTICIPANT; ANY PAYMENTS RECEIVED FOR LECTURES, LESSONS, TRAINING INSTRUCTION, COMPENSATION, FEES PAID FOR PARTICIPATION IN ADVISORY BOARDS, INVESTIGATIVE BOARDS OR OTHER COMMITTEES, ETC. FROM THE BRAZILIAN OR INTERNATIONAL PHARMACEUTICAL, ORTHOSIS, PROSTHESIS, EQUIPMENT AND IMPLANTS INDUSTRY: - Libbs: oncology - Novartis: oncology - Roche: oncology - Pfizer: oncology B - RESEARCH FUNDING UNDER YOUR DIRECT/PERSONAL RESPONSIBILITY (DIRECTED TO THE DEPARTMENT OR INSTITUTION) FROM THE BRAZILIAN OR INTERNATIONAL PHARMACEUTICAL, ORTHOSIS, PROSTHESIS, EQUIPMENT AND IMPLANTS INDUSTRY: - Roche: oncologia - institutional financing - Lilly: oncologia - institutional financing - Novartis: oncologia - institutional financing - MSD: oncologia - institutional financing OTHER RELATIONSHIPS FUNDING OF CONTINUING MEDICAL EDUCATION ACTIVITIES, INCLUDING TRAVEL, ACCOMMODATION AND REGISTRATION IN CONFERENCES AND COURSES, FROM THE BRAZILIAN OR INTERNATIONAL PHARMACEUTICAL, ORTHOSIS, PROSTHESIS, EQUIPMENT AND IMPLANTS INDUSTRY: - Pfizer: oncology - Libbs: oncology - United Medical: oncology
Ludhmila Abrahão Hajjar	Nothing to be declared
Luís Beck-da-Silva	FINANCIAL DECLARATION A - ECONOMICALLY RELEVANT PAYMENTS OF ANY KIND MADE TO (i) YOU, (ii) YOUR SPOUSE/PARTNER OR ANY OTHER PERSON LIVING WITH YOU, (iii) ANY LEGAL PERSON IN WHICH ANY OF THESE IS EITHER A DIRECT OR INDIRECT CONTROLLING OWNER, BUSINESS PARTNER, SHAREHOLDER OR PARTICIPANT; ANY PAYMENTS RECEIVED FOR LECTURES, LESSONS, TRAINING INSTRUCTION, COMPENSATION, FEES PAID FOR PARTICIPATION IN ADVISORY BOARDS, INVESTIGATIVE BOARDS OR OTHER COMMITTEES, ETC. FROM THE BRAZILIAN OR INTERNATIONAL PHARMACEUTICAL, ORTHOSIS, PROSTHESIS, EQUIPMENT AND IMPLANTS INDUSTRY: - Novartis: heart failure - Merck: heart failure B - RESEARCH FUNDING UNDER YOUR DIRECT/PERSONAL RESPONSIBILITY (DIRECTED TO THE DEPARTMENT OR INSTITUTION) FROM THE BRAZILIAN OR INTERNATIONAL PHARMACEUTICAL, ORTHOSIS, PROSTHESIS, EQUIPMENT AND IMPLANTS INDUSTRY: - AMGEN: heart failure - Novartis: heart failure
Manuel Maria Ramos Valente Neto	Nothing to be declared
Marcelo Antônio Cartaxo Queiroga Lopes	Nothing to be declared
Marcelo Westerlund Montera	Nothing to be declared
Marcus Vinicius Bolivar Malachias	FINANCIAL DECLARATION A - ECONOMICALLY RELEVANT PAYMENTS OF ANY KIND MADE TO (i) YOU, (ii) YOUR SPOUSE/PARTNER OR ANY OTHER PERSON LIVING WITH YOU, (iii) ANY LEGAL PERSON IN WHICH ANY OF THESE IS EITHER A DIRECT OR INDIRECT CONTROLLING OWNER, BUSINESS PARTNER, SHAREHOLDER OR PARTICIPANT; ANY PAYMENTS RECEIVED FOR LECTURES, LESSONS, TRAINING INSTRUCTION, COMPENSATION, FEES PAID FOR PARTICIPATION IN ADVISORY BOARDS, INVESTIGATIVE BOARDS OR OTHER COMMITTEES, ETC. FROM THE BRAZILIAN OR INTERNATIONAL PHARMACEUTICAL, ORTHOSIS, PROSTHESIS, EQUIPMENT AND IMPLANTS INDUSTRY: - Abbott: cardiology - Libbs: cardiology - Bayewr: cardiology - Novo Nordisk: cardiology OTHER RELATIONSHIPS FUNDING OF CONTINUING MEDICAL EDUCATION ACTIVITIES, INCLUDING TRAVEL, ACCOMMODATION AND REGISTRATION IN CONFERENCES AND COURSES, FROM THE BRAZILIAN OR INTERNATIONAL PHARMACEUTICAL, ORTHOSIS, PROSTHESIS, EQUIPMENT AND IMPLANTS INDUSTRY: - AstraZeneca - Bayer ANY ECONOMICALLY RELEVANT EQUITY INTEREST IN COMPANIES IN THE HEALTHCARE OR EDUCATION INDUSTRY OR IN ANY COMPANIES COMPETING WITH OR SUPPLYING TO SBC: - Instituto de Hipertensão de Minas Gerais, Cardio Check Up
Maria Carolina Feres de Almeida Soeiro	Nothing to be declared
Maria da Consolação Vieira Moreira	Nothing to be declared
Maria Del Pilar Estevez Diz	Nothing to be declared
Maria Verônica Câmara dos Santos	Nothing to be declared
Marianna Deway Andrade Dracoulakis	FINANCIAL DECLARATION A - ECONOMICALLY RELEVANT PAYMENTS OF ANY KIND MADE TO (i) YOU, (ii) YOUR SPOUSE/PARTNER OR ANY OTHER PERSON LIVING WITH YOU, (iii) ANY LEGAL PERSON IN WHICH ANY OF THESE IS EITHER A DIRECT OR INDIRECT CONTROLLING OWNER, BUSINESS PARTNER, SHAREHOLDER OR PARTICIPANT; ANY PAYMENTS RECEIVED FOR LECTURES, LESSONS, TRAINING INSTRUCTION, COMPENSATION, FEES PAID FOR PARTICIPATION IN ADVISORY BOARDS, INVESTIGATIVE BOARDS OR OTHER COMMITTEES, ETC. FROM THE BRAZILIAN OR INTERNATIONAL PHARMACEUTICAL, ORTHOSIS, PROSTHESIS, EQUIPMENT AND IMPLANTS INDUSTRY: - Pfizer: cardiology - Bayer: Xarelto - Daichii: Lixiana - Servier: cardiology OTHER RELATIONSHIPS FUNDING OF CONTINUING MEDICAL EDUCATION ACTIVITIES, INCLUDING TRAVEL, ACCOMMODATION AND REGISTRATION IN CONFERENCES AND COURSES, FROM THE BRAZILIAN OR INTERNATIONAL PHARMACEUTICAL, ORTHOSIS, PROSTHESIS, EQUIPMENT AND IMPLANTS INDUSTRY: - Pfizer: Eliquis
Marília Harumi Higuchi dos Santos Rehder	FINANCIAL DECLARATION A - ECONOMICALLY RELEVANT PAYMENTS OF ANY KIND MADE TO (i) YOU, (ii) YOUR SPOUSE/PARTNER OR ANY OTHER PERSON LIVING WITH YOU, (iii) ANY LEGAL PERSON IN WHICH ANY OF THESE IS EITHER A DIRECT OR INDIRECT CONTROLLING OWNER, BUSINESS PARTNER, SHAREHOLDER OR PARTICIPANT; ANY PAYMENTS RECEIVED FOR LECTURES, LESSONS, TRAINING INSTRUCTION, COMPENSATION, FEES PAID FOR PARTICIPATION IN ADVISORY BOARDS, INVESTIGATIVE BOARDS OR OTHER COMMITTEES, ETC. FROM THE BRAZILIAN OR INTERNATIONAL PHARMACEUTICAL, ORTHOSIS, PROSTHESIS, EQUIPMENT AND IMPLANTS INDUSTRY: - Eli Lilly from Brazil OTHER RELATIONSHIPS EMPLOYMENT RELATIONSHIP WITH THE BRAZILIAN OR INTERNATIONAL PHARMACEUTICAL, ORTHOSIS, PROSTHESIS, EQUIPMENT AND IMPLANTS INDUSTRY, AS WELL AS ANY EMPLOYMENT RELATIONSHIP WITH HEALTH INSURANCE COMPANIES OR MEDICAL AUDIT COMPANIES (INCLUDING PART-TIME JOBS) IN THE YEAR TO WHICH YOUR DECLARATION REFERS: - Pharmacovigilance Medical Manager
Patricia Tavares Felipe Marcatti	Nothing to be declared
Paulo Marcelo Gehm Hoff	FINANCIAL DECLARATION A - ECONOMICALLY RELEVANT PAYMENTS OF ANY KIND MADE TO (i) YOU, (ii) YOUR SPOUSE/PARTNER OR ANY OTHER PERSON LIVING WITH YOU, (iii) ANY LEGAL PERSON IN WHICH ANY OF THESE IS EITHER A DIRECT OR INDIRECT CONTROLLING OWNER, BUSINESS PARTNER, SHAREHOLDER OR PARTICIPANT; ANY PAYMENTS RECEIVED FOR LECTURES, LESSONS, TRAINING INSTRUCTION, COMPENSATION, FEES PAID FOR PARTICIPATION IN ADVISORY BOARDS, INVESTIGATIVE BOARDS OR OTHER COMMITTEES, ETC. FROM THE BRAZILIAN OR INTERNATIONAL PHARMACEUTICAL, ORTHOSIS, PROSTHESIS, EQUIPMENT AND IMPLANTS INDUSTRY: - Exelixis: oncology - Bayer: oncology - Lilly: oncology - United B - RESEARCH FUNDING UNDER YOUR DIRECT/PERSONAL RESPONSIBILITY (DIRECTED TO THE DEPARTMENT OR INSTITUTION) FROM THE BRAZILIAN OR INTERNATIONAL PHARMACEUTICAL, ORTHOSIS, PROSTHESIS, EQUIPMENT AND IMPLANTS INDUSTRY: - Exelixis: oncology - Bayer: oncology - AstraZeneca: oncology - United - Lilly - Pfizer - Sanofi - Roche - BMS - MSD - Merck - Novartis OTHER RELATIONSHIPS PARTICIPATION IN PROCUREMENT COMMITTEES FOR SUPPLIES OR DRUGS IN HEALTH INSTITUTIONS OR ANY SIMILAR ROLES TAKEN: - Pharmacy Committee - ICESP
Renata do Val	Nothing to be declared
Ricardo Pavanello	OTHER RELATIONSHIPS FUNDING OF CONTINUING MEDICAL EDUCATION ACTIVITIES, INCLUDING TRAVEL, ACCOMMODATION AND REGISTRATION IN CONFERENCES AND COURSES, FROM THE BRAZILIAN OR INTERNATIONAL PHARMACEUTICAL, ORTHOSIS, PROSTHESIS, EQUIPMENT AND IMPLANTS INDUSTRY: - Bayer: Continuing Distance Education Program
Roberto Kalil Filho	Nothing to be declared
Sílvia Marinho Martins Alves	Nothing to be declared
Silvia Moreira Ayub Ferreira	FINANCIAL DECLARATION A - ECONOMICALLY RELEVANT PAYMENTS OF ANY KIND MADE TO (i) YOU, (ii) YOUR SPOUSE/PARTNER OR ANY OTHER PERSON LIVING WITH YOU, (iii) ANY LEGAL PERSON IN WHICH ANY OF THESE IS EITHER A DIRECT OR INDIRECT CONTROLLING OWNER, BUSINESS PARTNER, SHAREHOLDER OR PARTICIPANT; ANY PAYMENTS RECEIVED FOR LECTURES, LESSONS, TRAINING INSTRUCTION, COMPENSATION, FEES PAID FOR PARTICIPATION IN ADVISORY BOARDS, INVESTIGATIVE BOARDS OR OTHER COMMITTEES, ETC. FROM THE BRAZILIAN OR INTERNATIONAL PHARMACEUTICAL, ORTHOSIS, PROSTHESIS, EQUIPMENT AND IMPLANTS INDUSTRY: - Abbott: MitraClip OTHER RELATIONSHIPS FUNDING OF CONTINUING MEDICAL EDUCATION ACTIVITIES, INCLUDING TRAVEL, ACCOMMODATION AND REGISTRATION IN CONFERENCES AND COURSES, FROM THE BRAZILIAN OR INTERNATIONAL PHARMACEUTICAL, ORTHOSIS, PROSTHESIS, EQUIPMENT AND IMPLANTS INDUSTRY: - Abbott: mechanical circulatory assistance
Silvia Moulin Ribeiro Fonseca	Nothing to be declared
Stephanie Itala Rizk	Nothing to be declared
Thiago Liguori Feliciano da Silva	Nothing to be declared
Vanderson Rocha	Nothing to be declared
Veronica Cristina Quiroga Fonseca	Nothing to be declared
Wilson Mathias Junior	Nothing to be declared
Yana Novis	Nothing to be declared

## 1. Introduction

Cardiovascular disease (CVD) and cancer are currently the leading causes of mortality worldwide and in Brazil.^1^^–^^3^ The recent demographic and epidemiological transitions in Brazil have determined an increase in the population's life expectancy, today around 76 years, and a change in the health profile, in which chronic diseases and their complications prevail.^4^

These factors pose important challenges and require the development of a health policy agenda for the management of the ongoing transitions. The technological advances, the shortage of cost-effectiveness analyses and, in higher education settings, the little value attributed to health access and promotion, as well as to disease prevention, require the implementation of guidelines and consensus statements. These guidelines and consensus statements are aimed at helping the use of systematized protocols to adapt the clinical practice regardless of the geographic location of the health facilities and the heterogeneity of their resources.

Recent advances in cancer detection and treatment have resulted in an exponential increase in the number of cancer survivors around the world. According to a recent estimate, by 2026, the United States will have 20 million cancer survivors, 50% of whom will be older than 70 years.^5^^,^^6^ The care of an older population with history of cancer and CVD, compounded by the potential cardiovascular toxicity of the oncological treatment, requires specialists in the ‘cancer-CVD' interaction.^7^

In 1967, anthracycline-induced cardiotoxicity was first described.^8^ In 1971, a study reported that anthracycline-induced cardiotoxicity was dose-dependent and that the cardiac damage might be irreversible.^9^ Some years later, risk factors for chemotherapy-related ventricular dysfunction were identified, and biomarkers, such as troponin and B-type natriuretic peptide (BNP), were related to the prediction of cardiovascular events.^10^^,^^11^ Those findings were the cornerstone of cardio-oncology.

Cardio-oncology is the field of science devoted to the early diagnosis and proper management of CVD in patients with the current or previous diagnosis of cancer. Furthermore, cardio-oncology comprises the analysis of not only the cardiovascular risks related to the oncological diagnosis, but also the patient's needs before, during and after the treatment. Cardio-oncology specialists should follow patients up since their diagnosis, through all treatment phases, and even after their cure, when the patients are called cancer survivors. The need for expansion of cardio-oncology relates directly to the epidemiology of cancer and CVD, the risk factors they share, and the multiplicity of treatments with distinct toxicities to the cardiovascular system (Figure 1).^12^^,^^13^

**Figure 1:** The current frontiers of cardio-oncology

In 2011, the Brazilian Society of Cardiology (SBC) and the Brazilian Society of Clinical Oncology (SBOC) pioneered in joining forces to publish the I Guideline on Cardio-Oncology.^14^ In 9 years, cardio-oncology has significantly grown as a discipline because of the following factors: a) remarkable advances in cancer treatment; b) understanding of multidisciplinarity and integration of cardiology, oncology and hematology as essentials for the care of cancer patients; c) implementation of fellowship programs across the world and insertion of ‘cardio-oncology' in the curriculum of some cardiology residency training programs; d) growth of research in basic and clinical areas; and e) creation of important journals dedicated to the subject, such as *JACC CardioOncology* and *Cardio-Oncology*.^15^^,^^16^

It is worth noting that, in 2019, Brazil hosted the *V Global Cardio-Oncology Summit*, to which specialists from several countries and approximately 600 professionals (cardiologists, oncologists, hematologists, nurses, physical therapists, pharmacists, physical educators) attended. The journal *Frontiers in Cardiovascular Medicine* published 89 abstracts, and the *JACC CardioOncology* published *“Proceedings From the Global Cardio-Oncology Summit - The Top 10 Priorities to Actualize for CardioOncology*”.^17^^,^^18^

The SBC and the SBOC, aiming at knowledge updating and promotion of a rational and systematic approach to cardiovascular complications in oncology patients, have gathered a team of experts to create new strategies, issue evidence-based recommendations, and develop multiprofessional healthcare, which will provide the proper management of that increasing category of patients.

The goals of the *Brazilian Cardio-Oncology Guideline - 2020* are as follows: 1) to demystify the belief of CVD as a barrier to the effective treatment of cancer patients; 2) to prevent and reduce the risks of treatment-related cardiotoxicity; 3) to promote the interaction among medical specialties (cardiology, hematology and oncology) to agree the best strategy for patient's care, weighing the risks and benefits of the treatment; 4) to propose the unification of terminologies and definitions of the cardiovascular complications of cancer patients, aiming at homogenizing care and research; 5) to disclose the evidence available on the management of cardiovascular complications in oncology patients, aiming at their early diagnosis by use of cardiovascular function monitoring before, during and after the treatment; 6) to promote proper treatment, with the participation of oncologists and hematologists, based on scientific evidence, risk analysis and care personalization, considering the patient's preferences; and 7) to boost research and knowledge spread in cardio-oncology (Figure 1).

The *Brazilian Cardio-Oncology Guideline - 2020* gathers evidence on the cardiovascular complications of cancer patients available up to 2020.

## 2. Methods

The *Brazilian Cardio-Oncology Guideline - 2020* abided by the ongoing recommendations. A team of experts in cardiology, hematology and oncology formed a committee to elaborate this manuscript. Participants were chosen based on their prominence in their fields, their participation in the International Cardio-Oncology Society (ICOS), SBC and SBOC, in addition to their scientific production.

A bibliographic search was conducted in PubMed in the period from 1975 to July 2020 with the following keywords: *cardiotoxicity, cancer, immunotherapy, cardiooncology, cardiovascular complications, targeted therapy, radiotherapy, vascular toxicity, heart failure, ventricular dysfunction, pericardial disease, coronary disease, thromboembolism, arrhythmias, hypertension, individual drug names*. The manuscript was sent electronically to all participants, and, after they all agreed on its content, it was formatted and sent to publication.

The classes of recommendation and levels of evidence used in this guideline were as follows:

Classes of recommendations:

Grade I – there is conclusive evidence, or, failing that, a consensus that the procedure is safe and useful/effective.

Grade II – there is conflicting evidence and/or divergent opinions on the safety and utility/effectiveness of the procedure:

- Grade IIA: weight of the evidence/opinion is in favor of the procedure. Most experts approve;
- Grade IIB: safety and utility/effectiveness are less well established, with no predominance of opinions in favor.

Grade III – there is evidence and/or expert consensus that the procedure is not useful/effective and, in some cases, can even be harmful.

Levels of Evidence:

Level A – data obtained from multiple, large, concordant randomized studies and/or robust meta-analyses of randomized clinical studies.

Level B – data obtained from a less robust metaanalysis, based on a single randomized trial or on non-randomized (observational) studies.

Level C – data obtained from consensus expert opinions.

## 3. Diagnosis and Management of Cardiovascular Complications in Cancer Patients

### 3.1. Initial Cardiological Assessment

The different types of cancer treatment, such as chemotherapy, immunotherapy, and radiotherapy, can result in damage to the cardiovascular system. Patients with previous CVD or cardiovascular risk factors have the highest likelihood of complications from cancer treatment. Thus, the treatment and control of cardiovascular risk factors in cancer patients are recommended.^19^^–^^21^

The consultation of cancer patients with a cardiologist should comprise the control of cardiovascular risk factors, cardioprotective measures, adhesion to treatment, and a strategy to enable the early diagnosis of cardiac damage (I, B).

Patients with cardiovascular risk factors or already established CVD and who will undergo a potentially cardiotoxic treatment [anthracyclines, anti-HER2 (human epidermal growth factor receptor 2) agents, alkylating agents, inhibitors of vascular endothelial growth factor (VEGF) signaling, proteasome inhibitors and immune checkpoint inhibitors (ICIs)] should be assessed by a cardiologist at the beginning of therapy and followed up according to specific protocols (I, B). Table 1 shows the antineoplastic treatments most associated with cardiovascular toxicity. Figure 2 shows the factors associated with a higher risk for cardiotoxicity.

**Figure 2:** Predisposing factors for the development of cardiotoxicity in cancer patients. Adapted from Zamorano et al.^22^

**Table 1:** Antineoplastic therapies associated with cardiovascular toxicity

Classes of antineoplastic drugs	Cardiovascular toxicity
Radiotherapy	Myocardial ischemia and infarction Pericardial disease Heart valve disease Myocarditis Cardiac arrhythmia
Anthracyclines (doxorubicin, epirubicin, daunorubicin, idarubicin, mitoxantrone)	Heart failure Asymptomatic ventricular dysfunction Myocarditis Pericarditis Atrial and ventricular arrhythmias
Alkylating agents (cyclophosphamide, ifosfamide, melphalan)	Arrhythmias Ventricular dysfunction Coronary artery disease
Platinum drugs (cisplatin, carboplatin, oxaliplatin)	Coronary thrombosis Myocardial ischemia Arterial hypertension
Antimetabolite drugs (5-fluorouracil, capecitabine)	Myocardial ischemia Coronary vasospasm Atrial and ventricular arrhythmias
HER2-targeted therapies (trastuzumab, pertuzumab, T-DM1, lapatinib, neratinib)	Heart failure Asymptomatic ventricular dysfunction Arterial hypertension
Inhibitors of VEGF signaling: Tyrosine kinase inhibitors (sunitinib, pazopanib, sorafenib, axitinib, tivozanib, cabozantinib, regorafenib, lenvatinib, vandetinib) Monoclonal antibodies (bevacizumab, ramucirumab)	Arterial hypertension Heart failure Asymptomatic ventricular dysfunction Myocardial ischemia and infarction QTc prolongation
Multi-targeted tyrosine kinase inhibitors: Second- and third-generation BCR-ABL tyrosine kinase inhibitors (ponatinib, nilotinib, dasatinib, bosutinib)	Arterial thrombosis (myocardial infarction, stroke and occlusive peripheral vascular disease*) Venous thromboembolism Arterial hypertension Heart failure Asymptomatic ventricular dysfunction Atherosclerosis** QTc prolongation** Pulmonary hypertension***
Other multi-targeted tyrosine kinase inhibitors: ALK inhibitors (crizotinib, ceritinib) PI3-AKT-mTOR inhibitors (everolimus, sirolimus) Bruton's tyrosine kinase inhibitors (ibrutinib) EGFR tyrosine kinase inhibitor (osimertinib)	Bradycardia, QTc prolongation Hyperglycemia, dyslipidemia Atrial fibrillation Heart failure, atrial fibrillation, QTc prolongation Atrial fibrillation, heart failure
Therapy of multiple myeloma: Proteasome inhibitors (carfilzomib, bortezomib, ixazomib) Immune modulators (lenalidomide, thalidomide, pomalidomide)	Heart failure**** Asymptomatic ventricular dysfunction**** Myocardial ischemia and infarction Atrial and ventricular arrhythmias Venous thromboembolism Arterial thrombosis Arterial hypertension
BRAF and MEK inhibitors: (dabrafenib + trametinib, vemurafenib + cobimetinib, encorafenib + binimetinib)	Heart failure Asymptomatic ventricular dysfunction Arterial hypertension QTc prolongation*****
Antiandrogen therapies: GnRH agonists (goserelin, leuprolide) GnRH antagonists (degarelix) Antiandrogens (abiraterone)	Atherosclerosis Myocardial ischemia and infarction Diabetes mellitus Arterial hypertension
Immune checkpoint inhibitors: (nivolumab, ipilimumab, durvalumab, pembrolizumab, atezolizumab, avelumab)	Myocarditis Heart failure Atrial and ventricular arrhythmias Myocardial ischemia

* Associated with ponatinib

** Associated with ponatinib and nilotinib

*** Associated with dasatinib

**** Associated with carfilzomib

***** *Associated with vemurafenib and cobimetinib. EGFR: epidermal growth factor receptor; GnRH: gonadotropin releasing hormone; HER2: human epidermal growth factor receptor 2; QTc: corrected QT; T-DM1: ado-trastuzumab emtansine; VEGF: vascular endothelial growth factor*.

The multiprofessional team assessing the cancer patient should weigh the risks and supposed benefits of the therapy and implement strategies to prevent cardiovascular damage (IIa, C).

Measuring and approaching the cardiovascular risk factors according to consensus and guidelines are recommended (I, A).

In the initial cardiological assessment, the following are recommended: anamnesis, physical examination, electrocardiogram (ECG), chest X-ray, complete blood count, measurement of electrolytes and biomarkers [N-terminal pro-BNP (NT-proBNP) and troponin I or high-sensitivity troponin T], folic acid, vitamins D and B12, glycemia, lipid profile, as well as kidney, liver and thyroid function (I, A) (Figure 3).

**Figure 3:** Initial cardiologist assessment. *Ideally combined with LVEF three-dimensional assessment and myocardial strain quantification by speckle tracking. CMRI: cardiac magnetic resonance imaging; CVD: cardiovascular disease; ECG: electrocardiogram; echo: echocardiography; HF: heart failure; Ica: serum ionic calcium; K: serum potassium; LV: left ventricular; LVEF: left ventricular ejection fraction; Mg: serum magnesium; Na: serum sodium; NT-proBNP: N-terminal pro-B-type natriuretic peptide; X-ray: radiography

In addition, in baseline and serial assessment according to the treatment regimen, transthoracic echocardiography with color Doppler, ideally three-dimensional, is recommended, with analysis of left ventricular ejection fraction (LVEF), diastolic function, and myocardial deformation with strain quantification by use of the speckle tracking technique (I, A).

Collaboration between cardiologists, oncologists and hematologists is recommended to ensure the proper and beneficial treatment to cancer patients (IIa, A).

### 3.2. Diagnosis of Cardiotoxicity in Cancer Patients

Cardiotoxicity can be diagnosed by confirming the presence of a new cardiovascular alteration (clinical and/or in biomarkers and/or in imaging) during or after treatment, once other etiologies have been excluded (I, B).

Echocardiography is the method of choice to detect myocardial dysfunction related to the oncological treatment. Three-dimensional echocardiography is the best echocardiographic method to measure LVEF in cancer patients. When not available or in the presence of limitations, biplane Simpson method is recommended (I, A).

Ventricular dysfunction related to cancer therapy is defined as a reduction ≥ 10% in LVEF to a value below the lower limit of the normal range (LVEF < 50%). A new cardiovascular imaging test should be performed in 2 to 3 weeks (I, B).

That LVEF reduction occurs in the course of treatment, and can be classified as symptomatic or asymptomatic and reversible or irreversible (I, B).

Global longitudinal strain (GLS) is a highly sensitive tool to predict later LVEF reduction. A GLS reduction ≥ 15% in regard to baseline is considered abnormal and an early marker of ventricular dysfunction (I, B).

Diastolic function analysis is recommended in oncological patients, both before therapy starts and during follow-up (IIa, C). However, there is no evidence that the treatment should be interrupted based on diastolic function.

Radionuclide ventriculography is not recommended for ventricular function assessment in cancer patients (III, B).

Cardiac magnetic resonance imaging (CMRI) is the gold-standard method to assess cardiac function. It enables structural assessment and tissue characterization, being recommended when echocardiography cannot be performed, in the presence of infiltrative diseases, for pericardial and myocardial evaluation, and for the detection of masses and tumors (IIa, B). In addition, CMRI can assess prognosis by analyzing myocardial fibrosis.

The routine use of biomarkers during a potentially cardiotoxic treatment has not been well established. Monitoring cardiotoxicity by measuring biomarkers can be considered for the early detection of myocardial damage in patients at high risk due to previous factors or those exposed to drugs, such as anthracyclines and trastuzumab (IIa, B). Neither the best time for measuring biomarkers regarding chemotherapy (during chemotherapy, 24 hours after, 48 hours after or later) nor the best management when high levels of biomarkers are detected are known. In addition, in the course of treatment, the same analysis kits of biomarkers, such as high-sensitivity troponin and NT-proBNP assays, should be used (IIa, C).

High levels of biomarkers (NT-proBNP and troponin) indicate increased risk for cardiotoxicity (I, A).

On initial assessment and throughout treatment, ECG should be performed. The QTc should be calculated by using Bazett's [QT / (RR)^1/2^] or Fridericia's [QT / (RR)^1/3^] formula, and the same method should be used for the patient's serial assessment. In cancer patients, the Fridericia's formula is preferred, because it undergoes less change in the presence of tachycardia or bradycardia (IIa, C).

Table 2 describes the cardiovascular diagnostic methods and their major advantages, uses, and limitations.

**Table 2:** Cardiovascular diagnostic methods and their major advantages, uses, and limitations

## 4. Ventricular Dysfunction

Ventricular dysfunction is one of the most severe complications from cancer treatment, characterized by high morbidity and mortality rates. It may appear during therapy or years after completion of therapy and even so be consequent to drug toxicity.^23^ The classic model of ventricular dysfunction as a form of cardiotoxicity is secondary to the use of anthracyclines, which are widely used to treat sarcoma, lymphoma, leukemia, and breast cancer.^24^^,^^25^

The different chemotherapy and immunotherapy drugs associated with ventricular dysfunction result in different phenotypes in patients, ranging from asymptomatic mild and reversible dysfunction to severe, clinically manifest and irreversible heart failure (HF). Pediatric cancer survivors are up to 15 times more likely to develop HF than controls matched for other risk factors.^26^

Predicting cardiotoxicity is a challenge, because of the multiplicity of drugs to which patients are exposed throughout life, in addition to the often-present cardiovascular risk factors. It is worth noting the multiple drug interactions of the different therapeutic regimens, such as those of anthracyclines with cyclophosphamide and anthracyclines with trastuzumab.

In recent years, with the introduction of new chemotherapy drugs and the advent of immunotherapy, in addition to the introduction of protocols for early detection of cardiotoxicity, ventricular dysfunction has been increasingly diagnosed. Table 3 shows the antineoplastic drugs more often associated with ventricular dysfunction.

**Table 3:** Chemotherapy drugs associated with ventricular dysfunction

Chemotherapy drugs	Incidence (%)
Anthracyclines (dose-dependent)	
Doxorubicin (Adriamycin) 400 mg/m^2^ 550 mg/m^2^ 700 mg/m^2^	3-5 7-26 18-48
Idarubicin > 90 mg/m^2^	5-18
Epirubicin > 900 mg/m^2^	0.9-11.4
Mitoxantrone > 120 mg/m^2^	2.6
Liposomal doxorubicin >900 mg/m^2^	2
Cyclophosphamide	7-28
Ifosfamide < 10 g/m^2^ 12.5-16 g/m^2^	0.5 17
**Antimetabolites**	
Clofarabine	27
**Antimicrotubule agents**	
Docetaxel	2.3-13
Paclitaxel	< 1
**HER2 targeted therapies**	
Trastuzumab	1.7-20.1
Pertuzumab	0.7-1.2
Monoclonal antibodies	
Bevacizumab	1.6-4
**Tyrosine kinase inhibitors**	
Sunitinib	2.7-19
Pazopanib	7-11
Sorafenib	4-8
Dasatinib	2-4
Imatinib	0.2-2.7
Lapatinib	0.2-1.5
Nilotinib	1
**Proteasome inhibitors**	
Carfilzomib	11-25
Bortezomib	2-5

*HER2: human epidermal growth factor receptor 2. Adapted from Zamorano et al.22*

### 4.1. Anthracyclines

Anthracyclines consist in a group of antineoplastic drugs known to be effective in treating sarcoma, lymphoma, leukemia, and breast cancer. Their clinical use is limited by cardiotoxicity characterized by ventricular dysfunction and HF, which are the main causes of mortality in cancer survivors.

The toxicity of anthracyclines is highly variable and can occur in up to 50% of the patients, depending on the patient's risk factors and the pharmacological properties of the chemotherapy drugs, such as cumulative dose. For example, doxorubicin is associated with a 5% incidence of HF at the cumulative dose of up to 400 mg/m^2^, and that incidence can reach 50% if the dose exceeds 700 mg/m^2^.^27^ A recent study with 2625 patients in a 5-year follow-up has shown a 9% overall incidence of anthracycline-induced cardiotoxicity, and 98% of the cases were asymptomatic and occurred in the first year.^24^

Cardiotoxicity can be acute, early or late, reversible or irreversible. Acute toxicity is characterized by the presence of supraventricular arrhythmia, left ventricular dysfunction and electrocardiographic changes, which appear right after anthracycline infusion in up to 1% of the patients, being usually reversible. Acute ventricular dysfunction can be a predictor of HF, which can be subacute or chronic. Early cardiotoxicity appears in the first year of treatment, while late cardiotoxicity appears years after treatment, on average, 7 years after completion of treatment.^28^

There is no predictor of the reversibility/irreversibility of the anthracycline-induced toxicity. However, the elevation in the levels of biomarkers and its persistence can identify patients at high risk for irreversibility.^29^

The tendency towards cardiotoxicity varies with the different treatment regimens, and doxorubicin is the anthracycline most associated with ventricular dysfunction. Cardiotoxicity is dose-dependent, and reducing the cumulative dose is a way to minimize it. Changes in infusion, such as prolonging its duration, splitting the dose and using liposomal formulations, can prevent cardiotoxicity.^24^ A recent experimental study has suggested that ischemic preconditioning might prevent doxorubicin-induced cardiotoxicity.^30^

Mechanistic studies have shown that anthracycline-induced ventricular dysfunction is associated with: 1) damage to the sarcoplasmic reticulum and mitochondria; 2) changes in myofibrillar structure and function; 3) total or partial loss of matrix interspersed with collagen plaques in the interstitium; 4) change in the excitation-contraction coupling and calcium flow; 5) apoptosis; 6) changes in iron metabolism; and 7) loss of the regeneration capacity of the cardiac muscle and coronary endothelial cells. The consequence is dysfunction and hypertrophy of the remaining myocytes.^31^ The common trigger for those events seems to be related to the oxidative stress caused by the production of reactive oxygen species, in addition to the inhibition of topoisomerase 2β, resulting in damage to membranes, proteins and DNA. The following observations support the importance of oxidative stress in anthracycline-induced cardiotoxicity: a) over-expression of metallothionein, a free radical scavenger, in the heart of transgenic mice minimizes the doxorubicin-induced injury; b) inhibition of the formation of peroxynitrite, a reactive oxidant produced from nitric oxide and superoxide, improves the cardiac function of mice exposed to doxorubicin; c) probucol, a strong antioxidant, prevents glutathione peroxidase reduction and reduces doxorubicin-related myocardial lipid peroxidation in a murine model; d) dexrazoxane is a chelating agent like EDTA that can prevent anthracycline damage via iron binding, which acts as a cofactor for free radicals.^32^ Diastolic dysfunction due to cumulative dose-dependent toxicity can be observed with a cumulative dose of 200 mg/m^2^, while systolic dysfunction is usually observed with doses over 400 mg/m^2^, with variability according to an individual threshold. However, impaired diastolic function has been observed with the cumulative dose of only 120 mg/m^2^.^33^

Table 4 shows the risk factors associated with a higher likelihood of anthracycline-induced toxicity, of which previous heart disease, cumulative dose and fast drug infusion stand out. However, in the presence of the same risk factors, there is an important variability in the occurrence of cardiotoxicity among patients, which might be related to genetic factors and interactions with unknown factors.

**Table 4:** Risk factors for anthracycline-related cardiotoxicity

Polymorphisms in ATP-binding cassette (ABC) transporter genes are associated with anthracycline cardiomyopathy. Those transporters play an important role in drug resistance via cellular efflux of drugs, including anthracyclines. Reduced activity can lead to intracellular accumulation of anthracycline and cellular toxicity. Variants in that family of genes replicated in cohorts of childhood cancer patients include *ABCC5* (A-1629T, rs7627754), associated with a significant LVEF reduction in T-allele homozygous survivors.^34^ In addition, a variant in histamine methyltransferase *HNMT* (rs17583889) confers risk in young patients exposed to anthracyclines.^35^ Table 5 shows the pharmacogenetic variants that predispose to anthracycline-related cardiotoxicity.

**Table 5:** Variantes farmacogenéticas associadas à cardiotoxicidade das antraciclinas

During treatment with anthracyclines, clinical and echocardiographic monitoring is recommended at a pre-established frequency or out of protocol in the presence of HF signs and symptoms.^21^ Ideally, the echocardiographic assessment should comprise biventricular systolic and diastolic function analysis (I, A) (Figure 4).

**Figure 4:** Echocardiographic monitoring and analysis of biomarkers in patients using anthracyclines. Echo: echocardiogram; NT-proBNP: N-terminal pro-B-type natriuretic peptide; QT: chemotherapy

### 4.2. HER2-targeted Therapies

Trastuzumab is a monoclonal antibody targeted at the human epidermal growth factor receptor 2 (HER2 or ErbB2). For 15-20% of the patients with breast cancer whose tumors over express HER2, therapy with trastuzumab significantly reduces mortality.^36^^,^^37^ Its use is associated with a considerable risk of cardiotoxicity, clinically manifested by an asymptomatic decline in LVEF and, less commonly, by symptomatic HF.^38^ After the introduction of trastuzumab, three other anti-HER2 agents were developed: lapatinib, a tyrosine kinase inhibitor of the epidermal growth factor (EGFR), ERBB1 and HER2; ado-trastuzumab emtansine (T-DM1), a conjugated antibody composed by trastuzumab, a thioester linker and an antimitotic maytansine derivative; and pertuzumab, a monoclonal antibody that binds to the subdomain II of the HER2 extracellular domain and prevents HER2 homo- and heterodimerization with other HER receptors. Although data on those new drugs are scarce, there is evidence that T-DM1 and pertuzumab are less cardiotoxic than trastuzumab.^39^

The LVEF decline rate consequent to trastuzumab use varies in the literature. Recent studies have reported, in 15% to 40% of the patients on trastuzumab, a LVEF reduction of at least 10%, and, in 18% of the patients, a LVEF drop to less than 53%.^40^^,^^41^ Symptomatic HF has been reported in 0.6% to 8.7% of patients.^40^

One difference between the toxicity of anti-HER2 agents and that of anthracyclines is the reversibility of the former in most cases. The determinants of reversibility are previous cardiovascular function and the extent of LVEF decline related to treatment. A recent study has shown that all LVEF declines smaller than 10% were reversible. However, for LVEF declines greater than 10%, reversibility was observed in 91% of the patients with normal baseline cardiovascular function as compared to only 71.4% of those with reduced LVEF prior to exposure.^42^ Some studies have reported that, even in the presence of cardiotoxicity, 70% to 80% of patients continue receiving trastuzumab and that the highest likelihood of cardiovascular toxicity and mortality related to treatment is observed in patients with previously reduced LVEF.^43^

Trastuzumab-induced ventricular dysfunction and clinically manifest HF are usually reversible after chemotherapy interruption and/or after beginning HF treatment. The mechanisms of the anti-HER2 therapy-induced cardiotoxicity include structural and functional changes in contractile proteins and mitochondria, but rarely lead to cellular death, explaining the potential reversibility. The interruption of trastuzumab treatment is associated with an increase in cancer recurrence, and cardiotoxicity is the major responsible for drug suspension.^44^

Table 6 shows the risk factors for cardiotoxicity induced by anti-HER2 therapy.

**Table 6:** Anti-HER2 therapy and risk factors for cardiotoxicity

Agents	Risk factors for cardiotoxicity
Anti-HER2 Trastuzumab Pertuzumab T-DM1	Previous or concomitant treatment with anthracycline
	Age > 50 years
	Body mass index > 30 kg/m^2^
	Previous left ventricular dysfunction
	Arterial hypertension
	Previous mediastinal radiotherapy

*HER2: human epidermal growth factor receptor 2; T-DM1: ado-trastuzumab emtansine*.

During treatment with trastuzumab, clinical and echocardiographic monitoring is recommended according to protocol or in the presence of HF signs and symptoms (I, A) (Figure 5).

**Figure 5:** Echocardiographic monitoring and analysis of biomarkers in patients using anti-HER2 drugs. Echo, echocardiogram; RF, risk factors; HF, heart failure; NT-proBNP, N-terminal pro-B-type natriuretic peptide

### 4.3. VEGF Inhibitors

The inhibition of VEGF signaling pathways benefits thousands of cancer patients, but some chemotherapy drugs of that class are associated with the risk of cardiotoxicity, which can be reversible or irreversible, particularly in the presence of concomitant or previous use of other chemotherapy drugs.^45^^–^^47^

The relative risk of congestive HF in bevacizumab-treated patients was 4.74 (95% CI: 1.6-11.18; p = 0.001) compared to that of the placebo group.^45^ In addition, other drugs, such as sunitinib, pazopanib and axitinib, have been associated with the development of ventricular dysfunction. A meta-analysis including 10 553 patients has reported congestive HF incidence of 3.2% (95% CI: 1.8% - 5.8%) with the use of VEGF tyrosine kinase inhibitors.^47^

Systemic arterial hypertension (SAH) is a common complication of that class of chemotherapy drugs, and some studies have suggested that the proper treatment of SAH might reduce the risk of HF.^48^ The prognosis of patients who develop cardiotoxicity associated with VEGF inhibitors is hard to assess, because candidates for treatment with such drugs usually have metastatic disease and reduced life expectancy. Most cases reverse with the treatment of ventricular dysfunction. Table 7 shows the risk factors for cardiotoxicity.

**Table 7:** Therapy with VEGF inhibitors and risk factors for cardiotoxicity

VEGF Inhibitors	
Antibodies Bevacizumab Ramucirumab	Pre-existing HF, coronary artery disease, heart valve disease, ischemic heart disease Previous use of anthracycline
Tyrosine kinase inhibitors Sunitinib Pazopanib Axitinib Neratinib Afatinib Sorafenib Desatinib	Arterial hypertension Pre-existing heart disease

*HF: heart failure*.

#### 4.3.1. BCR-ABL Tyrosine Kinase Inhibitors

The BCR-ABL tyrosine kinase inhibitors have changed the prognosis of patients with chronic myeloid leukemia and gastrointestinal stromal tumors. The cardiotoxicity of imatinib has not been confirmed; however, nilotinib and ponatinib may be associated with cardiotoxicity involving HF, SAH, arrhythmias and thromboembolism.^49^

### 4.4. Therapies for Multiple Myeloma

Proteasome inhibitors are relatively new drugs to treat multiple myeloma. Bortezomib and carfilzomib belong to this class of drugs and can cause cardiovascular dysfunction. Proteasomes are protein complexes responsible for degrading dysfunctional proteins, being essential for the cardiomyocyte survival. The incidence of bortezomib-related HF is 4% and it can be compounded by the use of steroids.^50^ In addition to being irreversible, carfilzomib is the most potent proteasome inhibitor and can cause HF in up to 25% of the patients.^51^^,^^52^

### 4.5. BRAF and MEK Inhibitors

The combined BRAF-MEK inhibitor therapy is currently the first choice for metastatic BRAF*-*mutant melanoma, because it significantly improves patients' survival. So far, three BRAF inhibitors (dabrafenib, vemurafenib and encorafenib) and three MEK inhibitors (trametinib, cobimetinib and binimetinib) have been approved for the treatment of melanoma.^53^^–^^55^

Several studies have reported cardiovascular adverse effects associated with those inhibitors, mainly LVEF reduction (5-11%), SAH (10-15%), and QT interval prolongation.^56^^,^^57^ The inhibition of BRAF and MEK interferes with cardiovascular MAPK signaling, resulting in oxidative stress, cardiac myocyte apoptosis, and angiogenesis inhibition.^56^^,^^57^

A recent meta-analysis, including five randomized clinical trials and 2317 patients with melanoma and receiving BRAF and MEK inhibitors, has shown that the concomitant treatment with those inhibitors is associated with an increased risk for pulmonary embolism (4.4x), LVEF reduction (3.72x), and SAH (1.5x). There was no increase in the occurrence of arrhythmias, myocardial infarction, and QT prolongation. A higher risk for HF was detected in patients under the age of 55 years.^58^

### 4.6. Taxanes

Paclitaxel and docetaxel are used to treat several solid neoplasms. Cardiotoxicity is not frequent with this group of drugs, occurring in 12 out of 100 (RR: 0.9 [0.53 -1.54]).^59^ Docetaxel, in particular, seems to be associated with an increase in the occurrence of ventricular dysfunction. Some reports have suggested that taxanes should be avoided in patients with previous ventricular dysfunction, and the same non-use criteria of anthracyclines apply. Taxanes have been reported to cause sinus bradycardia, atrioventricular blocks, ventricular tachycardia, and ventricular extrasystoles. However, because taxanes are used in combination with anthracyclines, determining their cardiotoxicity potential is challenging.^36^^,^^60^

### 4.7. Immune Checkpoint Inhibitors

Immune checkpoint inhibitors have revolutionized cancer treatment. The ICIs modulate the immune system, inhibiting the apoptosis of T lymphocytes, restoring the antitumor cell response. Their anti-apoptotic action comprises the inhibition of CTLA-4 (ipilimumab), PD-1 (nivolumab, pembrolizumab), and PDL-1 (atezolizumab, durvalumab, avelumab) (Figure 6).^61^

**Figure 6:** Major immune checkpoint inhibitors related to cardiotoxicity and its management

The cardiotoxicity of ICIs can be divided into two categories: inflammatory adverse effects (myocarditis, pericarditis, and vasculitis) and non-inflammatory cardiovascular toxicity (Takotsubo-like syndrome, asymptomatic non-inflammatory ventricular dysfunction, and arrhythmias). Most cases reported are severe, with mortality rates of 50% in myocarditis, 21% in pericardial disease, and 6% in vasculitis.^62^ The major causes of mortality from myocarditis are arrhythmias and cardiogenic shock.^62^^–^^64^

The adverse events usually occur after the first or second dose of ICIs, but sporadic cardiovascular events have been reported up to 32 weeks after treatment. The prevalence of cardiovascular involvement is higher in patients on combined therapy, of the female sex, and older than 75 years. The prevalence of myocarditis varies from 0.06% to 0.3%.^62^^,^^63^

For patients who develop new cardiovascular symptoms during or right after treatment with ICIs or who have arrhythmia, conduction system abnormality or ventricular dysfunction on the echocardiogram, initiating cardiovascular investigation with measurement of biomarkers (troponin, NT-proBNP and C-reactive protein), ECG, viral panel test, strain echocardiography and CMRI is recommended to confirm the diagnosis and exclude viral myocarditis (IIa, C).

Endomyocardial biopsy should be considered in case of diagnostic suspicion even when the initial investigation is negative (IIa, C).

## 5. Radiotherapy

The current incidence of radiation-induced cardiotoxicity is hard to estimate, among other reasons, because of the long interval between exposure and the clinical manifestation of cardiotoxicity, the concomitant use of cardiotoxic chemotherapy, and the progressive improvement in radiation techniques in recent years with the consequent reduction in the incidence of cardiac structural damage. Some studies have reported relative risk of fatal cardiovascular events varying from 2.2% to 12.7% in lymphoma survivors and from 1% to 2.2% in breast cancer patients.^65^^,^^66^ Among survivors exposed to radiotherapy, the risk of ventricular dysfunction increases 4.9 times.^66^ Radiation-related cardiotoxicity is more frequent in patients with left-sided breast cancer^67^ and in those on the concomitant use of anthracyclines. The radiation-induced injury can affect the cardiac muscle, valves, pericardium, coronary arteries and conduction system,^68^ and can be diagnosed 10 to 15 years after radiotherapy.

## 6. Cardiotoxicity Prevention and Treatment

- Cardiotoxicity should be prevented in all cancer patients and the cardiovascular risk factors should be recognized since the initial consultation. The following measures are recommended: smoking and alcoholism cessation, implementation of a regular diet aimed at maintaining a proper weight (body mass index between 18 and 24 kg/m^2^), physical exercise practice (moderate aerobic activity for 30 minutes per day at least 5 times per week), SAH control, treatment of diabetes and dyslipidemia (I, B).
- Angiotensin-converting-enzyme inhibitors (ACEI) or angiotensin receptor blockers (ARB) are the drugs of choice to treat SAH. Statins are recommended to treat dyslipidemia, aiming at maintaining LDL levels below 100 mg/dL. Metformin is the drug of choice to treat diabetes and, when HF is associated, SGLT2 inhibitors (empaglifozin, dapaglifozin, canaglifozin) should be used. In the presence of coronary artery disease (CAD), GLP-1 agonists (liraglutide, dulaglutide and semaglutide) should be preferred (IIa, C).
- When assessing the therapeutic proposal, the risk factors for cardiotoxicity should be identified and specific measures implemented according to the regimen (IIa, C).
- For patients with subclinical cardiotoxicity [troponin elevation or SLG reduction (absolute ≥ 5% or relative ≥ 15%)]:
  - the use of ACEI or ARB or beta-blocker can be considered, aiming at preventing ventricular dysfunction and cardiovascular events (IIa, B);
  - repeat strain echocardiography every 3 months and measurement of biomarkers every cycle, if asymptomatic, or at any time, if symptoms appear (IIa, C);
  - chemotherapy should not be suspended based on alterations in strain and biomarkers (IIa, C);
    consider referring the patient to the cardio-oncologist (IIa, C);
  - consider ruling ischemic heart disease out (IIa, C);
  - consider initiating dexrazoxane in patients who will undergo high doses of anthracyclines and at high risk for cardiotoxicity (IIa, B).
- For patients with LVEF ≤ 50% and ≥ 40%, therapy with ACEI/ARB and beta-blocker is recommended before initiating a cardiotoxic treatment (I, A).
- Patients with LVEF ≤ 40% should not receive therapy with anthracycline unless there is no effective treatment option (IIa, A).
- Patients on chemotherapy or immunotherapy, who develop HF and LVEF < 40% during treatment, should have their antineoplastic treatment suspended temporarily based on the discussion with the cardiologist and the oncologist, and therapy for HF should begin based on guidelines and consensus statements (I, A).
- Patients on potentially cardiotoxic drugs, who develop HF signs or symptoms, should be referred to the cardio-oncologist for clinical assessment, echocardiography, and measurement of biomarkers (IIa, C).
- Figures 7 and 8 show the algorithms that should be considered for the management of ventricular dysfunction induced by anthracyclines and anti-HER2 (IIa, B).
- For patients with trastuzumab-induced cardiotoxicity, after symptom stabilization and LVEF recovery to > 40%, trastuzumab reintroduction should be considered, provided that the patient is followed up by a cardio-oncologist, with serial assessment by use of echocardiography and biomarkers (IIa, B).
- For patients with trastuzumab-induced cardiotoxicity, if symptoms do not improve and LVEF persists below 40%, trastuzumab should only be reintroduced if there is no therapeutic alternative and after thorough discussion with the oncologist (IIa, C).
- For patients on sunitinib or other anti-VEGF drug, SAH assessment and proper control are recommended (IIa, C).
- For patients on monoclonal antibodies or anti-VEGF tyrosine kinase inhibitors (bevacizumab, sunitinib, sorafenib, axitinib and pazopanib), the highest risk for HF occurs at the beginning of therapy. In the presence of signs and symptoms, the patient should be assessed with echocardiography and measurement of biomarkers (IIa, B). Cardio-oncologist consultation, HF treatment initiation, and discussion of drug suspension with the oncologist are recommended (IIa, C). After clinical and LVEF recovery, consider resuming chemotherapy (IIa, C).
- For patients with HF or ventricular dysfunction, drug treatment should be instituted according to guidelines (I, A).
- The indication for circulatory assistance device or heart transplantation follows the Brazilian Guideline on Acute and Chronic Heart Failure recommendations. Before the indication, the patient's *status* and oncological prognosis should be discussed with the oncologist, always considering the patient's preferences.
- The indication for heart transplantation of cancer patients follows the Brazilian Guideline on Acute and Chronic Heart Failure recommendations (Table 8). However, patients with acute or chronic HF should only be considered for transplantation when meeting criteria of cancer remission or cure for more than 3 years (IIa, C).
- If myocarditis due to ICIs is suspected or confirmed, the therapy with ICIs should be interrupted and corticosteroid initiated immediately (intravenous methylprednisolone, 1g per day, for 3 to 5 days, followed by prednisone, 1-2 mg/kg/day). Corticosteroid should be kept until resolution of the symptoms and normalization of troponin, systolic function and conduction abnormalities (IIa, C). In cases of pericarditis, oral corticosteroid is recommended (IIa, C). For Takotsubo syndrome, pulse therapy can be considered (IIa, C), and, for dilated cardiomyopathy, support treatment is recommended (Table 9).
- For patients with refractory myocarditis or in severe situations with cardiogenic shock, other immunosuppressant therapies, such as antithymocyte globulin, infliximab (except for patients with HF), mycophenolate mofetil, cyclophosphamide or abatacept, should be considered (IIa, C).
- For patients with tachyarrhythmia or bradyarrhythmia induced by ICIs, proper drug therapy and pacemaker should be considered according to the clinical characteristics (IIa, C).
- Therapy with ICIs should be discontinued in cases of myocarditis. The decision on resuming therapy should be individualized according to cancer *status*, response to treatment and cardiotoxicity severity, and analyzing the risks and benefits. If ICIs are resumed, monotherapy with one anti-PD1 drug and cardiovascular surveillance are recommended (IIa, C).
- Consider the use of dexrazoxane for patients with metastatic breast cancer and a planned high dose of anthracycline (doxorubicin > 250 mg/m^2^) (I, A), for patients with sarcoma and for pediatric patients with lymphoma/leukemia (IIa, A).

**Figure 7:** Algorithm for the management of heart failure and ventricular dysfunction induced by anthracyclines. chemo: chemotherapy; HF: heart failure; LVEF: left ventricular ejection fraction

**Figure 8:** Algorithm for the management of heart failure and ventricular dysfunction induced by anti-HER2 therapy. chemo: chemotherapy; HF: heart failure; LVEF: left ventricular ejection fraction

**Table 8:** Recommendations for heart transplantation. Coordinating Committee of the Brazilian Guideline on Acute and Chronic Heart Failure^69^

Recommendation	Class	Level of evidence
For patients with acute heart failure and/or cardiogenic shock with low recovery potential, assessment of heart transplant candidacy should begin early and be as thorough as possible, including psychosocial assessment, even in the presence of difficulties inherent in acute findings.	I	C
For patients with refractory cardiogenic shock and no proper myocardial function recovery, heart transplant candidacy should consider the hemodynamic instability grade, presence of multiorgan dysfunctions, comorbidities, and the transplant center experience. Prognostic scores might help estimate the post-transplant mortality risk in the short and long run.	IIa	C

**Table 9:** Adverse effects of the use of immune checkpoint inhibitors and therapeutic strategies

Potential CV events related to ICIs	Diagnostic methods	Potential initial approach for treatment	Potential additional therapy if stable and not responding to initial approach	Potential additional therapy if unstable
Myocarditis	Non-invasive: CMRI, troponin, ECG Invasive: biopsy and pathology	Methylprednisolone, 1g/day for 3-5 days, followed by prednisone, 1.5mg/kg, with ambulatory troponin monitoring. Standard therapy for HF with neuro-hormonal blocker, if LVEF is reduced.	Mycophenolate, 500-750mg 2x/day Plasmapheresis Intravenous immunoglobulin	Antithymocyte globulin Abatacept Alemtuzumab Mechanical circulatory support
Pericarditis	Non-invasive: echocardiography Invasive: fluid analysis	Prednisone, 1.5mg/kg/day, with ambulatory dose reduction for 2 months.	Methylprednisolone, 1g/day for 3-5 days Mycophenolate, 500-750mg 2x/day	Pericardial drainage if huge pericardial effusion with signs of hemodynamic instability is present
Takotsubo syndrome	Non-invasive: echocardiography, CMRI Invasive: coronary cineangiography and ventriculography	Standard therapy for HF with neuro-hormonal blocker, if LVEF is reduced. Consider methylprednisolone, 1g/day for 3-5 days, followed by oral prednisone with dose reduction for 4-6 weeks.	Mycophenolate, 500-750mg 2x/day	Mechanical circulatory support
Dilated cardiomyopathy	Non-invasive: CMRI, echocardiography, troponin, natriuretic peptide Invasive: coronary cineangiography and ventriculography	Standard therapy for HF with neuro-hormonal blocker, if LVEF is reduced.	Cardiac resynchronization therapy Implantable cardioverter defibrillator	

*This table details the CV toxicities associated with ICIs and potential management strategies. Many strategies listed for toxicities other than myocarditis have been extrapolated from the literature on myocarditis and based on small case series or case reports. CMRI: cardiac magnetic resonance imaging; CV: cardiovascular; ECG: electrocardiogram; HF: heart failure; ICIs: immune checkpoint inhibitors; LVEF: left ventricular ejection fraction. Table adapted from: Lenihan DJ et al. Proceedings*.^18^

## 7. Arterial and Venous Thromboembolism

Thromboembolic disease is common in cancer patients, being considered the second cause of mortality in that population.

### 7.1. Venous Thromboembolism

Venous thromboembolism (VTE) comprises deep venous thrombosis (DVT) and pulmonary thromboembolism (PTE). It is a severe complication in cancer patients, being their second cause of death. Neoplasms are associated not only with an increase in the risk for VTE and in its severity, but also with thrombosis recurrence, which result in higher rates of treatment-related complications. Moreover, cancer patients have a 2- to 9-times higher chance of recurrence of thromboembolic events.^70^^–^^72^

Cancer induces a pro-thrombotic state because of the following: production of thrombogenic microparticles; platelet activation; its antifibrinolytic properties; and thrombin production. In addition, thrombogenesis is intensified by factors related to cancer type, disease *status*, concomitant use of drugs,^73^ such as erythropoiesis-stimulating agents, presence of anemia and leukocytosis, obesity and thrombogenic laboratory phenotype, such as high levels of D-dimer and prothrombin fragment 1+2.^74^

In the past 5 years, some clinical trials on the cancer population were published, allowing the expansion of the therapeutic arsenal (Table 10).^75^^,^^76^

**Table 10:** Clinical studies on venous thromboembolism in cancer patients

Study	Population	Intervention	Primary efficacy outcomes	Primary safety outcome
**Primary prevention**				
CASSINI trial*	841 ambulatory cancer patients at high risk for VTE	Rivaroxaban 10mg vs placebo 6 months	DVT or PE or VTE-related death HR: 0.66; 95%CI: 0.4-1.09	Major bleeding 1.0% vs 2.0% HR: 1.96; 95%CI: 0.59-6.49
AVERT trial*	574 ambulatory cancer patients at high risk for VTE	Apixaban 2.5mg 2x/day vs placebo	Documented VTE 4.2% vs 10.2% HR: 0.41; 95%CI: 0.26-0.65	Major bleeding 3.5% vs 1.8% HR: 2.0; 95%CI: 1.0-3.95
Treatment				
HOKUSAI VTE	1050 cancer patients with acute symptomatic or incidental VTE	LMWH for 5 days + edoxaban 60mg vs dalteparin Treatment: 6 months	VTE recurrence or major bleeding 12.8% vs 13%	Major bleeding 6.9% vs 4% HR: 1.77; 95%CI: 1.03-3.0^4^
SELECT-D	406 cancer patients with symptomatic PE or VTE	Rivaroxaban vs dalteparin Treatment: 6 months	VTE recurrence: 4% vs 11% HR: 0.43; 95%CI: 0.19-0.99	Major bleeding 6% vs 4% HR:1.83; 95%CI: 0.68-4.96
ADAM-VTE trial	300 patients with VTE associated with cancer	Apixaban 10mg 2x/day for 7 days followed by 5mg 2x/day vs dalteparin	VTE recurrence: 0.7% vs 6.3% HR: 0.099; 95%CI: 0.013-0.78	Major bleeding 0% vs 1.4% HR: 1.96; 95%CI: 0.59-6.49
Caravaggio Study*	1055 cancer patients with symptomatic or incidental VTE or PE	Apixaban 10mg for 10 days followed by 5mg/day vs dalteparin	VTE recurrence: 5.6% vs 7.9% HR: 0.63; 95%CI: 0.37-1.07	Major bleeding 3.8% vs 4% HR: 0.82; 95%CI: 0.4-1.69

* Randomized clinical trial; CASSINI = rivaroxaban in ambulatory cancer patients at high risk for VTE; AVERT = apixaban for VTE prevention in cancer patients; HOKUSAI VTE = edoxaban *versus* dalteparin to treat symptomatic VTE; SELECT-D = anticoagulation in patients at high risk for VTE recurrence; ADAM VTE = apixaban and dalteparin in VTE associated with active neoplasm; Caravaggio Study = apixaban to treat VTE associated with cancer. CI: confidence interval; DVT: deep venous thrombosis; HR: hazard ratio; LMWH: low-molecular-weight heparin; PE: pulmonary embolism; VTE: venous thromboembolism; Table adapted from: Lenihan DJ *et al*. Proceedings.^18^

The recommendations for VTE management in cancer patients are:

- The multiprofessional team caring for oncological patients should instruct them on the risk for VTE, particularly in high-risk situations, such as large surgeries and during chemotherapy (IIa, C).
- In-patients should receive pharmacological prophylaxis, in the absence of contraindications (IIa, B).
- Pharmacological prophylaxis should not be routinely performed for patients admitted for small procedures or chemotherapy infusion or transplantation (IIa, C).
- For low-risk outpatients, routine anticoagulation to prevent VTE is not recommended (III, B).
- Outpatient pharmacological prophylaxis with apixaban, rivaroxaban or enoxaparin should be provided to those at high risk for VTE, assessed with the Khorana score (≥ 2) or the CAT score (D-dimer level and cancer type) (IIa, A).
- For outpatient pharmacological prophylaxis assessment, consider the patients' risk for bleeding (higher in gastrointestinal tumors) and their preferences (IIa, C).
- Patients with multiple myeloma on thalidomide or lenalidomide or dexamethasone should be assessed for the use of aspirin or enoxaparin (IIa, C).
- Patients undergoing large oncological surgeries should receive pharmacological prophylaxis against VTE (enoxaparin or low-molecular-weight heparin), starting in the preoperative period, except for those with active bleeding or at high risk for bleeding (I, A). Mechanical methods can be added to pharmacological prophylaxis; however, they should only be used as monotherapy for patients with contraindication for heparin (IIa, B).
- The combined regimen of pharmacological and mechanical prophylaxis can improve efficacy, especially in patients at higher risk (IIa, B).
- The pharmacological prophylaxis against thrombus for patients undergoing large oncological surgery should be extended for 7 to 10 days, and be prolonged for 4 weeks in the postoperative period in cases of open abdominal or laparoscopic surgery and of pelvic surgery in the presence of other risk factors, such as obesity, immobility, and history of VTE (IIa, B).
- For smaller surgeries, the duration of prophylaxis should be decided on a personalized basis (IIa, C).
- In the oncology patient, VTE can be initially treated with low-molecular-weight heparin (enoxaparin), unfractionated heparin, fondaparinux, apixaban or rivaroxaban. For patients starting treatment with parenteral anticoagulation, low-molecular-weight heparin, rather than unfractionated heparin, is preferred in the first days of treatment, provided the patient has no kidney dysfunction (creatinine clearance should be > 40 mL/min/m^2^) (I, A).
- Long-term anticoagulation can be performed preferably with low-molecular-weight heparin, edoxaban, apixaban or rivaroxaban for at least 6 months (I, A).
- Warfarin can be used in cancer patients when other drugs are not available or when other anticoagulants are contraindicated, such as for chronic kidney failure requiring dialysis (IIa, B).
- Direct oral anticoagulants (DOACs), such as rivaroxaban and apixaban, are associated with higher bleeding rates, especially in gastrointestinal and genitourinary neoplasms (IIa, B).
- In cancer patients, drug interactions with DOACs should be analyzed on a case-by-case basis (I, A).
- Anticoagulation for more than 6 months should be offered to patients with active cancer, such as metastatic ones, or on chemotherapy, provided the risks and benefits are analyzed (IIa, C).
- Based on expert opinions, in the absence of randomized studies, vena cava filters should not be inserted in patients with chronic or established (for more than 4 weeks) thrombosis or temporary contraindications for anticoagulant therapy (IIa, C).
- Warfarin is the first option for anticoagulation in patients with chronic kidney failure requiring dialysis (IIa, B).
- Vena cava filters can be considered for patients with high-risk acute VTE (in the past 4 weeks) and absolute contraindication for anticoagulation (IIa, C).
- Incidental PTE and DVT should be treated the same way symptomatic VTE is, because they have similar outcomes (IIa, C).
- The treatment of subsegmental PTE or of visceral or splanchnic venous thrombosis should be considered on a case-by-case basis, analyzing the potential benefits and risks of anticoagulation (IIa, C).
- Cancer patients should have their risk for VTE assessed on an outpatient basis with the Khorana or the CAT score, and the benefits and risks of that strategy should be analyzed on a case-by-case basis, because they are associated with a reduction in thromboembolic events but not in mortality (IIa, B).
- For clinically significant bleeding associated with warfarin, the treatment of choice is intravenous vitamin K (10 mg) and intravenous prothrombin complex (500 U/kg) (IIa, B).
- For bleeding associated with rivaroxaban, edoxaban and apixaban, no specific antidote is available. Thus, the use of antifibrinolytics (intravenous tranexamic acid, 1g to 2g) and prothrombin complex (500 U/kg, intravenous) is recommended. For refractory cases, plasma (15 mL/kg), cryoprecipitate (1 U/kg) and platelet (1-2 units), by use of apheresis, are recommended (IIa, C).

There is a substantial variation in the risk for VTE in cancer patients and different clinical situations. Cancer patients should have their risk for VTE analyzed in the baseline assessment and then periodically, particularly at the beginning of antineoplastic therapy and on hospital admission. Individual risk factors, including biomarkers or cancer site, do not accurately identify cancer patients at risk for VTE. On an outpatient basis, the assessment should include the Khorana and the CAT scores (IIa, C) (Tables 11 and 12, respectively).

**Table 11:** Khorana score^77^

Patient's characteristic		Points	
	Site of cancer	
	Very high risk (stomach and pancreas)	2
High risk (lung, lymphoma, gynecological, urinary bladder, testicles, kidney)		1
Pre-chemotherapy platelet count ≥ 350 000 / μL		1
Hemoglobin < 10 g/dL and/or use of erythropoiesis-stimulating agents		1
Pre-chemotherapy leukocyte count > 11 000 / μL		1
Body mass index ≥ 35 kg/m^2^		1
Calculate the total score by adding the points for each criterion of the model		
Score interpretation:		
	High risk: ≥ 3 points	
	Intermediate risk: 1-2 points	
	Low risk: 0 point	

**Table 12:** Nomogram (CAT score) to predict risk for venous thromboembolism in 6 months^78^

### 7.2. Arterial Thrombosis

In an epidemiological study with 279 719 participants and comparing patients with neoplasm and controls without neoplasm, the incidence of arterial events was 4.7% in the former and 2.2% in the latter in 6 months.^79^ Usually, these events occur in individuals with metastatic pancreas, breast, colorectal and lung neoplasms, who are on anthracyclines, taxanes and platins. The pro-thrombotic state may favor the occurrence of embolic events secondary to atrial fibrillation. Some antineoplastic drugs, especially VEGF inhibitors, may induce thromboembolic complications. In patients on hormone therapy, higher rates of arterial thrombotic events are more often observed with aromatase inhibitors than with tamoxifen. In several cases, kinases and their pathways play a critical role in vascular and metabolic cell homeostasis. The inhibition of these kinases can cause cardiovascular sequelae, depending on the kinase type. The most worrisome vascular toxicities that might occur with the new agents include arterial ischemic events, such as acute myocardial infarction, stroke and ischemia of a limb, as well as venous thromboembolic events.^79^

Recent reports have shown that the VEGF-inhibitor therapy results in adverse vascular events, such as aortic dissection, stroke, and arterial and venous thrombosis. Of the VEGF inhibitors, bevacizumab is associated with the highest VTE rate, around 12%, as compared to 2% with the other drugs.^49^^,^^80^

## 8. Metabolic Syndrome Associated with Androgen Deprivation Therapy

The treatment of locally advanced prostate neoplasms is based on the hormonal control of testosterone. This blockade can be obtained surgically (orchiectomy) or through androgen deprivation therapy. Gonadotropin releasing hormone (GnRH) agonists (leuprolide, goserelin and triptorelin) and antagonists (degarelix) cause central blockade with a reduction in the levels of luteinizing and follicle stimulating hormones and testosterone. In addition, adrenal androgen receptor inhibitors (abiraterone) and direct androgen inhibitors (enzalutamide) reduce testosterone. These drugs are used with curative intention in high-risk patients with non-metastatic disease and as standard therapy for metastatic disease. Understanding the impact of these drugs on cardiovascular risk is important because many risk factors that lead to prostate cancer can result in cardiovascular disease, such as advanced age, smoking, diet, and obesity. Some studies have reported a higher prevalence of those risk factors among prostate cancer patients.

The recognized antiandrogen therapy leads to metabolic changes characterized by hyperinsulinemia, hypercholesterolemia, and body composition changes, with an increase in predominantly visceral fat and a reduction in lean mass. The metabolic syndrome resulting from the antiandrogen therapy is associated with an increase in cardiovascular complications. Modification of risk factors is recommended with lipid-lowering therapy, anti-hypertensive treatment, strict control of glycemia, and use of antiplatelet drugs (IIa, B).

## 9. Cardiac Arrhythmia

Several factors present in cancer patients, such as infection, electrolyte imbalance, dehydration, surgical procedures, and oncological and adjuvant therapies, predispose to the occurrence of cardiac arrhythmias.^81^ These arrhythmias are relatively frequent complications in cancer patients, estimated to occur in 16-36% of those patients.^82^^,^^83^

The types of cardiac arrhythmias in oncology patients comprise a wide range: sinus tachycardia, bradyarrhythmias, tachyarrhythmias, and conduction disorders. Of the supraventricular arrhythmias, the most common is atrial fibrillation. Ventricular tachycardia and ventricular fibrillation are rare, but can occur especially in the presence of QT prolongation and in patients with hypokalemia or hypomagnesemia.^84^^,^^85^ Table 13 lists the major drugs related to cardiac arrhythmias and their incidences.

**Table 13:** Major drugs related to cardiac arrhythmias and their incidences

Drug	Incidence
Anthracyclines	ECG changes: 38.6% AF: 2-10%
Antimicrotubule agents (Paclitaxel)	Sinus bradycardia: 29% 1^st^ degree AVB: 25%
Antimetabolites (5-Fluorouracil and Capecitabine)	ECG changes: 68% Arrhythmias (AF, SVT, VT): 5%
Platins (Cisplatin)	SVT: 12-32%
Thalidomide	Bradycardia: 27%
Arsenic trioxide	QT prolongation and ventricular arrhythmias: up to 50%
**Tyrosine kinase inhibitor**	
Crizotinib	QT prolongation and arrhythmias: 3.5%
Dasatinib	QT prolongation and arrhythmias: 0.6-3%
Sunitinib	QT prolongation and arrhythmias: 1-4%
Vandetinib	QT prolongation and arrhythmias: 12-15%

*AF: atrial fibrillation; AVB: atrioventricular block; ECG: electrocardiographic; SVT: supraventricular tachycardia; VT: ventricular tachycardia*.

### 9.1. QT Prolongation

The diagnosis of QT prolongation is electrocardiographic, and QTc should be calculated by use of the Bazett's formula [QT / (RR)^1/2^] or Fridericia's formula [QT / (RR)^1/3^]. Normal QTc values are as follows: ≤ 440 ms in men; between 450 and 460 in women. Both congenital and acquired factors can be responsible for QT prolongation, and the most cited conditions are as follows: female sex, bradycardia, electrolyte abnormalities, drug effects, myocardial ischemia, HF, myocarditis, hypothermia, and channelopathies.^86^

The QT prolongation is a concern in cancer patients, because the oncological treatment, electrolyte disorders and concomitant medications can contribute to that prolongation and predispose to complex arrhythmias.^83^ Monitoring the QT interval and correcting the factors that contribute to QT prolongation should be considered in patients on medications that prolong the QT interval. Cardiotoxicity is defined in the presence of QTc prolongation > 500 ms and/or QT variation > 60ms from baseline (Table 14).^87^^,^^88^

**Table 14:** Recommendations for patients on drugs that can prolong the QT interval

Avoid using drugs related to QTc prolongation for patients with pre-treatment QTc > 470ms
Discontinue drugs related to QTc prolongation if QTc > 500ms, or, if QTc > 550ms when baseline QRS duration is prolonged (> 120ms secondary to pacemaker or bundle-branch block)
Reduce dose or discontinue drug if QTc increases more than 60ms from pre-treatment value
Maintain serum electrolyte (potassium, magnesium, and calcium) concentrations within the normal range
Prevent drug interactions
In patients with acute renal injury or chronic renal disease, adjust the drugs with renal clearance that prolong QTc to kidney function
Avoid rapid intravenous infusion of drugs that prolong QTc
Avoid the concomitant administration of more than one drug that prolongs QTc
Avoid drugs that prolong QTc for patients with history of drug-induced *torsade de points* or patients resuscitated from cardiac sudden death
Avoid drugs that prolong QTc for patients with congenital diseases
Perform ECG at a frequency depending on the therapy, dose administered, and concentration of drugs

*ECG: electrocardiogram; QTc: corrected QT interval. Source: Adapted from Porta-Sanchez et al*.^88^

The QT interval and the risk factors associated with QT prolongation should be assessed before and after treatment with drugs known to be related to cardiac arrhythmias, such as tyrosine kinase inhibitors (crizotinib, dasatinib, sunitinib) and arsenic trioxide. Electrolyte and ECG assessments should be performed during treatment at baseline, 7-15 days after the beginning of therapy, after changes in doses in the first 3 months, and depending on the therapy frequency. Before postponing chemotherapy, the suspension of other medications related to QT prolongation, such as antiemetics, antidepressants, antiarrhythmics, antifungal drugs, antipsychotics, should be considered. In addition, correction of electrolyte disorders should be performed. Patients on arsenic trioxide should be monitored with ECG every week.^22^

The QT prolongation increases the incidence of ventricular arrhythmias and *torsades de points.*^88^ Ventricular tachycardias are usually associated with structural cardiomyopathies [CAD, dilated cardiomyopathy, right ventricular heart diseases, congenital abnormalities, hypertrophic cardiomyopathy, and channelopathies].^89^ The objective of the treatment of ventricular arrhythmias is to reduce morbidity and sudden death events, and the assessment of triggering factors is paramount. Pharmacological therapy is indicated for refractory and/or symptomatic cases.^90^

### 9.2. Atrial Fibrillation

Atrial fibrillation is the most common cardiac arrhythmia of the oncology patient. Its occurrence is related to the pro-inflammatory state of these patients, the inflammatory response to oncological surgery, and the cardiotoxic effects of antineoplastic therapy.^91^ Understanding the mechanisms that trigger and sustain atrial fibrillation is important for prevention.

Atrial fibrillation can be induced by several mechanisms, such as myocardial change due to electrolyte disorder, liposomal and mitochondrial injury, inflammation, pericardial disease, and increased oxidative stress inducing cell apoptosis.^92^

There is great difficulty in establishing the causal relationship between arrhythmic events and each chemotherapy drug. The small number of studies published and the simultaneous administration of many drugs make it difficult to relate drug and effect. The chemotherapy drugs most commonly associated with arrhythmias are anthracyclines (doxorubicin, epirubicin), antimicrotubule agents (paclitaxel and docetaxel), antimetabolites (5-fluorouracil, capecitabine and gemcitabine), alkylating agents (cisplatin and cyclophosphamide), tyrosine kinase inhibitors (ibrutinib, ponatinib, sorafenib and sunitinib), and monoclonal antibodies (trastuzumab and cetuximab), in addition to immunotherapy drugs.

Cancer is associated with a pro-thrombotic state and can increase the risk for embolic events in patients with atrial fibrillation, who also have more bleeding complications due to treatment, and, therefore, higher morbidity and mortality. There is no consensus/guideline-based recommendation on the use of antithrombotic drugs for patients with atrial fibrillation.^85^^,^^93^

The choice of antithrombotic therapy for cancer patients should be individualized, analyzing pharmacokinetic and pharmacodynamic factors, drug interactions, and risks for thrombosis and bleeding (IIa, B).

Warfarin should be avoided in the oncology patient with atrial fibrillation, because that drug is associated with lower efficacy and higher risk of bleeding due to drug interactions, higher occurrence of liver dysfunction, dietary changes, cachexia, and malnutrition (IIa, B).

The DOACs (dabigatran, rivaroxaban, apixaban and edoxaban) are superior to warfarin in terms of efficacy and bleeding in the general population with atrial fibrillation; however, the evidence for their use in cancer patients derives from analyses of substudies and observational data (IIa, B).

Although there is no validation of the classic scores for the cancer population, anticoagulation should be initiated in those patients according to the same criteria adopted for the population without cancer: CHADS_2_ and CHA_2_DS_2_-VASc scores above 2 indicate anticoagulation (IIa, C) (Table 15).

**Table 15:** Risk score of thromboembolism associated with atrial fibrillation

CHA^2^DS^2^-VASc		
	Description	Points
C	Heart failure	1
H	Arterial hypertension	1
A_2_	Age (≥ 75 years)	2
D	Diabetes mellitus	1
S_2_	Previous TIA or stroke	2
V	Vascular disease (prior AMI, peripheral artery disease or aortic plaque)	1
A	Age (65-74 years)	1
Sc	Female sex	1

*AMI: acute myocardial infarction; TIA: transient ischemic attack*.

Antithrombotic therapy in cancer patients should be personalized, analyzing the patient's profile, cancer type, and risk for thrombosis and bleeding, the last, for example, by use of the HAS-BLED score (IIa, C) (Table 16).

**Table 16:** Risk score of bleeding associated with anticoagulation (HAS-BLED). Score 1 for each item

□	Arterial hypertension (1 point)
□	Abnormal liver function (1 point)
□	Abnormal kidney function (1 point)
□	Stroke (1 point)
□	Bleeding tendency or predisposition (1 point)
□	Labile INRs in patients on warfarin (1 point)
□	Elderly: age > 60 years (1 point)
□	Drugs: concomitant antiplatelet agent(s) or NSAIDs (1 point)
□	Alcohol abuse (1 point)
*INR: international normalized ratio; NSAIDs: non-steroidal anti-inflammatory drugs*.	
0 point: 1.02 bleeding per 100 patients/year	
1 point: 1.13 bleeding per 100 patients/year	
2 points: 1.88 bleeding per 100 patients/year	
3 points: 3.74 bleedings per 100 patients/year	
4 points: 8.70 bleedings per 100 patients/year	
points: insufficient data (high risk)	

There is no prospective randomized study on DOACs in cancer patients with atrial fibrillation. Analysis of substudies from randomized clinical trials have shown those drugs to be safe and effective in cancer patients. This evidence and the results from studies on VTE and cancer, which confirm the superiority of DOACs as compared to low-molecular-weight heparin, suggest that DOACs are a feasible option of antithrombotic therapy in cancer patients with atrial fibrillation (IIa, C).

The routine use of DOACs can be considered for patients with atrial fibrillation and gastrointestinal or genitourinary tract tumors, and the potential risk for bleeding should be analyzed (IIb, B).

The management of cancer patients with atrial fibrillation should include a cardiologist since the beginning, because of the higher rates of anticoagulation use and lower incidence of ischemic and hemorrhagic complications (IIa, C).

## 10. Coronary Artery Disease

Cancer and CAD have several risk factors in common and often coexist in the same patient (Figure 9). The presence of risk factors, such as advanced age, smoking, diabetes, SAH, sedentary lifestyle and dyslipidemia, is elevated in cancer patients.^94^ Other common factors in those patients that contribute to the development of CAD are endothelial dysfunction, oxidative stress, genetic predisposition and chronic inflammation.^95^

**Figure 9:** Coronary artery disease in cardio-oncology. CAD: coronary artery disease; DAPT: dual antiplatelet therapy; VEGF: vascular endothelial growth factor

In addition, the oncological treatment contributes to the high prevalence of CAD in cancer patients.^49^ Patients with lung cancer undergoing chemotherapy have a 5.3-time (95% CI: 2.002-14.152) increase in the risk of important coronary injury, which suggests that the treatment may be associated with anatomic complexity.^96^ The major mechanisms of CAD related to oncological therapy are: vasospasm, thrombosis, and accelerated atherosclerosis.^49^

In patients on cisplatin isolated or associated with vincristine or bleomycin, coronary thrombosis has been observed in coronary angiography without previous atherosclerosis. These drugs can induce endothelial dysfunction/lesion, which seems to be the basic mechanism of the vasoactive alteration caused by these drugs.^97^^–^^99^ Cisplatin leads to the death of endothelial cells via the production of procoagulant microparticles.^100^

Another class of chemotherapy drugs typically related to CAD in cancer patients are the antimetabolites, especially 5-fluorouracil and capecitabine. The incidence of angina or acute findings ranges from 3.9% to 12.5%.^101^ The mechanism through which these drugs cause toxicity has not been completely established and several hypotheses have been raised to explain those findings, such as acute vasospasm, direct toxicity to myocytes, endothelial dysfunction, and hypercoagulable state causing thrombosis.^101^^,^^102^ Acute vasospasm is often observed and experimental studies have suggested that vasoconstriction caused by 5-fluorouracil is related to protein kinase C and endothelin-1.^103^^,^^104^ Similarly to 5-fluorouracil, taxanes are another class of drugs that induce angina secondary to coronary spasms. The incidence of chest pain reported by patients on paclitaxel is approximately 0.2-4%.^105^^,^^106^

Accelerated atherosclerosis has been observed in patients being treated with second- and third-generation tyrosine kinase inhibitors. These drugs have an increased risk of coronary occlusion as compared to imatinib (OR = 3.45; 95% CI: 2.30-5.18).^107^ Of the tyrosine kinase inhibitors, ponatinib seems to be the one most often related to vascular toxicity. Arterial and venous thromboses have been reported in 27% of the patients on ponatinib, regardless of the presence of cardiovascular risk factors.^107^ Patients treated with bevacizumab have high risk for coronary ischemia as compared to a control group (RR: 2.47; 95% CI: 1.4-4.36).^108^ Patients on ICIs have an altered inflammatory cell composition of the atherosclerotic plaque (increased ratio of CD3+ T cells to CD68+ macrophages), which may predispose to plaque progression and/or clinical coronary events.^109^

Radiotherapy is classically related to the development of CAD, usually reported later after exposure to radiation. The incidence varies in the literature, with a decreasing tendency in recent decades because of the most modern techniques that reduce direct radiation emission to the heart. The pathogenesis of CAD is multifactorial, resulting in direct myocardial injury, vascular tonus alteration, inflammatory activation, and oxidative stress.^110^^,^^111^

Because of the complexity of cancer patients with CAD, their mortality is higher than that of patients without cancer in the long run.^112^^,^^113^ When approaching those patients, it is important to know the oncological prognosis, the therapeutic perspectives and the program of oncological surgeries. The control of risk factors should be reinforced in patients with evidenced CAD. The use of drug-eluted stent should be preferred in those patients when the therapeutic intervention is indicated.^114^ Therapy with antiplatelet agents should be maintained according to the guidelines on CAD and acute coronary syndrome management, unless contraindicated, such as in the presence of tumor bleeding.^115^^–^^117^

Recommendations:

- The control of risk factors (SAH, diabetes, dyslipidemias) and of body weight loss, smoking cessation and dietary guidance should be provided to all patients who will receive drugs that predispose to CAD (I, A).
- Drug-eluted stent should be preferred in these patients (IIa, B).
- Dual antiplatelet therapy can be maintained in patients with platelet count > 30 000, in the absence of contraindication (IIa, B).
- The investigation of CAD is indicated in patients 5 years after mediastinal exposure to a dose of at least 30 gy (IIa, B).
- For patients who developed acute coronary syndrome with 5-fluorouracil, assessment of the coronary anatomy and by the cardio-oncology team should be considered (IIa, B).
- Re-exposure can be considered for patients with mild events, who are asymptomatic and whose benefit with 5-fluorouracil impacts the prognosis. The use of nitrates and vasodilators can be considered in this scenario (IIb, B).

## 11. Arterial Hypertension

The prevalence of SAH is higher in cancer patients and survivors as compared to that of the general population.^118^ In these patients, SAH is the major modifiable risk factor for cardiovascular events.^119^ Chronic kidney disease, SAH, cardiovascular disease, and cancer have risk factors in common, such as smoking, obesity, and diabetes. Many types of cancer and their treatments cause or aggravate preexisting SAH, because of vascular, endothelial and renal effects.^22^

Periodical blood pressure measurement is recommended in cancer patients (IIa, C).

The selection of anti-hypertensive agents should consider individual risk factors, effects of the antineoplastic treatment and drug interactions. It is estimated that 35% of cancer patients will develop SAH during the treatment. Patients with history of cancer have a higher prevalence of SAH than that of the general population.^94^ Patients with renal, gastric and ovarian cancer have higher blood pressure levels than those with other tumor sites. Exposure to chemotherapy is an independent risk factor for SAH.^120^

### 11.1. SAH and Chemotherapy

Therapy with anti-VEGF tyrosine kinase inhibitors and multi-targeted tyrosine kinase inhibitors aggravates and induces SAH.^121^ The mechanisms are a reduction in the production of nitric oxide and in angiogenesis, leading to an increase in systemic vascular resistance. Anti-VEGF therapy leads to fluid retention because of natriuresis impairment, in addition to inducing endothelin-1-mediated vasoconstriction and thrombotic microangiopathy, similarly to the pathophysiology of eclampsia.^121^ In a recent meta-analysis, the use of anti-VEGF tyrosine kinase inhibitors increased the risk for cardiotoxicity, such as SAH, bleeding and ventricular dysfunction. The most common vascular cardiotoxicity was SAH.^122^

The alkylating agents seem to induce SAH through nephrotoxicity, but there is not much evidence of their real effect on blood pressure. Cyclophosphamide has been associated with multiple vascular complications, such as pulmonary and hepatic veno-occlusive disease, thromboembolic disease, and myocardial ischemia. Pre-clinical evidence has shown endothelial injury and renin-angiotensin-aldosterone system abnormalities in animals treated with cyclophosphamide.^21^^,^^22^ Both ifosfamide and cisplatin apparently induce SAH by causing nephrotoxicity.^123^ The antimicrotubule agents affect mitosis, acting on tubulin to prevent microtubule polymerization. Experimental studies have shown that vinblastine acts on the endothelium and apoptosis, but its effect in SAH is unkonwn.^124^^,^^125^ Gemcitabine and proteasome inhibitors can trigger SAH associated with thrombotic microangiopathy. Adjuvant medications associated with SAH used for cancer patients are: corticosteroids, erythropoietin, calcineurin inhibitors, and anti-inflammatory drugs.

### 11.2 Cancer-Induced SAH

Systemic arterial hypertension can be a paraneoplastic manifestation of hepatocellular carcinoma, renal cancer, and carcinoid disease. It results from the production of renin, angiotensinogen, angiotensin I or catecholamines. Among individuals with renal cell carcinoma, the SAH prevalence exceeds 75%, and SAH is due to the nephrectomy-related loss of nephrons and particularly to the treatment with VEGF inhibitors. In addition, renal cell carcinoma can secrete vasoactive peptides, mainly endothelin-1. The presence of SAH in renal cell carcinoma may indicate more aggressive disease, with a negative impact on prognosis.^126^^,^^127^ Pheochromocytoma and paraganglioma are neuroendocrine tumors of the chromaffin cells, with an annual incidence of 0.8 per 100 000 individuals. The SAH related to those tumors is caused by secretion of catecholamines (norepinephrine, epinephrine, and dopamine) and can be associated with symptoms, such as headache, palpitations, and sweating.^128^

Patients with cancer and SAH have a higher incidence of heart and kidney failures, and SAH is an independent risk factor for CAD, HF, and arrhythmia, being the major modifiable risk factor to prevent HF.^47^^,^^129^

Blood pressure should be properly and regularly measured in cancer patients (I, A).

Patients on anti-VEGF tyrosine kinase inhibitors, multi-targeted tyrosine kinase inhibitors, alkylating agents or high doses of steroids should undergo more frequent blood pressure monitoring (IIa, C).

The blood pressure goal in cancer patients follows the recommendations for patients without cancer: < 130 x 80 mm Hg (IIa, B).

The drug treatment should be personalized, but the presence of proteinuria or ventricular dysfunction determines the indication of ACEI or ARB (IIa, B).

If there is neither proteinuria nor ventricular dysfunction, a dihydropyridine calcium-channel blocker (amlodipine) can be initiated (IIa, C).

Diuretics should be used following a well-defined criterion and considering the risk for hypovolemia as well as for fluid and electrolyte imbalance (IIa, C).

Non-dihydropyridine calcium-channel blockers (verapamil and diltiazem) in cancer patients (III, B). Because these drugs are metabolized via CYP3A4, they can alter the serum levels of antineoplastic drugs.

Secondary causes of SAH, such as hypovolemia and pain, should be investigated (IIa, C).
